# Multi-color live-cell STED nanoscopy of mitochondria with a gentle inner membrane stain

**DOI:** 10.1073/pnas.2215799119

**Published:** 2022-12-19

**Authors:** Tianyan Liu, Till Stephan, Peng Chen, Jan Keller-Findeisen, Jingting Chen, Dietmar Riedel, Zhongtian Yang, Stefan Jakobs, Zhixing Chen

**Affiliations:** ^a^College of Future Technology, Institute of Molecular Medicine, National Biomedical Imaging Center, Beijing Key Laboratory of Cardiometabolic Molecular Medicine, Peking University, Beijing 100871, China; ^b^Peking-Tsinghua Center for Life Science, Academy for Advanced Interdisciplinary Studies, Peking University, Beijing 100871, China; ^c^Department of NanoBiophotonics, Max Planck Institute for Multidisciplinary Sciences, Göttingen 37077, Germany; ^d^Clinic of Neurology, University Medical Center Göttingen, Göttingen 37075, Germany; ^e^Peking University-Nanjing Institute of Translational Medicine, Nanjing 211800, China; ^f^Genvivo Biotech, Nanjing 211800, China; ^g^Fraunhofer Institute for Translational Medicine and Pharmacology, Translational Neuroinflammation and Automated Microscopy, Göttingen 37075, Germany; ^h^Laboratory of Electron Microscopy, Max Planck Institute for Multidisciplinary Sciences, Göttingen 37077, Germany; ^i^Cluster of Excellence “Multiscale Bioimaging: from Molecular Machines to Networks of Excitable Cells”, University of Göttingen, Göttingen 37099, Germany

**Keywords:** mitochondria, STED nanoscopy, cristae

## Abstract

Capturing mitochondria’s intricate and dynamic structure poses a daunting challenge for optical nanoscopy. Different labeling strategies have been demonstrated for live-cell stimulated emission depletion (STED) microscopy of mitochondria, but orthogonal strategies are yet to be established, and image acquisition has suffered either from photodamage to the organelles or from rapid photobleaching. Therefore, live-cell nanoscopy of mitochondria has been largely restricted to two-dimensional (2D) single-color recordings of cancer cells. Here, by conjugation of cyclooctatetraene (COT) to a benzo-fused cyanine dye, we report a mitochondrial inner membrane (IM) fluorescent marker, PK Mito Orange (PKMO), featuring efficient STED at 775 nm, strong photostability, and markedly reduced phototoxicity. PKMO enables super-resolution (SR) recordings of IM dynamics for extended periods in immortalized mammalian cell lines, primary cells, and organoids. Photostability and reduced phototoxicity of PKMO open the door to live-cell three-dimensional (3D) STED nanoscopy of mitochondria for 3D analysis of the convoluted IM. PKMO is optically orthogonal with green and far-red markers, allowing multiplexed recordings of mitochondria using commercial STED microscopes. Using multi-color STED microscopy, we demonstrate that imaging with PKMO can capture interactions of mitochondria with different cellular components such as the endoplasmic reticulum (ER) or the cytoskeleton, Bcl-2-associated X protein (BAX)-induced apoptotic process, or crista phenotypes in genetically modified cells, all at sub-100 nm resolution. Thereby, this work offers a versatile tool for studying mitochondrial IM architecture and dynamics in a multiplexed manner.

## Significance

Mitochondrial nanoscopic imaging has developed from proof-of-principle demonstrations to a viable approach for structural and functional research. The next technological leap requires a palette of orthogonal dyes to label various mitochondrial components and unveil their interactive dynamics in 3D space and extended time, at sub-100 nm resolution. We report a mitochondrial inner membrane fluorescent marker, PK Mito Orange (PKMO), featuring markedly reduced phototoxicity for time-lapse imaging, and compatibility with not only commercial stimulated emission depletion (STED) nanoscopes, but also green and far-red fluorophores such as the genetically encoded calcium indicator (GCaMP) and (silicon rhodamine) SiR. Gentle PKMO labeling enables 3D reconstruction of crista structures in live mitochondria, analyzing crista morphology in live primary cells and genetically edited cell lines, and multiplexed recording of mitochondrial dynamics and interactions.

Mitochondria are the powerhouses of the cell and influence key signaling pathways of cell homeostasis, proliferation, and death (1, 2). Due to their dynamic behavior and abundant interactions with other organelles, mitochondrial research has been particularly driven by the development of fluorescence microscopy (3). However, the delicate double-membrane structure of mitochondria remains invisible using conventional fluorescence microscopes featuring a resolution limit of roughly 200 nm. Surrounded by a smooth outer membrane, the contiguous mitochondrial IM forms numerous lamellar to tubular cristae, membrane invaginations that enhance the overall surface of the IM (4, 5). Crista junctions (CJs), small structures with a diameter of about 20 nm, connect the invaginations to the residual part of the IM and anchor the cristae along the organelle. Cristae are densely stacked along the mitochondrial tubules of most cell types, which can lead to crista-to-crista distances of below 100 nm (6, 7). Due to this intricate arrangement of the cristae, electron microscopy of fixed specimens has been the only tool to capture the unique mitochondrial membrane architecture for decades. However, STED nanoscopy and structured illumination microscopy (SIM) recently allowed to image cristae also in living cells, with the former offering a better spatial resolution of around 40 to 50 nm (3, 8–10) and the latter giving rise to faster imaging recording and longer imaging durations at about 100 to 120 nm resolution (11).

Like all nanoscopy techniques, STED nanoscopy relies on suitable fluorophores to reach its full potential. In the past several years, a handful of new mitochondrial labels facilitated the first live-cell nanoscopic captures of mitochondrial cristae and revealed their dynamic behavior (6, 12, 13). STED nanoscopy using MitoPB Yellow, COX8A-SNAP-SiR, or MitoESq 635 all have demonstrated sub-100 nm resolution imaging of the IM (6, 12, 14). MitoPB Yellow (λ_ex_ = 488 mm, λ_STED_ = 660 nm) and MitoESq (λ_ex_ = 633 mm, λ_STED_ = 775 nm) are mitochondria-targeting, lipophilic dyes with remarkable photostability for time-lapse recordings. Arguably, the widespread use of these two dyes has so far been prevented by phototoxicity or by the lack of combinability with other STED dyes. A different approach utilized the self-labeling SNAP-tag targeted to the IM for subsequent labeling using a SiR dye (SNAP-Cell 647-SiR, λ_ex_ = 633 nm, λ_STED_ = 775 nm) (15). Different to the membrane stains, the latter labeling strategy is generally applicable to the imaging of various mitochondrial proteins (6, 16–18), but it requires genetic manipulation and often involves overexpression of the fusion proteins. STED nanoscopy of mitochondria labeled by SiR causes low photodamage (19) but suffers from rapid photobleaching during image acquisition, which strongly restricts the number of recordable frames when used to label cristae (6, 16).

From a technological point of view, the next challenge in nanoscopic live-cell imaging of mitochondrial dynamics is to facilitate long-term time-lapse imaging, 3D analysis, and multiplexed recordings. To meet these demands, the next generation mitochondrial marker should feature: 1) simple and robust protocol of highlighting mitochondrial structures in various cells and tissues; 2) high brightness and photostability, compatibility with a 775-nm STED laser which is available at most commercial STED microscopes, and compatibility with popular orthogonal nanoscopy dyes such as SiR for multi-color analysis; and 3) reduced phototoxicity to retain the integrity of mitochondria even under strong illumination.

As nanoscopy techniques generally require higher light doses than diffraction-limited approaches, photodamage and photobleaching can become a key, yet often under-evaluated technical hurdle, for analyzing four-dimensional dynamics (19–21). We previously demonstrated that cyanine-COT conjugates (22–26) are gentle mitochondrial markers that allow prolonged SIM recordings of cristae (25) (*SI Appendix*, Fig. S1).

Here, we extend the palette of COT-conjugates, introducing PKMO, an orange-emitting inner membrane stain with minimal phototoxicity. PKMO is photostable and well-tailored for most commercial STED microscopes. The COT conjugated to PKMO depopulates its triplet state, markedly reducing the photodynamic damage during STED imaging. We demonstrate single-color time-lapse STED recordings of mitochondrial dynamics over the time course of several minutes as well as 3D STED recordings of live mitochondria in cultivated cells. We demonstrate that PKMO can be combined with widely used fluorescent labels, enabling simultaneous localization of cristae along with mitochondrial protein complexes, mitochondrial DNA (mtDNA), or cellular organelles like the ER using multi-color nanoscopy. By resolving different crista morphologies in living cells, we demonstrate that PKMO can pave the way for nanoscopy-based chemical and genetic screenings on mitochondria.

## Results

### PKMO Is an Orange-Emitting Cyanine-COT Conjugate that Stains the Mitochondrial Inner Membrane.

**Fig. 1.**
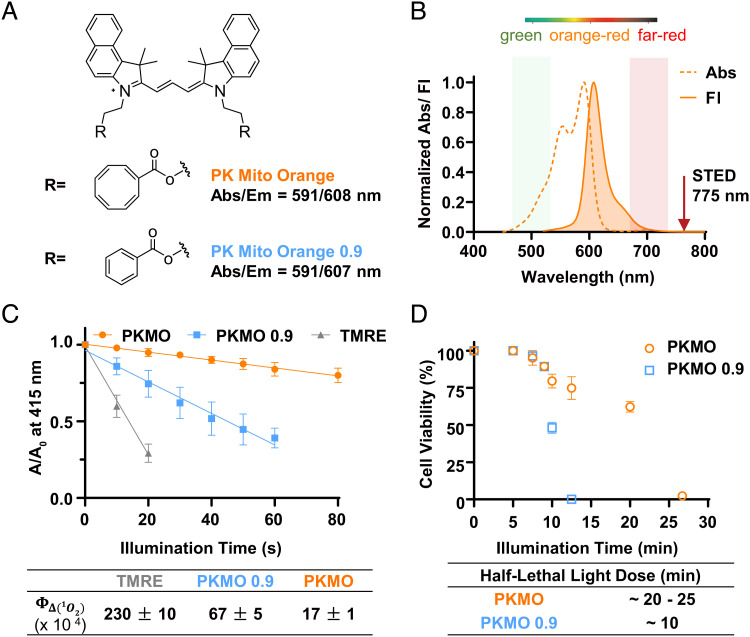
PKMO is a cyclooctatetraene-conjugated Cy3.5, featuring excellent photostability and remarkably low singlet oxygen generation. *A*. Chemical structures of PKMO and a benzoate-derived control compound. *B*. Absorption and fluorescence spectra of PKMO in orange–red channel. The fluorescence can be potentially depleted using a 775-nm laser. *C*. Singlet oxygen quantum yields of PKMO and PKMO 0.9 measured using 1,3-diphenylisobenzofuran (DPBF) decay assay in acetonitrile. TMRE in MeOH (Φ_Δ_= 0.012) was selected as a standard. *D*. Viability of PKMO (1 μM)- and PKMO 0.9 (0.65 μM)-stained HeLa cells after green LED light illumination (543-nm, 2.6 W/cm^2^). >500 cells were examined in each time point of the three independent experiments.

In our previous work, we introduced the two mitochondrial probes PK Mito Red (PKMR) and PK Mito Deep Red (PKMDR) which showcased low phototoxicity due to the use of the COT-conjugating strategy (25). PKMDR features an emission spectrum compatible with the more common 775-nm STED depletion, but the Cyanine 5 (Cy5) scaffold of PKMDR is prone to photobleaching compared to Cyanine 3 (Cy3) (27), giving only ~10 informative STED frames (*SI Appendix*, Fig. S1). In addition, PKMDR cannot be combined with popular live-cell-compatible STED labels such as SiR due to overlapping emission spectra. To overcome this problem, we conjugated COT to Cy3.5 introducing the orange-emitting mitochondrial probe PKMO, which is photostable, well-suited for use with 775-nm STED lasers, and enables multicolor super-resolution (SR) imaging combined with green and far-red emitting probes. The chemical structures of PKMO and its analog without COT, PKMO 0.9, are shown in Fig. 1*A*. Notably, these molecules feature a straightforward synthesis following the well-established cyanine chemistry (<7 steps from commercial material, see *SI Appendix* for details), facilitating their large-scale production. The absorption and emission spectra of PKMO (Fig. 1*B*) demonstrate that the dye is tailored for STED nanoscopy using 561 nm excitation and 775 nm depletion, a configuration implemented in the most commercially available STED microscopes (*SI Appendix*, Fig. S2*A*). Photobleaching of fluorophores is often a significant issue in STED nanoscopy. In hydrophobic poly (methyl methacrylate) film, PKMO exhibited excellent photostability (*SI Appendix*, Fig. S2*C*) compared to popular orange–red dyes such as tetramethylrhodamine ethyl ester (TMRE). Interestingly, PKMO 0.9 appeared to be only slightly less photostable than the COT-conjugated PKMO under this condition (*SI Appendix*, Fig. S2*C*). Nevertheless, PKMO led to significantly reduced singlet oxygen generation in vitro compared to PKMO 0.9 or TMRE as measured by the 1,3-diphenylisobenzofuran decay assay (28) under illumination with a green light-emitting diode (50 mW/cm^2^, 520 to 530 nm) (Fig. 1*C* and *SI Appendix*, Fig. S2*E* and S2*F*) (29). The singlet oxygen quantum yield of PKMO was measured as 1.7 ± 0.1 × 10^−3^, which is ~fourfold lower than that of PKMO 0.9 (6.7 ± 0.5 × 10^−3^). To assess phototoxicity at the cellular level, we labeled live HeLa cells with 1 µM PKMO or 0.65 µM PKMO 0.9 (to achieve the same staining brightness, *SI Appendix*, Fig. S3) and illuminated the cells for different periods in a high-content imager before assessing apoptosis using a Calcein AM stain. The half-lethal light dose for cells stained with PKMO 0.9 was reached after ~ 10-min illumination, whereas for PKMO, the dose was reached after 20 to 25 min, supporting the notion that COT conjugation significantly reduces cellular photodamage induced by long-term illumination. These data corroborate previous results with Cy3/Cy5 (25, 30), suggesting that phototoxicity and photostability are two related yet different effects of excited fluorophores. We conclude that the COT-conjugated PK Mito dyes are primarily characterized by reduced phototoxicity.

For a characterization of the biocompatibility of PKMO, we next investigated the influence of PKMO on dehydrogenase activity, mitochondrial network morphology, and mitochondrial respiration. Measurements of cellular dehydrogenase activity using cell counting kit -8 assays indicated that treatment of HeLa cells for 12 h with up to 1 µM PKMO did not cause a significant reduction in dehydrogenase activity (*SI Appendix*, Fig. S4). We next tested whether PKMO could interfere with mitochondrial fusion and fission dynamics. To this end, we analyzed the overall mitochondrial network morphology whose appearance is the consequence of a delicate balance of fusion and fission events (31). We performed a direct comparison of PKMO, MitoTracker Red, TMRE, and PKMR and stained HeLa cells stably expressing COX8A-mNeonGreen to highlight the mitochondria with 250 nM of the respective red/orange mitochondrial probe. Confocal fluorescence microscopy recordings demonstrated that PKMO delivers specific mitochondrial staining comparable to widely established mitochondrial probes (*SI Appendix*, Fig. S5 *A* and *B*). Individual cells and their respective mitochondrial networks were automatically segmented and analyzed (*SI Appendix*). Neither PKMO nor the other tested probes negatively affected the overall mitochondrial shape or branch length (*SI Appendix*, Fig. S5 *A*, *C* and *D*). Mitochondrial respiration is another sensitive indicator of mitochondrial function. In line with the previously described findings, measurements of the real-time oxygen consumption rate (OCR) using the Seahorse analyzer indicated that the basal, adenosine triphosphate-coupled, proton leak-related, and maximal OCR was not significantly influenced by 250 nM PKMO treatment for up to 12 h (*SI Appendix*, Fig. S6). Together, these data established PKMO as a suitable nontoxic reagent for mitochondrial analysis in live cells.

Most mitochondrial probes accumulate in mitochondria based on the mitochondrial membrane potential (MMP). In line with this, we found that PKMO was partially released from mitochondria following treatment with 10 μM of the membrane potential uncoupler carbonyl cyanide-p-trifluoromethoxy phenylhydrazone. However, the kinetics of the observed signal loss was not as fast and the degree of signal loss not as strong as observed for TMRE, which is known as a real-time MMP indicator (*SI Appendix*, Fig. S7). Therefore, PKMO staining is somewhat dependent on mitochondrial potential but it is not recommended for real-time monitoring of the membrane potential.

### Long-Term Time-Lapse Recording and 3D-STED Imaging of Cristae with PKMO.

**Fig. 2.**
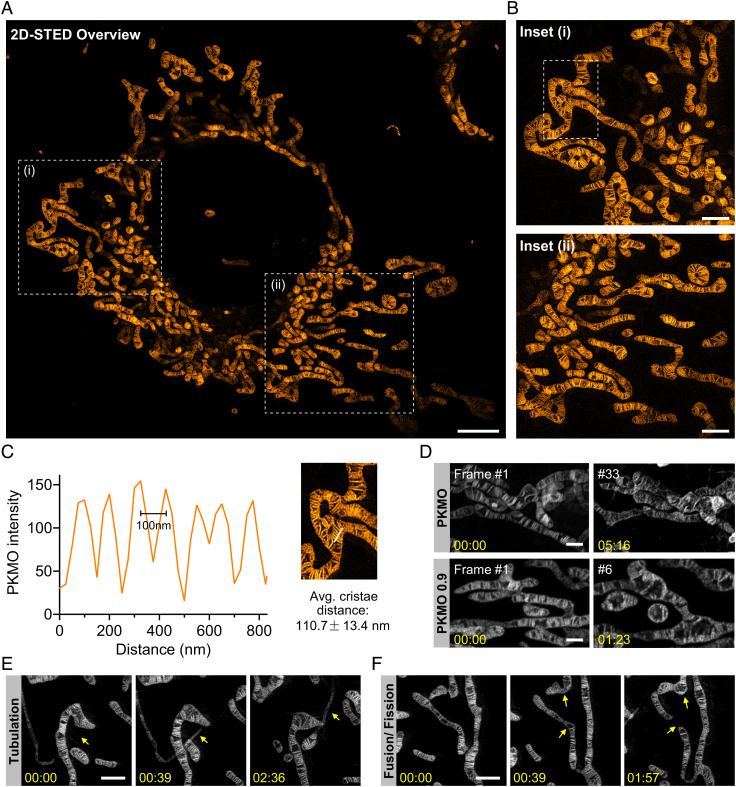
PKMO enables long time-lapse nanoscopic recordings of mitochondrial cristae in COS-7 cells with minimal phototoxicity. *A*. 2D-STED recording of mitochondrial cristae of a COS-7 cell labeled with PKMO. (Scale bar, 10 μm.) *B*. Magnified images of the white boxed areas (i, ii) in *A*. *C*. Fluorescence intensity line profiles measured as indicated in the magnified view of white boxed area in *B*. *D.* Time-lapse recordings of live COS-7 cell labeled with PKMO and PKMO 0.9; PKMO maintained both fluorescent signal and mitochondrial morphology during 30 frames of STED recording, while PKMO 0.9 caused visible mitochondrial swelling after 10 frames. *E*. Time-lapse STED nanoscopy recordings highlighting mitochondrial tubulation. (Scale bar, 1 μm.) *F*. Time-lapse STED nanoscopy recordings showing mitochondrial network dynamics such as fusion and fission. (Scale bar, 1 μm.) All the image data were deconvoluted and image series were corrected for photobleaching.

To test the application of PKMO in SR imaging, we stained COS-7 cells using 250 nM PKMO, resulting in brightly fluorescent mitochondria. PKMO showed excellent performance when recorded with commercial STED nanoscopes (Facility Line and STEDYCON, Abberior Instruments) equipped with 561 nm excitation and 775-nm depletion lasers, and like PKMR, the lipophilic and cationic PMKO specifically accumulated in the IM of mitochondria. We were able to record the mitochondrial networks across entire COS-7 cells and to capture individual mitochondrial cristae (Fig. 2*A*) at an optical resolution of down to 50 nm (*SI Appendix*, Fig. S8). STED recordings revealed that mitochondria in COS-7 cells featured a highly ordered lamellar crista architecture across the entire mitochondrial network (Fig. 2 *A* and *B*). We measured the crista-to-crista distance from a selected area in Fig. 2*B* and found distances of around 100 nm between closely spaced cristae (Fig. 2*C* and *SI Appendix*, Fig. S9).

Dye-induced phototoxicity seems to be a general limiting factor of live-cell STED imaging of mitochondria since the fluorophores enriched in crista membranes tend to leak into the cytoplasm, implying a drop of MMP when the cells are exposed to high light intensities (32). Indeed, COS-7 cells stained with PKMO 0.9 showed drastic mitochondrial swelling, accompanied by diffusion of the fluorescence signal after very few recorded frames (Fig. 2*D*). In contrast, the COT-conjugated counterpart, PKMO, enabled time-lapse STED recordings of cristae for 30 to 50 frames (Fig. 2*D* and *SI Appendix*, Fig. S10) before the onset of prohibitive photobleaching. To further test the reduced phototoxicity of PKMO, we analyzed the MMP during STED imaging. To this end, we counterstained the cells using Rhodamine 123 (Rho123), a green-fluorescent real-time MMP indicator (33), and recorded it in the confocal mode. Rho123 signal was completely lost after only three STED frames in PKMO 0.9-stained HeLa-cells, whereas PKMO-treated cells experienced a gradual loss of the Rho123 signal and maintained the mitochondrial morphology more than three times longer (*SI Appendix*, Figs. S11 and S12). Next, we compared PKMO labeling with cells expressing COX8A-SNAP and labeled by SiR (6). When aiming at comparable imaging results, photobleaching of SiR was nearly three times stronger than that of PKMO, thereby restricting the practical recording window to ~7 frames. In comparison, PKMO showed strongly reduced photobleaching, allowing the acquisition of >20 frames (*SI Appendix*, Fig. S13 *A–D*). For moderately stained cells, the influence of PKMO on MMP (measured by Rho123 fluorescence) was similar to that of SiR-labeled cells (*SI Appendix*, Figs. S13 *A* and *C* and S14). However, we noticed that in cells that showed very bright PKMO labeling, mitochondria occasionally started to depolarize after ~10 frames (*SI Appendix*, Figs. S13 *B* and *E* and S14). Hence, we recommend optimizing labeling conditions for different cell lines if time-lapse imaging is performed. Altogether, reduced phototoxicity and photobleaching of PKMO allow the recording of more useful super-resolved frames than previously demonstrated chemigenetic approaches.

**Fig. 3.**
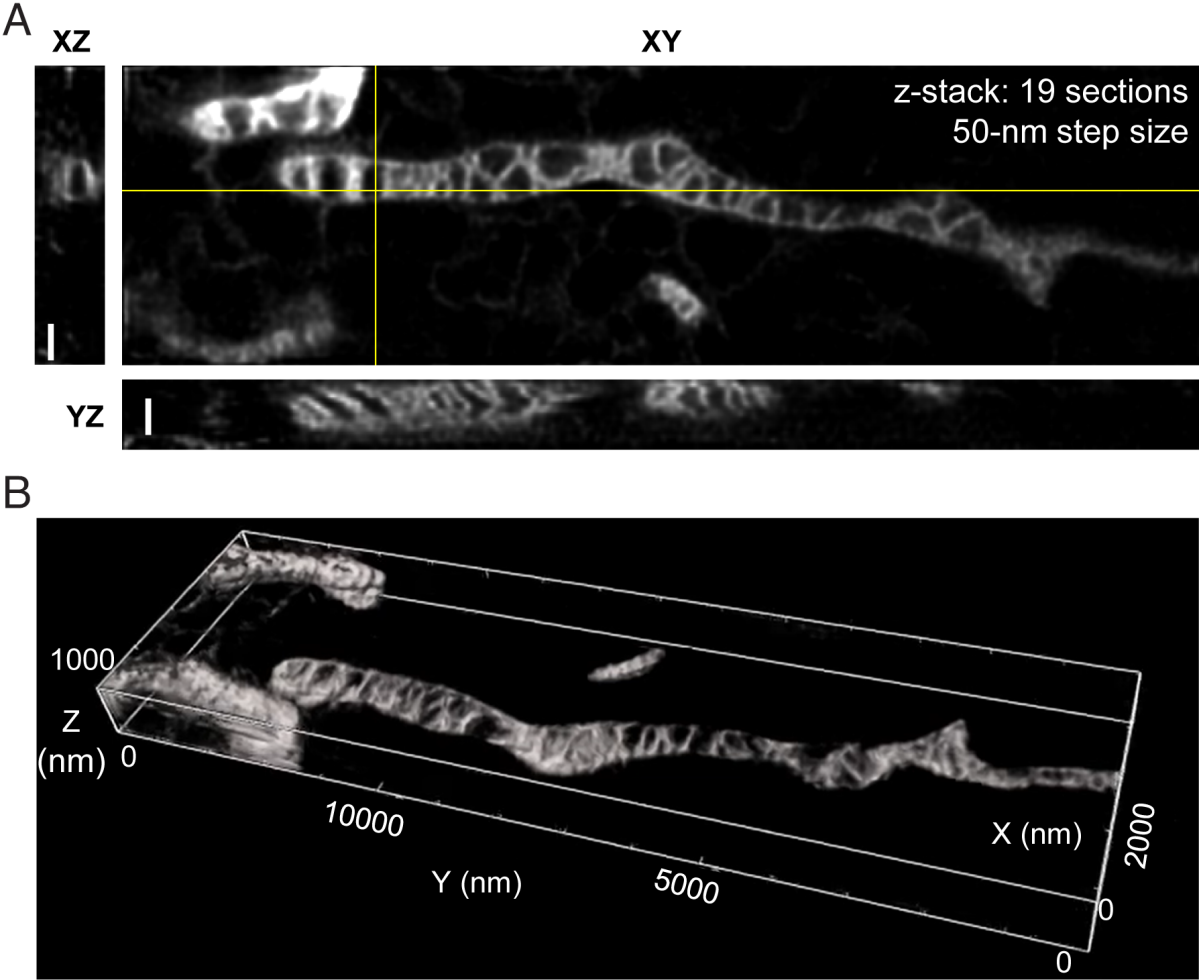
3D-STED imaging and reconstruction of cristae in COS-7 cells labeled with PKMO. *A* and *B*. 3D live-cell STED recording of a mitochondrion from a COS-7 cell labeled with PKMO. *A*. Orthogonal cross-sections through 3D STED recording. (Scale bars, 500 nm.) *B*. 3D reconstruction/volume rendering of 3D STED data (Imaris).

PKMO enabled time-lapse STED recordings of mitochondria over the course of several minutes. This allowed us to follow the mitochondrial IM during fission and tubulation of mitochondria, dynamic processes which are hardly accessible by electron microscopy (Fig. 2 *E* and *F* and *SI Appendix*, Fig. S15 and Movie S1). The excellent performance of PKMO in 2D STED encouraged us to test 3D STED nanoscopy of COS-7 cells as well. Live-cell 3D STED nanoscopy is challenging due to several different limitations such as photobleaching of probes or rapid mobility of mitochondria. Most importantly, phototoxicity effects accumulate by repeated scanning during Z-stacking, causing continuous swelling artifacts. Nevertheless, the gentle nature of PKMO allowed us to record the 3D crista architecture in mitochondria of a live COS-7 cell, at sub100-nm resolution using a 3D STED PSF (Fig. 3 *A* and *B* and *SI Appendix*, Fig. S16 and Movie S2). Orthogonal cross-sections of the recorded volume revealed densely stacked and strongly tilted cristae in the YZ section and hollow sections of mitochondria in the XZ section. Thereby, the data allow 3D mapping of cristae in live mitochondria, demonstrating that 3D STED can resolve complicated crista arrangements, which would be barely visible using 2D nanoscopy.

### PKMO Is a Versatile Crista Marker in Various Cell Lines, Primary Cells, and Organoids.

**Fig. 4.**
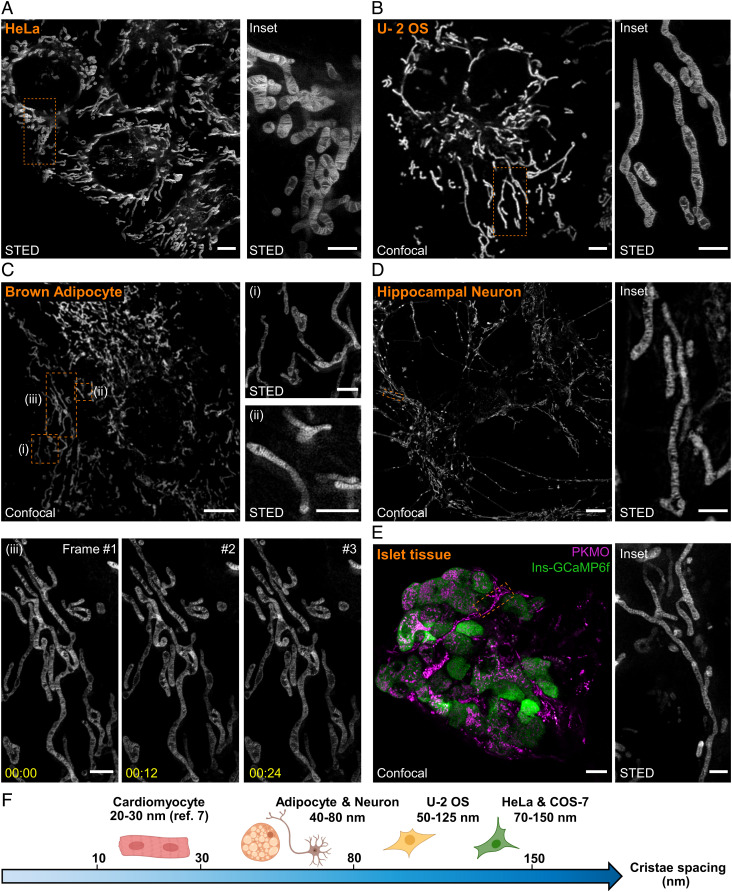
PKMO as a general mitochondrial crista probe enables STED recordings on different cell lines, primary cells, and tissue. *A*. STED overview image (*Left*) of live HeLa cells labeled with PKMO and magnified view (*Right*) of the orange boxed area. (Scale bar, overview 5 μm, *Inset* 2 μm.) *B*. Confocal image of mitochondria labeled with PKMO in U-2 OS cell (*Left*) and magnified view of the dashed boxes in STED mode (*Right*). (Scale bar, overview 5 μm, *Inset* 2 μm.) *C*. STED images of mitochondria labeled with PKMO in primary brown adipocytes (pBAcs). Confocal image of the mitochondria of pBAc (*Left*) and magnified views or time-lapse recordings of the corresponding dashed boxes (i, ii, and iii) in the overview (*Right* and *lower panel*). (Scale bar, overview 5 μm, *Insets* 1 μm.) *D*. Confocal image of mitochondria labeled with PKMO in primary hippocampal neurons (*Left*) and magnified STED image of the dendrite in the boxed region (*Right*). (Scale bar, overview 5 μm, *Inset* 2 μm.) *E*. Dual-color confocal image (*Left*) of mitochondria (magenta, PKMO) and beta cells (green, Ins-GCaMP6f) in the primary islet tissue and magnified STED images (*Right*) of the corresponding orange boxed region (i and ii). (Scale bar, overview 5 μm, *Inset* 1 μm.) *F*. Summary of crista spacing in primary cells and cultivated cancer cells. All STED data were deconvoluted and image series were corrected for photobleaching.

In contrast to labeling strategies based on fluorescent proteins or self-labeling tags, PKMO spontaneously accumulates inside the IM of mitochondria, which should allow rapid and simple labeling of mitochondria in various cell types and tissues. We first evaluated the performance of PKMO across other immortalized cell lines, namely HeLa and U-2 OS cells. As expected, both cell lines showed excellent PKMO labeling and good STED performance (Fig. 4 *A* and *B*), which allowed us to compare the overall shape of the cristae and measure the crista distances in these cells. Crista spacing in HeLa (127 ± 33 nm, *SI Appendix*, Fig. S17) and U-2 OS cells (89 ± 22 nm, *SI Appendix*, Fig. S18) was in a similar size regime compared to that of COS-7 cells (90 ± 24 nm, *SI Appendix*, Fig. S9). However, STED recordings showed that in mitochondria of U-2 OS cells (Fig. 4*B*), the crista arrangements appeared to be less regular compared to the lamellar cristae in COS-7 and HeLa cells (Figs. 2*A* and 4*A*).

So far, live-cell SR microscopy of mitochondria has only been demonstrated in cultivated cancer cells (6, 11, 12, 14, 16, 17, 25, 32, 34–36), which generally feature relatively thick mitochondria and well-ordered cristae. We next tested PKMO for SR imaging of primary cells and tissues (Fig. 4 *C*–*E*). In primary murine brown adipocytes (pBAcs), PKMO delivered bright fluorescence labeling and STED nanoscopy recordings revealed densely spaced (63 ± 14 nm) lamellar cristae (*SI Appendix*, Fig. S19 and Table S1). Remarkably, we were able to follow the dynamic behavior of cristae in pBAcs using time-lapse STED nanoscopy, making PKMO a promising tool for investigating crista dynamics in primary cells (Fig. 4*C* and *SI Appendix*, Figs. S19 and S20). We further tested mitochondrial labeling and crista imaging in live mouse hippocampal neurons (Fig. 4*D* and *SI Appendix*, Fig. S21). PKMO effectively labeled mitochondria throughout the neurons´ cell bodies, axons, and dendrites (Fig. 4 *D*, *Left*), and STED imaging revealed a dense spacing of the cristae (62 ± 20 nm, *SI Appendix*, Fig. S22 and Table S1). Similarly, PKMO could label the fibrillar mitochondrial networks in rat cardiomyocytes (CMs) (*SI Appendix*, Fig. S23). However, STED nanoscopy was not able to visualize the cristae along the mitochondria of these cells. We attribute this to the intrinsic dense packing of cristae in CMs (~20 to 30 nm spacing according to electron microscopy (7) which is beyond the practical resolution of current commercial STED machines). Importantly, PKMO worked well also on isolated mouse islet tissue, which allowed us to resolve the mitochondrial cristae of various cells across the fragile islet tissue (Fig. 4*E*).

Together, PKMO is a convenient mitochondrial probe, which allows quantitative assessment of the crista structure in cancer cell lines, but also in fragile primary cells and tissues (Fig. 4*F*). Often, PKMO delivers similar information as transmission electron microscopy analysis (*SI Appendix*, Fig. S24 and Table S1) but circumvents time-consuming protocols (13) and the potential distortion of the sample during chemical fixation (37). We note, however, that live-cell SR imaging is generally more challenging for primary cells than that for cancer cells. First, many primary cells are often 3D and small in size and have thinner, more elongated mitochondria, which complicates imaging. Second, labeling conditions need to be precisely adapted for different cell types. Third, primary cells are inherently fragile and tended to be more susceptible to photodamage compared to cancer cells. Especially in neurons, mitochondria rapidly developed signs of significant and irreversible damage (*SI Appendix*, Fig. S21), which inevitably compressed the dynamic information. These challenges, in turn, underscore the importance of biocompatibility in the development of probes for nanoscopy (19–21).

### PKMO Is Compatible with Various Live-Cell Dyes and Self-Labeling Tags for Multiplexed Recording.

**Fig. 5.**
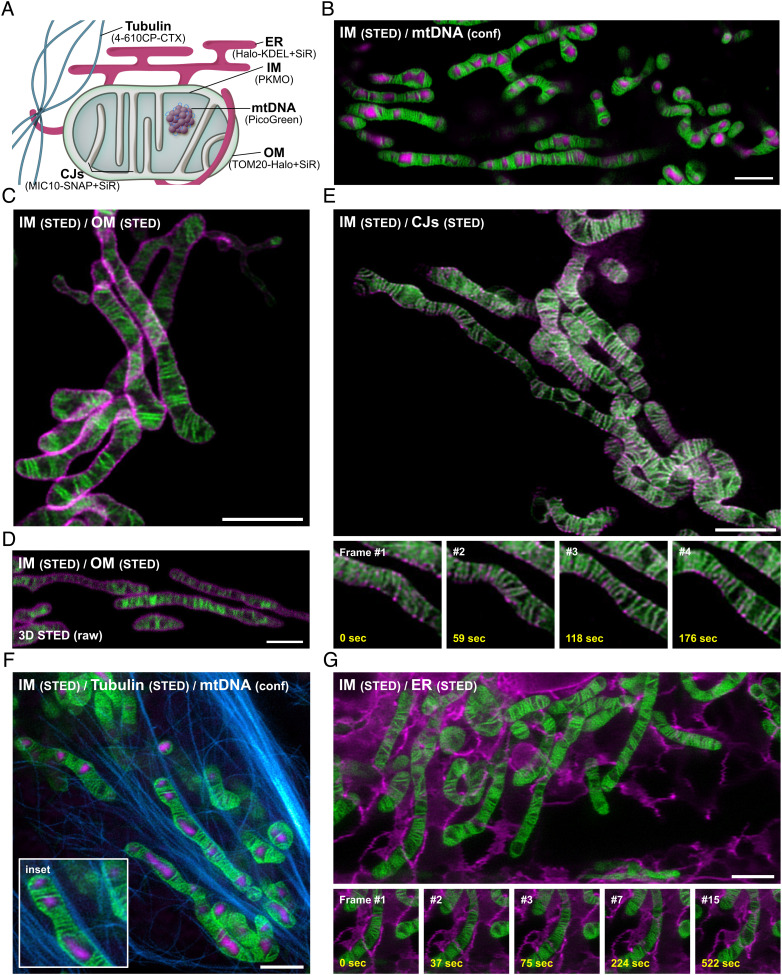
PKMO in conjunction with fluorogenic rhodamine probes enables nanoscopic mapping of mitochondria and the analysis of mito-organelle interactions using multi-color STED nanoscopy. *A*. Labeling strategies for multi-color live-cell recordings of mitochondria. Cartoon demonstrating the labeling strategies for mitochondrial subcompartments and for mitochondria-interacting cellular structures. Abbreviations: ER (endoplasmic reticulum); IM (inner mitochondrial membrane); OM (outer mitochondrial membrane); CJs (crista junctions), mtDNA (mitochondrial DNA). *B*–*G*. Multi-color live-cell STED nanoscopy of HeLa and COS-7 cells labeled for the IM together with different mitochondrial targets or subcellular structures. IM was labeled using PKMO and recorded by live-cell STED nanoscopy (λ_ex_ = 561 nm, λ_STED_ = 775 nm). *B.* 2D single-color STED nanoscopy of mitochondria labeled for mtDNA. The mtDNA was stained using PicoGreen and was recorded in the confocal mode (λ_ex_ = 488 nm). *C* and *D*. Dual-color STED nanoscopy of mitochondria in COS-7 cells. OM marker TOM20-Halo was labeled using 647-SiR-CA (λ_ex_ = 640 nm, λ_STED_ = 775 nm). Cells were recorded by 2D STED in (*C*) or 3D STED nanoscopy (*D*). *E*. 2D dual-color time-lapse STED nanoscopy of mitochondria in HeLa cells. CJs were labeled by overexpression of MIC10-SNAP and staining with SNAP-Cell 647-SiR. *Insets* (*Lower panel*) shows four consecutive frames illustrating cristae and CJ dynamics. *F*. 2D dual-color STED nanoscopy of mitochondria and microtubules in HeLa cells. Microtubules were labeled using 4-610CP-CTX (λ_ex_ = 640 nm, λ_STED_ = 775 nm). MtDNA was labeled using PicoGreen. *Inset* highlights contact sites of mitochondria and microtubules. *G*. 2D dual-color time-lapse STED nanoscopy of mitochondria and ER in HeLa cells. ER was labeled by overexpression of Halo-KDEL and staining with 647-SiR-CA. *Insets* (*Lower panel*) highlights contact sites of a mitochondrion and ER over several time points. If not indicated otherwise, all image data were deconvoluted and corrected for photobleaching. (Scale bars, 2 µm.)

The double-membrane architecture of mitochondria creates mitochondrial subcompartments, which serve different purposes (Fig. 5*A*). Traditionally, biochemical analysis or electron microscopy has been used to analyze the submitochondrial localization of proteins, but multicolor nanoscopy would open the possibility to target protein localization in live cells. PKMO is designed for multicolor nanoscopy with its peak emission in the orange part of the spectrum. Excitation at 561 nm wavelength and depletion at 775 nm allow to combine PKMO with green fluorophores, which can be recorded in the confocal mode, or with a wide palette of deep-red fluorophores for dual-color STED imaging. We tested the multi-color performance of PKMO in HeLa and COS-7 cells labeled with different transfection-free fluorescent probes or by expressing fusion constructs with the self-labeling SNAP-tag or HaloTag (for an overview of labeling strategies, see Fig. 5*A*).

#### PKMO allows analyzing submitochondrial compartments.

The mtDNA is packed into nucleoids, which are located within the matrix of the organelle. In order to localize the mtDNA across the mitochondrial network of HeLa cells, we stained the cells with PKMO and the dsDNA-binding green-emitting probe PicoGreen. This allowed us to capture the spatial organization of the mtDNA (λ_ex_ 488nm, confocal) alongside the cristae (λ_ex_ 561 nm, λ_STED_ 775 nm) over the time course of several minutes (Fig. 5*B* and *SI Appendix*, Fig. S25 and Movie S3). During this time, we observed significant remodeling of individual cristae and the overall mitochondrial network, but we found that the mtDNA remained trapped within the larger voids between groups of densely stacked cristae, suggesting that lamellar cristae generally act as diffusion barriers, which can separate different nucleoids (6).

Next, we aimed at a nanoscopic differentiation of the mitochondrial outer membrane (OM), IM, and the CJs using dual-color live-cell STED nanoscopy. To this end, we expressed the outer mitochondrial membrane (OM) protein TOM20 and the CJ marker MIC10 fused to the HaloTag and SNAP-tag, respectively. We stained the cells with PKMO and the deep-red fluorophores 647-SiR-CA or SNAP-Cell 647-SiR (15). Both PKMO (λ_ex_ 561 nm) and SiR (λ_ex_ 640 nm) are efficiently depleted using the 775-nm STED wavelength implemented in most STED microscopes. As expected, we were able to resolve the mitochondrial OM surrounding the PKMO-enriched IM using 2D STED as well as using 3D STED (Fig. 5 *C* and *D* and *SI Appendix*, Figs. S26 and S27). Similarly, we were able to highlight individual CJs alongside the cristae and capture their dynamic movements over a few frames (Fig. 5*E* and *SI Appendix*, Fig. S26 *A* and *C*). Our data demonstrate that by combining PKMO with self-labeling tags, multi-color live-cell STED can reveal the different submitochondrial localizations of biomolecules. Thereby, STED imaging can deliver similar information as the technically more demanding immunogold transmission electron microscopy (38). Importantly, our approach does not require extensive sample preparation and allows drawing additional information about the dynamics of the involved structures and biomolecules. However, we note that rapid photobleaching of SiR (*SI Appendix*, Figs. S26) could compromise the overall number of recordable frames compared to single-color recordings.

#### PKMO allows analyzing mito-organelle interactions.

Mitochondria form a highly dynamic and interconnected tubular network that pervades the entire cytosol and features contact sites with different cellular structures. Especially important are interactions of mitochondria with the ER (39). Contact sites mediate the transport of lipids between both organelles (40, 41) and control the overall fusion–fission dynamics of the mitochondrial network (42–45). Similarly, interactions with the tubulin cytoskeleton are pivotal for cellular function as they enable proper mitochondrial transport (46). An important aspect of future live-cell SR microscopy studies will be the analysis of such interactions between mitochondria and other cellular structures at high spatiotemporal resolution. To test the performance of PKMO in such scenarios, we labeled HeLa cells for the tubulin cytoskeleton using the biocompatible fluorescent probe 4-610-CP-CTX (47) or for the endoplasmic reticulum (ER) by overexpressing Halo-KDEL (stained with 647-SiR-CA). Cells were colabeled with PKMO and analyzed by live-cell dual-color STED nanoscopy. This approach allowed us to capture the spatial arrangement of mitochondria and the cytoskeleton at high spatial resolution (Fig. 5*F*) and to follow the interaction of the mitochondrial tubules with the ER over the time course of several minutes (Fig. 5*G* and *SI Appendix*, Fig. S28 and Movie S4).

#### PKMO allows following crista remodeling upon cellular stimuli.

**Fig. 6.**
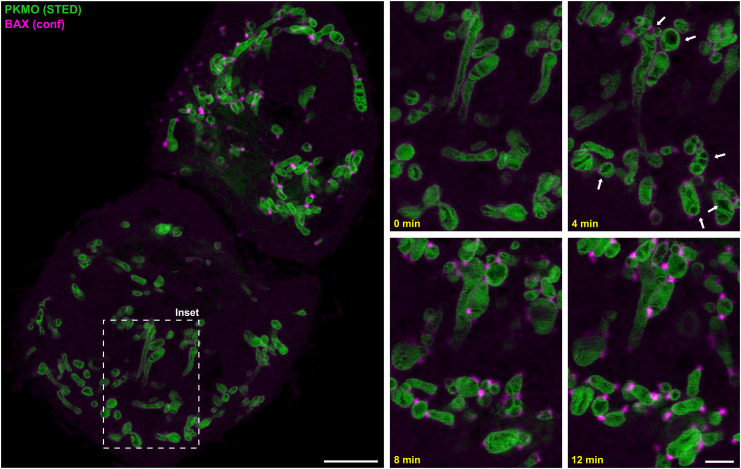
Live-cell STED nanoscopy of apoptotic cells. HeLa cells labeled with PKMO (250 nM, 45 min) were transfected with mEGFP-BAX to induce apoptosis. Cells were recorded 4 to 6 h after transfection. PKMO was recorded using STED, and mEGFP-BAX was recorded in the confocal mode. *Left*: Overview recording, showing two cells expressing different amounts of mEGFP-BAX (magenta). *Right*: Time-lapse recording of the area indicated by the dashed box. Shown are four consecutive frames. Arrows indicate mitochondria that undergo severe changes of the crista morphology, once mEGFP-BAX clusters appeared along the mitochondria. Scale bars, 5 µm (overview), 1.5 µm (*Inset*).

Apoptosis involves permeabilization of the mitochondrial OM and the release of a cocktail of proteins into the cytosol. This process also involves remodeling (48–50) and ultimately permeabilization of the IM (51), but little is known about crista dynamics during this process. We stained HeLa cells with PKMO and transfected them to overexpress the proapoptotic Bcl-2-associated X protein (BAX) fused with monomeric enhanced green fluorescent protein (mEGFP) to induce apoptosis (52). Four to six hours after transfection, mEGFP-BAX translocated to the mitochondria and started to form distinct clusters, marking the formation of proapoptotic pores (53). Live-cell STED with PKMO allowed us to follow the cristae during this process and revealed drastic IM remodeling, including mitochondrial swelling, once the first BAX clusters appeared (Fig. 6).

##### PKMO Paves the Way for a Nanoscopic Screening of the Crista Architecture in Living Cells.

**Fig. 7.**
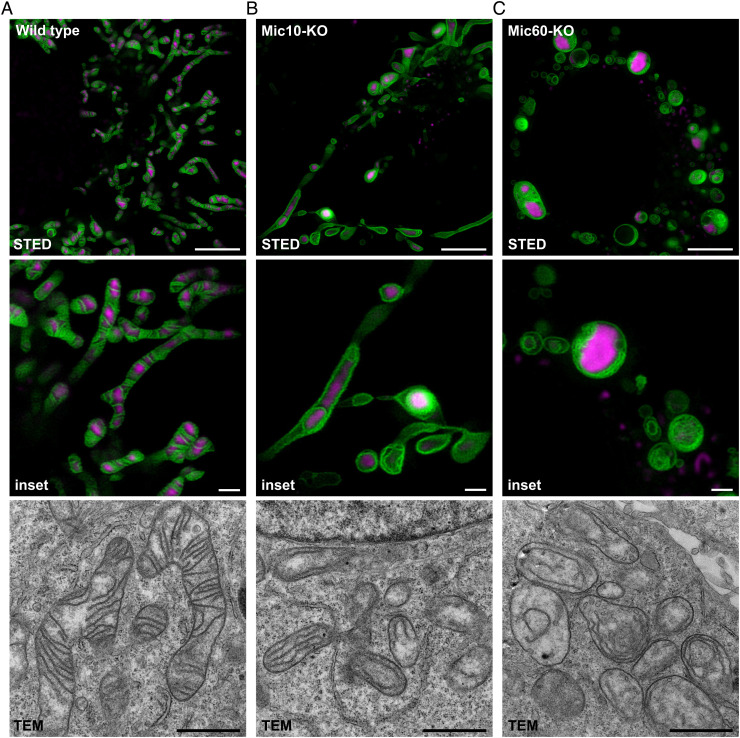
Analysis of crista architecture using PKMO labeling and 2D live-cell STED nanoscopy. *A*–*C*. The mitochondrial inner membrane (IM) of HeLa cells was labeled using PKMO (λ_ex_ = 561 nm, λ_STED_ = 775 nm). MtDNA was labeled using PicoGreen (λ_ex_ = 488 nm, confocal). *A*. STED (*Upper*) and transmission electron microscopy (TEM) recording (*Lower*) of wild-type HeLa cells with typical lamellar crista architecture. *B*. STED (*Upper*) and TEM recording (*Lower*) of genome-edited HeLa cells lacking MIC10, a subunit of the mitochondrial contact site and crista organizing system (MICOS complex). STED nanoscopy reveals tube-like and onion-like crista arrangements and a disturbed arrangement of the mtDNA. *C*. STED (*Upper*) and TEM recording (*Lower*) of genome-edited HeLa cells lacking MIC60, the core subunit of MICOS. STED nanoscopy reveals a fragmented mitochondrial network, onion-like crista arrangements, and aggregations of mtDNA. All STED data were deconvoluted. Scale bars, STED (overview) 5 µm, STED (*Insets*) 1 µm, TEM 1 µm.

The unique structure of the mitochondrial IM is inextricably linked to the functionality of mitochondria as the powerhouses of the cells. Defects of the crista architecture are related to malfunction of cellular respiration and are associated with neurodegenerative or cardiac diseases (54). Mutations or complete loss of proteins that control crista morphology cause drastic remodeling of the crista architecture (55–58). Typically, such changes of the crista architecture have been analyzed using transmission electron microscopy of ultrathin sections of fixed cells. PKMO opens the door to perform such analysis also in living cells. As an example, we show cells genetically modified by CRISPR-Cas9 to silence the expression of MIC10 or MIC60, core subunits of the mitochondrial contact site, and the crista organizing system (MICOS) that controls CJ formation. We labeled these cells with PKMO and PicoGreen and recorded them using 2D live-cell STED. STED recordings showed that all cell lines feature distinct crista phenotypes (Fig. 7 *A–C*). Whereas HeLa wild-type cells showed highly ordered lamellar cristae (Fig. 7*A*), the cristae appeared as single- or double-layered tubes that lined the mitochondrial interior in the absence of MIC10 (Fig. 7*B*). This phenotype is caused by a reduction in the number of CJs and by a defective segmentation of the IM into individual cristae (17). MIC60-depleted HeLa cells showed a different phenotype (Fig. 7*C*). The mitochondrial network of these cells was largely fragmented, and the large spherical mitochondria accumulated multiple layers of cristae, since MIC60 depletion causes virtually a complete disruption of CJs (16, 17, 57, 59). Transmission EM recordings of the same cell lines (Fig. 7 *A–C*) demonstrated that live-cell STED delivers similar information regarding the overall crista arrangements as the electron microscopy recordings of resin-embedded samples. In addition, multi-color recordings allowed drawing additional information that are barely extractable from EM recordings. Our recordings showed that MICOS-depleted mitochondria did not only feature disturbed cristae but also featured an aberrant distribution of the mtDNA, which accumulated into large aggregates (Fig. 7 *B* and *C*).

## Discussion and Conclusion

In this study, we introduce PKMO, a gentle and versatile mitochondrial inner membrane stain that has been tailored for multi-color STED imaging. From a perspective of fluorophore chemistry (60), this work brings the classic cyanine fluorophores to the stage of live-cell nanoscopic imaging. Despite the widespread use of cyanine dyes in advanced fluorescent microscopies such as single-molecule localization microscopy (SMLM) (61), single-molecule fluorescence resonance energy transfer microscopy (SM-FRET) (22), nanoscopy with minimal photon fluxes (MINFLUX) (62–64), and near infrared in-vivo imaging (65), rhodamine dyes are often preferentially used in STED-based bioimaging (66–68). This work shows that a cyanine dye, once equipped with triplet-state quenchers and spectroscopically tailored, offers superior performance in live-cell STED nanoscopy. Thereby, it adds itself to the ever-growing mitochondrial imaging options (12, 14, 69, 70) and also sheds light on potential development strategies of future live-cell stains for nanoscopy.

Phototoxicity has long been recognized as an important parameter and is considered as a main hurdle of live-cell STED microscopy (19), but approaches to chemically alleviate it were falling disproportionally short. From the perspective of probe development, the conceptual advance of this work lies in the introduction of triplet-state depleted dyes (22, 24) into STED nanoscopy to minimize photodamage. This work demonstrates that a dedicated mitochondrial stain can be gentle enough to allow the recording of ~20 z-planes in a live cell for reconstructing cristae in 3D, opening promising possibilities for the analysis of mitochondria in living cells. Using PKMO, we demonstrate that live-cell SR microscopy of mitochondria can be extended to fragile primary cells such as beta cells or neurons, thus enabling the investigation of disease-related processes at the precision of nanoscopy.

Currently, numerous questions on the mechanisms that govern the dynamics of the mitochondrial IM are unsolved. The networks of mitochondrial tubules are highly dynamic, exhibiting frequent fission and fusion events. Thereby, the fission/fusion of IM needs to be coordinated with that of the OM. Intriguingly, it was recently reported that mitochondrial fission sites are determined either by ER contact sites or lysosomal contact, leading to the biogenesis of mitochondria or clearance of defective mitochondria, respectively (45, 71). However, it remains unclear whether the IM behaves differently at the respective sites. Likewise, the molecular mechanisms that govern crista biogenesis and regulate the adaptation of crista shapes to different metabolic conditions are still poorly understood (3). We assume that PKMO will aid studies into the underlying molecular mechanisms that govern mitochondrial inner membrane dynamics. An important advancement reported in this study is the introduction of reliable multi-color live-cell nanoscopy of mitochondria. Our dual-color recordings support previous findings that nucleoids are primarily localized between groups of cristae (6), challenging the view that the mtDNA is consistently positioned in close proximity to the IM. In conjunction with promising STED dyes that label mtDNA (72), multi-color STED nanoscopy will be able to follow individual nucleoids and investigate, for example, the influence of nucleoid heterogeneity (73) on the IM structure.

Together, PKMO facilitates the investigation of crista dynamics, organelle interactions, and mitochondrial morphologies in cells and tissues in a multiplexed manner at sub-100 nm resolution and for longer durations than previously possible.

## Materials and Methods

For a detailed description of the experimental procedures including chemical synthesis of PKMO, spectroscopy, bulk bleaching assays, singlet oxygen quantum yield measurements, cell culture, cell assays, primary cell and tissue isolation and culture, plasmids, transfection, phototoxicity assay, electron microscopy, PKMO labeling, image processing, and analysis (*SI Appendix*).

### Labeling for Live-cell Imaging of Cancer Cells, Primary Cells, and Tissue.

The progression and intensity of PKMO labeling are dependent on the individual cell line and the culture condition. For optimal results, we recommend optimizing PKMO concentration and staining duration for each cell line. For initial testing, we recommend staining cells using 250 nM PKMO for 15 to 20 min at 37 °C and 5% CO_2_. If necessary, staining might be extended for up to 1 h at staining concentrations of up to 600 nM PKMO. For a summary of PKMO concentration and labeling duration (*SI Appendix*, Tables S2 and S3).

#### PKMO labeling of cancer cells.

COS-7 cells were stained with DMEM supplemented with 250 nM PKMO for 15 min at 37 °C. U-2 OS cells were stained with McCoy's medium supplemented with 250 nM PKMO for 20 min at 37 °C. For single-color recordings, HeLa cells were stained with DMEM containing 150 to 250 nM PKMO for 40 to 45 min at 37 °C. Following the staining procedure, the cells were washed three times with culture medium and incubated for 30 to 60 min to remove the unbound dye. The cells were imaged in Dulbecco's Modified Eagle Medium (DMEM) buffered by 4-(2-hydroxyethyl)-1-piperazineethanesulfonic acid (HEPES) at room temperature (*SI Appendix*).

#### PKMO labeling of primary cells.

pBAcs and neurons were stained with DMEM containing 250 nM PKMO at 37 °C for 15 min. CMs were stained with 500 nM PKMO in DMEM for 15 min at 37 °C before imaging experiments. Islet tissues were stained with Krebs–Ringer bicarbonate buffer solution containing 600 nM PKMO at 37 °C for 30 min. After removing the staining solution, the cells/tissues were washed with medium once and maintained in fresh medium for subsequent STED imaging as described in *SI Appendix*.

#### Sample preparation for multi-color imaging.

For labeling of crista junctions, outer membrane, and ER, cells were transfected for overexpression of MIC10-SNAP, TOM20-Halo, or Halo-KDEL, respectively. The cells were stained with 150 to 350 nM PKMO together with 1 µM SNAP-Cell 647-SiR or 0.5 µM 647-SiR-CA as described in detail in *SI Appendix*. For labeling of cristae and mtDNA, HeLa cells were incubated with DMEM containing 150 nM PKMO and 0.5 μL/mL PicoGreen reagent as described in *SI Appendix*. Labeling of cristae together with the cytoskeleton and mtDNA was achieved using 350 nM PKMO, 200 nM 4-610CP-CTX, and 0.2 μL/mL PicoGreen reagent as described in *SI Appendix*. After staining, the culture medium was replaced, and the cells were incubated in the culture medium for 30 to 60 min to remove unbound dye. For analysis of apoptosis, the cells were stained with 250 nM PKMO. Afterward, apoptosis was induced by the expression of mEGFP-BAX as described in *SI Appendix*. The cells were recorded in HEPES-buffered DMEM at room temperature. Further details may be found in the *SI Appendix*.

### Live-Cell Imaging of Cancer Cells, Primary Cells, and Tissue.

COS-7, HeLa, U-2 OS cells, primary cells, and tissues were recorded using a Facility Line microscope (Abberior Instruments) equipped with an Olympus UPlanXAPO 60× oil/NA1.42 objective, a STEDYCON STED microscope (Abberior Instruments) equipped with a CFI Plan Apochromat Lambda D 100× oil/NA1.45 objective, or an Expert Line dual-color STED 775 QUAD scanning microscope (Abberior Instruments) equipped with a UPlanSApo 100×/1.40 Oil [infinity]/0.17/FN26.5 objective. PKMO was excited at 561 nm wavelength, and STED was performed using a pulsed depletion laser at 775 nm wavelength with gating of 1 to 7 ns and dwell times of 5 to 10 μs. Pixel sizes of 20 to 30 nm were used for STED nanoscopy and each line was scanned 3 to 10 times (line accumulations). The pinhole was set to 0.7 to 1.0 AU. For dual-color STED imaging, PKMO was excited at 561 nm and SiR was excited at 640 nm wavelength. Depletion was performed at a 775-nm wavelength. PicoGreen, mEGFP, and Rho123 were excited at 485 nm wavelength and were recorded in the confocal mode. For a detailed summary of imaging parameters (*SI Appendix*, Table S3).

## Supplementary Material

Appendix 01 (PDF)

Movie S1. Time-lapse STED nanoscopy recording of mitochondrial cristae dynamics in a COS-7 cell labeled with PKMO.

Movie S2. 3D-reconstruction of an individual mitochondrion in a COS-7cell labeled with PKMO and recorded using 3D live-cell STED nanoscopy.

Movie S3. Time-lapse STED nanoscopy recording of mitochondrial cristae (STED, green) and mtDNA (confocal, magenta) in a HeLa cell labeled with PKMO.

Movie S4. Dual-color time-lapse STED nanoscopy recording of mitochondrial cristae (green) and ER (magenta) in a HeLa cell labeled with PKMO.

## Data Availability

All study reported in this paper will be shared by the corresponding author upon reasonable request. PKMO is commercially available from Genvivo Biotech (Nanjing, China) and Spirochrome AG (Stein am Rhein, Switzerland).
