# Supplementary material for: Multi-color live-cell STED nanoscopy of mitochondria with a gentle inner membrane stain
Source: Proc Natl Acad Sci U S A. 2022 Dec 19;119(52):e2215799119. doi: 10.1073/pnas.2215799119 (PMC9907107; doi:10.1073/pnas.2215799119)
Supplement: Supplementary file 1 — Appendix 01 (PDF) [file pnas.2215799119.sapp.pdf]

PNAS

Supplementary Information for
Multi-color live-cell STED nanoscopy of mitochondria with a gentle inner membrane stain

Tianyan Liu 1,2,10, Till Stephan 3,4,10, Peng Chen 5,6, Jan Keller-Findeisen 3,7, Jingting Chen 1, Dietmar Riedel 8, Zhongtian Yang 2, Stefan Jakobs 3,4,7,9, and Zhixing Chen 1,2,5,6*

1) College of Future Technology, Institute of Molecular Medicine, National Biomedical Imaging Center, Beijing Key Laboratory of Cardiometabolic Molecular Medicine, Peking University, Beijing 100871, China
2) Peking-Tsinghua Center for Life Science, Academy for Advanced Interdisciplinary Studies, Peking University, Beijing 100871, China
3) Department of NanoBiophotonics, Max Planck Institute for Multidisciplinary Sciences, Göttingen 37077, Germany
4) Clinic of Neurology, University Medical Center Göttingen, Göttingen 37075, Germany
5) Peking University-Nanjing Institute of Translational Medicine, Nanjing 211800, China
6) Genvivo Biotech, Nanjing 211800, China
7) Fraunhofer Institute for Translational Medicine and Pharmacology ITMP, Translational Neuroinflammation and Automated Microscopy TNM, Göttingen 37075, Germany
8) Laboratory of Electron Microscopy, Max Planck Institute for Multidisciplinary Sciences, Göttingen 37077, Germany
9) Cluster of Excellence “Multiscale Bioimaging: from Molecular Machines to Networks of Excitable Cells” (MBExC), University of Göttingen, Göttingen 37099, Germany
10) Equal contribution

*Corresponding author: Zhixing Chen (zhixingchen@pku.edu.cn)

This PDF file includes:

Supplementary information text
- Legends for Movies S1 to S4
- Material and Methods
- Figures S1 to S28
- Table S1-S3

Chemical synthesis and characterization of new compounds

SI References

Other supplementary materials for this manuscript include the following:

Movies S1 to S4

Legends for Movies S1 to S4

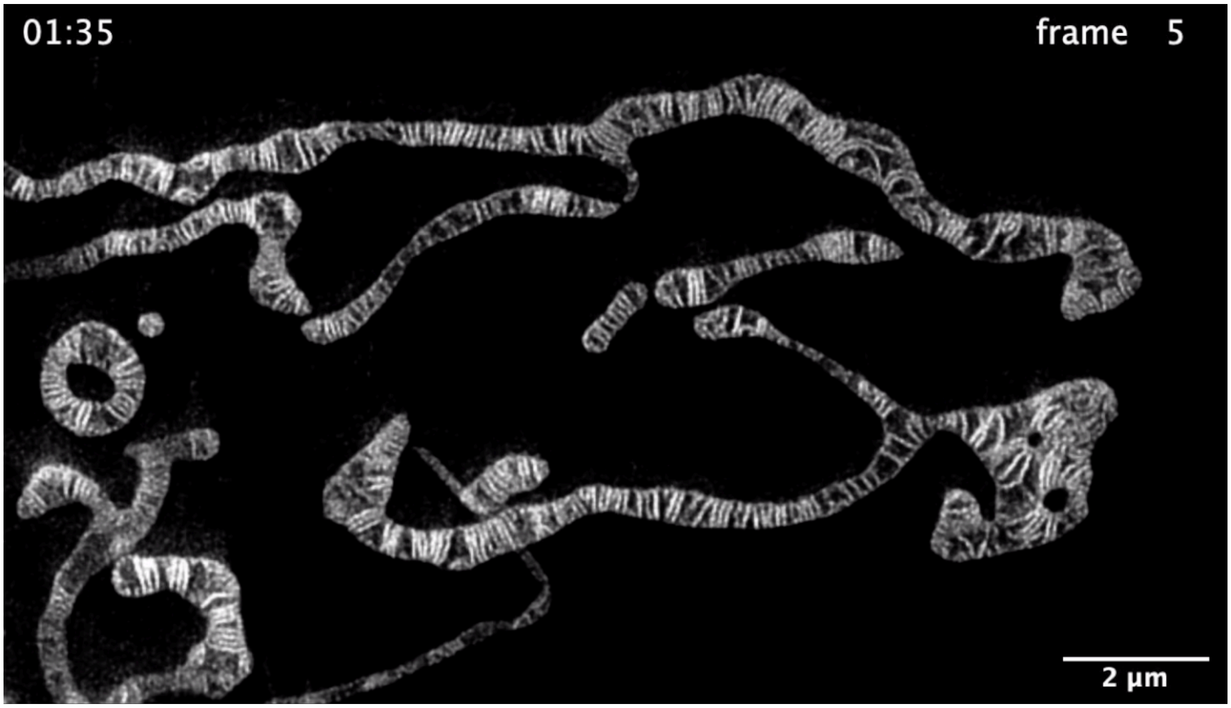

Movie S1. Time-lapse STED nanoscopy recording of mitochondrial cristae dynamics in a COS-7 cell labeled with PKMO.

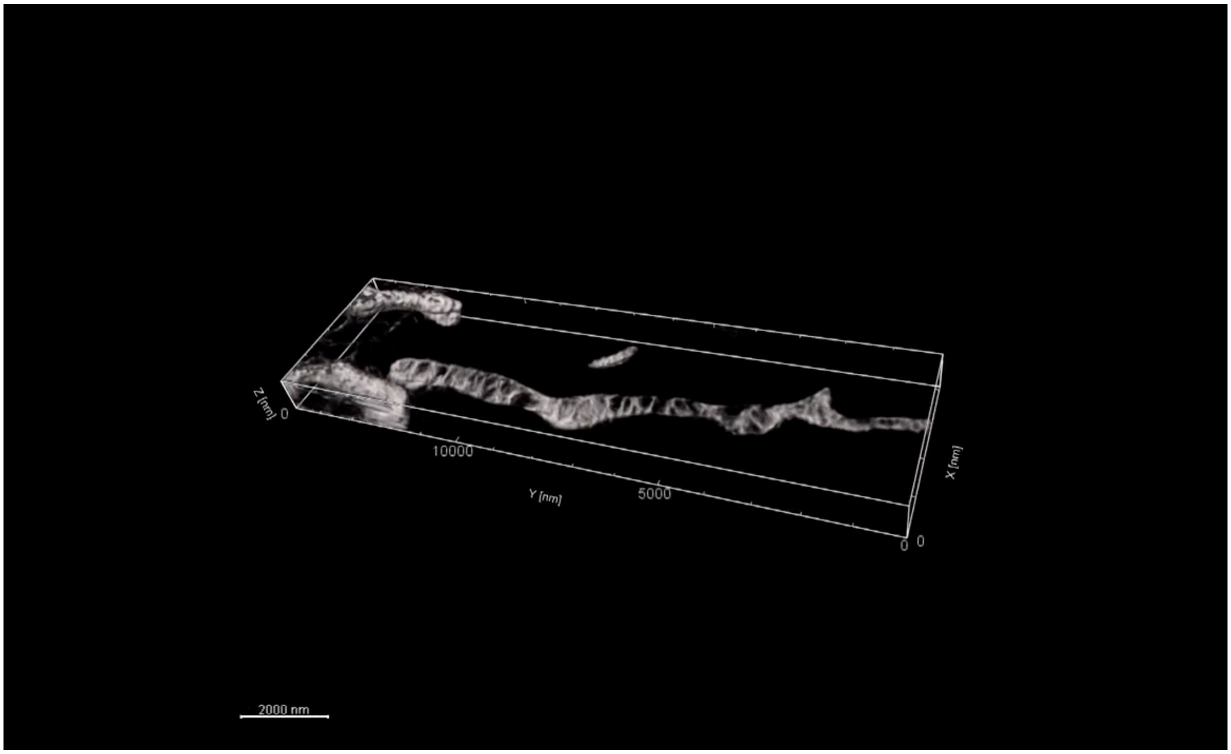

Movie S2. 3D-reconstruction of an individual mitochondrion in a COS-7cell labeled with PKMO and recorded using 3D live-cell STED nanoscopy.

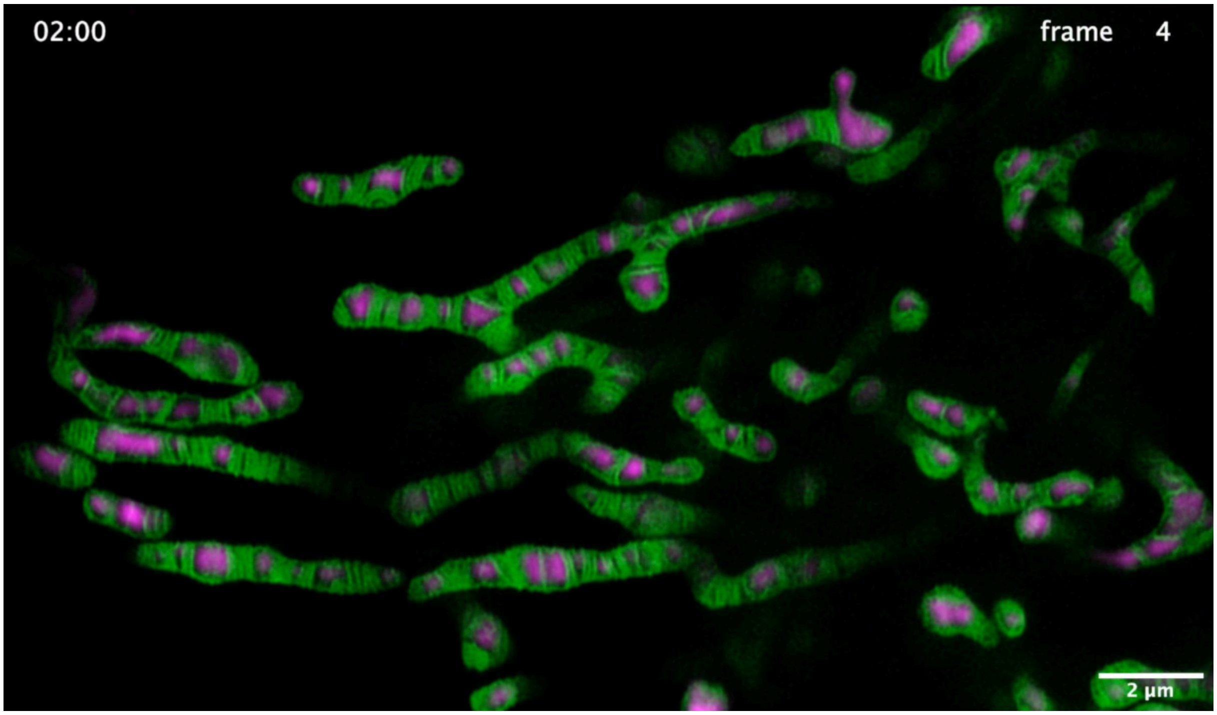

Movie S3. Time-lapse STED nanoscopy recording of mitochondrial cristae (STED, green) and mtDNA (confocal, magenta) in a HeLa cell labeled with PKMO.

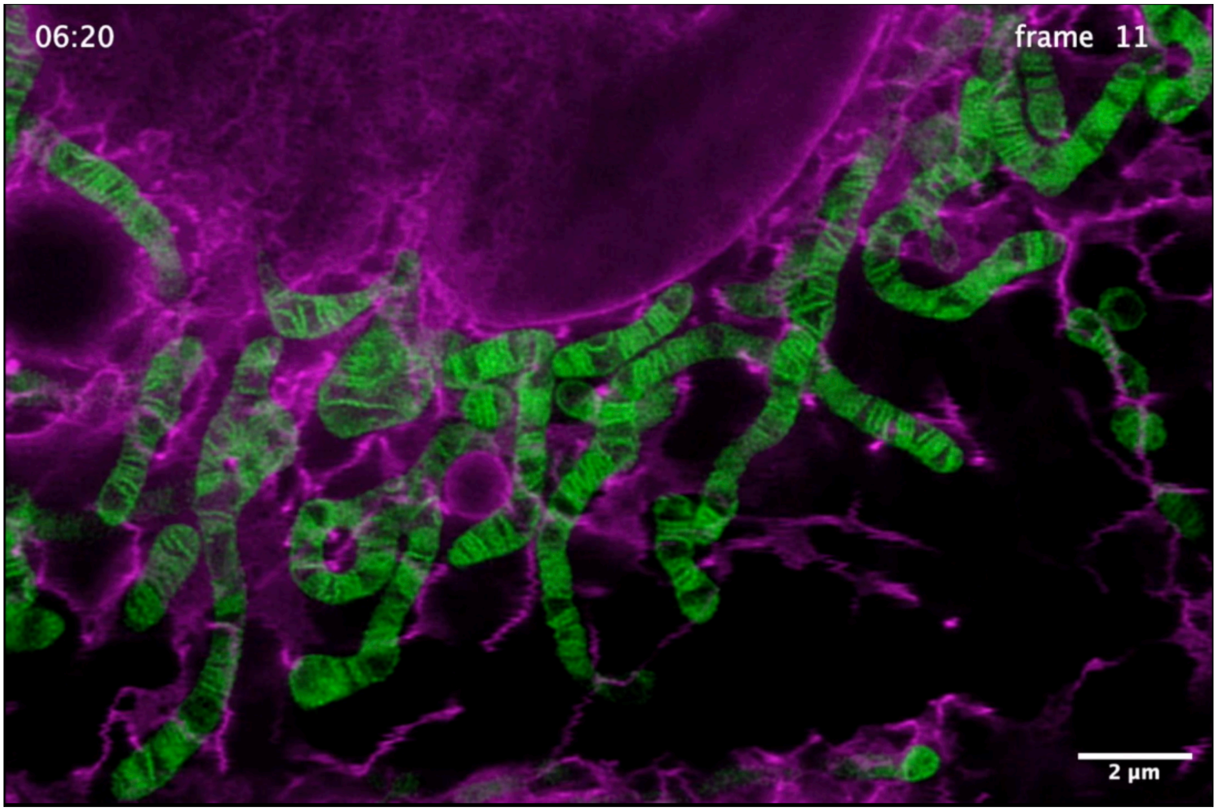

Movie S4. Dual-color time-lapse STED nanoscopy recording of mitochondrial cristae (green) and ER (magenta) in a HeLa cell labeled with PKMO.

Supplementary Information Text

Material and Methods

UV-Vis and fluorescence spectroscopy

Stock solutions of PKMO and PKMO 0.9 were prepared in DMSO solvent and diluted with methanol (MeOH) to 1 μM. UV-vis absorption spectra of sample solutions were measured using a Shimadzu UV3600Plus UV-VIS-NIR Spectrophotometer (Shimadzu, Kyōto, Japan) in a 1cm square quartz cuvette. Fluorescence emission spectra were measured using a Shimadzu RF-5301PC spectrofluorometer (Shimadzu) in a 1cm square quartz cuvette. The concentration of dyes was determined based on the absorbance at 591 nm ($\varepsilon_{MeOH} = 1.05\times10^5$ $M^{-1}cm^{-1}$) (1).

Quantum yield determination

Stock solutions of PKMO and PKMO 0.9 were prepared in DMSO solvent and diluted with methanol or toluene to 1 μM. Absolute quantum yields of dyes were measured using a FLS980 Spectrometer equipped with an integrating sphere (FLS980; Edinburgh Instruments, Livingston, England) at room temperature. Measurements were carried out using diluted samples (A < 0.1) at their excitation wavelength (560 nm).

Bulk bleaching measurement

Dyes were immobilized within polyvinyl alcohol (PVA) and polymethylmethacrylate (PMMA) films prepared using a SW-4A spin coater (Setcas, Beijing, China) and round glass coverslips (diameter = 25 mm) as substrates. For PVA films, 100 μL 5% PVA (S27770-500g; Yuanye, Shanghai, China) in Mili-Q water containing 2 μM dyes were applied to the coverslip followed by spin coating (80,000 rpm, 10 sec; 300,000 rpm, 45 sec); for PMMA films, 100 μL 5% PMMA in chloroform (112048; Tongguang, Beijing, China) containing 2 μM dyes were applied to coverslip followed by spin coating (80,000 rpm 10 sec; 800,000 rpm 45 sec). These polymer films were then irradiated using a ZEISS LSM880 confocal microscope (Carl Zeiss AG, Oberkochen, Germany) equipped with a 20x Air Plan Apochromat 20X/0.8 NA objective. Three groups of time-lapse images (500 frames,10.6 μm x 10.6 μm, 1.6 fps, 100% laser power) were acquired for each dye, and quantification of the fluorescence intensity was achieved via Analyze >> Tools >> ROI manager in the Fiji software from three parallel experiments.

Measurement of singlet oxygen quantum yield

Singlet oxygen quantum yields were determined using 1,3-Diphenylisobenzofuran (DPBF, Q105708; Dibai, Shanghai, China) as a chemical indicator. 10 μM dye and 8 x 10^{-5} M DPBF were dissolved in air-saturated acetonitrile (ACN), followed by irradiation with an LED lamp (520-530 nm, 50 mW/cm^2). For PKMO, PKMO 0.9, and TMRE, the dyes were corrected for concentration so that their absorbance values at 525 nm were equal. DPBF absorption was monitored at 415 nm. The linear decay slope of 415 nm is positively correlated with singlet oxygen quantum yield. TMRE (φ = 0.012) in MeOH was used as a reference (2).

Cell culture and isolation of primary cells and tissues

COS-7 (MB5841; HARVEYBIO, Beijing, China) and HeLa (a gift from Prof. Wulan Deng, Peking University) cells for phototoxicity measurements were cultured in a high-glucose Dulbecco´s modified Eagle´s medium (DMEM) (Gibco, 11965092; Thermo Fisher Scientific, Waltham, MA, USA) containing 10% (v/v) heat-inactivated fetal bovine serum (SE100-011; Vistech, Sydney, Australia) and 1% (v/v) penicillin sulfate and streptomycin (CC004; Macgene, Beijing, China).
For super-resolution imaging, HeLa cells (3) were grown in DMEM with glutaMAX™ additive and 4.5 g/l glucose (Thermo Fisher Scientific, USA). The culture medium was supplemented with 1% (v/v) penicillin-streptomycin (Sigma Aldrich, Munich, Germany), 1 mM sodium pyruvate (Sigma Aldrich), and 10% (v/v) FBS (Merck Millipore, Burlington, MA, USA). U-2 OS cells (European Collection of Authenticated Cell Cultures, ECACC; cat. no. 92022711) were cultured in McCoy´s Medium (Thermo Fisher Scientific) supplemented with 10% (v/v) FBS (Merck Millipore), 1% (v/v) sodium pyruvate (Sigma Aldrich) and 1% penicillin-streptomycin (Sigma Aldrich).

The isolations of primary brown adipocytes, hippocampal neurons, cardiomyocytes, and islet tissues were performed according to (4-7). Primary rat cardiomyocytes (CMs) and primary brown adipocytes (pBAcs) were cultured in DMEM. Islets tissues isolated from Ins1-Cre$^{+/+}$; GCaMP6f$^{fl/fl}$ mice were cultured on glass-bottom dishes in RPMI 1640 supplemented with 10% FBS, 8 mM glucose, and 100 U/mL and 100 mg/ml Pen/Strep. Ins1-Cre$^{+/+}$; GCaMP6f$^{fl/fl}$ mice were crossbred by the Ins1-Cre (Jackson Laboratories, Bar Harbor, ME, USA; stock number 026801) and GCaMP6f$^{fl/fl}$ lines (Jackson Laboratories; stock number 029626) (7). All cells were cultured in an incubator at 37°C with 5% CO_2.

Transfection of cells

For expression of fusion proteins, HeLa cells were transfected 24-48 h prior to imaging using jetPRIME® transfection reagent (Polyplus-transfection SA, Illkirch-Graffenstaden, France). Transfection was carried out according to the manufacturer protocols using 1-2 µg plasmid DNA. COS-7 cells were transfected 48 h prior to imaging using lipofectamine® 3000 reagent (Thermo Fisher Scientific). Transfection was carried out according to the manufacturer's protocols using 2.5 µg plasmid DNA.

Plasmids

CJ-labeling was carried out by transfection with pH-MINOS1-SNAP (8). ER-labeling was performed by transfection with the plasmid Halo-KDEL (9) TOM20-Halo was expressed by transfection with plasmid TOM20-Halo.To generate TOM20-Halo, the plasmid TOM20-eDHFR: L28C (10) was first linearized using the restriction endonucleases EcoRV and XhoI. The gene encoding HaloTag® was amplified by PCR from EF1α-H2B-Halo (a gift from Prof. Wulan Deng) using the oligonucleotides (a) and (b) and subsequently integrated into the linearized plasmid by Gibson assembly®.

(a): 5'-AGATGATATCAAGCTTACCATGGGCAGCGAAATTGGCACA-3'

(b): 5'-GCCGCTAATCTCCAGTGTACTCATTACCGCCGCTCCAGAA-3'

Engineering of stable HeLa COX8A-mNeonGreen cells

HeLa cells stably expressing COX8A-mNeonGreen were produced by CRISPR/Cas9-mediated incorporation of the donor plasmid AAVS1-Blasticidine-COX8A-mNeonGreen into the AAVS1 safe harbor locus as described previously (11). For cloning of the donor plasmid, AAVS1-Blasticidine-CAG-Flpe-ERT2 (Addgene #68461) was linearized using EcoRV and SalI restriction endonucleases. COX8A was amplified by PCR using oligonucleotides (c) and (d) and mNeonGreen was amplified by PCR using primers (e) and (f). All fragments were ligated using Gibson assembly®. AAVS1-Blasticidin-CAG-Flpe-ERT2 was a gift from Su-Chun Zhang (Addgene plasmid #68461; http://n2t.net/addgene:68461; RRID: Addgene_68461).

(c) 5´-ATTTTGGCAAAGAATTCGTCGACGCCGCCACCATGTCCGTC-3`

(d) 5´-CTCACCATGGTAAGCTTGATATCCTCTGGCCTC-3`

(e) 5´-AGCTTACCATGGTGAGCAAGGGCGAG-3`

(f) 5´-GATTATCGATAAGCTTGATATCTTACTTGTACAGCTCGTCCATG-3`

Viability-based phototoxicity assay of HeLa cells

HeLa cells were grown to 80-90 % confluency in a 96-well plate (costar 3599; Corning, NY, USA) before treatment. To achieve the same brightness after staining, HeLa cells were incubated with 1000 nM PKMO and 650 nM PKMO 0.9 respectively in DMEM (Gibco 11965092; Thermo Fisher Scientific) for 10 min at 37 °C with 5% CO_2. Then, the cells were washed with PBS three times and maintained in fresh medium for subsequent imaging analysis. 96-well plates were analyzed using a high-content imaging system ImageXpress Micro XLS (Molecular Devices, San Jose, CA, USA) equipped with a 20 ×/0.4 NA air objective and live-cell imaging device. The average light intensity on cells was measured to be 2.6 W/cm^2 (568 nm). The cells in each well were irradiated with the maximum light intensity at different time points. Time points were selected as 5, 7.5, 9, 12.5, 20, and 26.7 min (each time point with three parallel repeats). After illumination, the cells were directly used for subsequent imaging analysis. The cells were washed with PBS once and a 100 µL cell viability assay solution containing 1 µM Calcein AM (C2012-0.1ml; Beyotime, Shanghai, China) was added to each sample including controls (without dye incubation). Images of two channels (mitochondria: Cy3 channel, 568 nm; Calcein AM: FITC channel, 488 nm) were recorded on ImageXpress Micro XLS. The cell viability (%) was calculated according to the following equation: cell viability % = B / A *100%, where A = the number of total cells before illumination, and B =

the number of Calcein AM-positive cells after illumination. More than 500 cells were counted at each time point.

Comparison of the effects of PKMO and PKMO 0.9 on mitochondrial membrane potential
HeLa cells were co-stained with PKMO (250 nM) /Rho123 (300 nM) or with PKMO 0.9 (250 nM)/ Rho123 (300 nM) in DMEM for 1 h. Control cells were stained with Rho123 (300 nM) in DMEM for 1 h. Glass slides coated with polymer films containing 1 μM Rho123 were used as an in vitro control. All samples were recorded by time-lapse 2D STED nanoscopy (λ_{ex} = 488 nm and 561 nm. STED was performed at λ_{STED} = 775 nm). Each line was scanned 2 times and the signal was accumulated with dwell times offset to 10 μs. Fluorescence signal decrease was evaluated using Fiji. We measured the mean grey value for each frame after background subtraction. (Process > Math > Subtract; Image > Stacks > Measure Stack). Bleaching curves of individual measurements were normalized and averaged from four independent experiments.

Measurement of mitochondrial dehydrogenase activity
HeLa cells were grown to 80-90 % confluency in a 96-well plate (costar 3599, Corning) the day before treatment. Cells were either stained with 250 nM PKMO for different times (0.25, 0.5, 1, 2, 4, 12 h) or stained with different concentrations of PKMO for 14 hours at 37 °C with 5% CO_2. For non-illuminated samples, cells were washed with PBS once after the staining procedure and incubated with cultured medium containing 10% Cell Counting Kit-8 reagent (CCK8; C0038; Beyotime) for 3 hours at 37 °C with 5% CO_2. The absorbance (450 nm) of each well was recorded using a TECAN Infinite M Nano+ microplate reader (TECAN, Männedorf, Switzerland). For illuminated samples, the cells were seeded in 96-well plates and stained with PKMO (250 nM in DMEM, 30 min). The cells were washed once with PBS and then illuminated for different periods (0.25, 0.5, 1, 2 h) using a green LED (520-530 nm, 50 mW/cm^2) Mitochondrial dehydrogenase activity was measured by CCK8 assay as described before.

Real-time respirometry
Oxygen consumption rate (OCR) experiments were performed using a Seahorse XFe24 Extracellular Flux Analyzer (Agilent Technologies, Santa Clara, CA, USA). HeLa cells were seeded at 28,000 cells/ well and grown on the cell culture microplate (100777-004; Agilent Technologies) overnight. Seahorse XF cartridges were hydrated and calibrated by Seahorse XF calibrant (100840-000; Agilent Technologies). Cells were stained with PKMO (250 nM in DMEM) for 0, 2, 5, and 12 h. Negative controls were incubated with 10 μM carbonyl cyanide 3-chlorophenylhydrazone (CCCP) for 12 h. Baseline respiration was measured in XF DMEM Base Medium (103575-100; Agilent Technologies) with 4.5 g/L glucose and 2 mM glutamine after incubation at 37 °C in an incubator without CO_2 for 1 h. Periodic oxygen consumption measurements were performed, and OCR was calculated from the slope of change in oxygen concentration over time. Metabolic states were measured after subsequent addition of 1.5 μM oligomycin, 0.25 μM carbonyl cyanide 4 (trifluoromethoxy) phenylhydrazone (FCCP), 0.5 μM Rotenone/Antimycin A from Seahorse XF Cell Mito Stress Test Kit (103015-100; Agilent Technologies, MA, USA).

PKMO response to the treatment of mitochondrial oxidative phosphorylation uncoupler
HeLa cells were seeded in glass-bottom dishes (STGBD-035-1; Standard Imaging, Beijing, China) one day prior to imaging. Cells were stained with DMEM supplemented with 25 nM PKMO or 25 nM TMRE for 30 min. Cells were washed with medium once and maintained in a fresh medium for subsequent confocal imaging. Time-lapse confocal images were recorded at room temperature for 30 min after adding FCCP (10 μM) to the culture medium. The mitochondrial fluorescence intensities before and 30 min after FCCP addition were measured using Fiji (Process > Math > Subtract background; Image > Adjust > Threshold; Analysis > Analysis Particles > Multi-measure).

PKMO labeling for live-cell imaging of cancer cells, primary cells, and tissue.

General remarks on PKMO labeling

Like for other mitochondria-specific probes, the progression and intensity of PKMO labeling are dependent on the individual cell line and the culture condition. For optimal results, we recommend optimizing PKMO concentration and staining duration for each cell line. If long-term time-lapse imaging is desired, labeling density should be kept as low as possible to reduce potential phototoxic effects. For initial testing, we recommend staining cells using 250 nM PKMO for 15-20 min. If the signal is too weak, leading to noisy data or low intramitochondrial contrast, we recommend extending the staining time up to 1 hour. Similarly, the PKMO concentration can be increased up to 600 nM. Some cell lines, such as HeLa cells can show significant heterogeneity in PKMO labeling degree. This effect can be reduced by extending the staining duration at lower PKMO concentrations. Moreover, we observed that a washing step of 30 to 60 min following the staining procedure could significantly increase the intramitochondrial contrast while reducing unspecific ER labeling. For multi-color labeling, we recommend sequential staining if the used dyes require different incubation times. For a summary on PKMO concentration and labeling duration, please see Supplementary Tables S2 and S3.

Labeling of cancer cells

COS-7 cells were seeded in glass-bottom dishes (Standard Imaging) two days prior to imaging. COS-7 cells were stained with DMEM supplemented with 250 nM PKMO at 37 °C for 15 min. U-2 OS cells and HeLa cells were seeded in glass-bottom dishes (ibidi GmbH, Germany) one day prior to imaging. U-2 OS cells were stained with McCoy´s medium supplemented with 250 nM PKMO at 37 °C for 20 min. For single-color recordings, HeLa cells were stained with DMEM containing 150-250 nM PKMO at 37 °C for 40-45 min. Following the staining procedure, cells were washed three times with culture medium and incubated at 37 °C for 30-60 min to remove the unbound dye. The cells were imaged at room temperature in HEPES buffered DMEM (HDMEM) containing 4.5 g/l glucose, l-glutamine, and 25 mM HEPES (Thermo Fisher Scientific).

Labeling of primary cells

Primary brown adipocytes (pBAcs) were seeded in glass-bottom dishes (Standard Imaging) 3 days before imaging. pBAcs and primary hippocampal neurons were stained with DMEM containing 250 nM PKMO at 37 °C for 15 min. After removing the staining solution, the cells were washed with medium once and maintained in fresh medium for subsequent STED imaging. CMs were seeded in laminin-coated glass-bottom dishes 3 days before measurements. CMs were stained with 500 nM PKMO in DMEM at 37 °C for 15 min before imaging experiments. Islets tissues isolated from Ins1-Cre+/+; GCaMP6ffl/fl mice (7) were stained with Krebs-Ringer bicarbonate buffer (KRBB) solution containing 125 mM NaCl, 5.9 mM KCl, 2.4 mM $CaCl_2$, 1.2 mM $MgCl_2$, 1 mM L-Glutamine, 25 mM HEPES, 3 mM glucose, 0.1% (v/v) bovine serum albumin, and 600 nM PKMO at 37 °C for 30 min. After removing the staining solution, the islets were then washed with KRBB solution three times and maintained in a fresh medium for the STED imaging.

Sample preparation for multi-color imaging

Labeling of cristae and mtDNA in HeLa cells

For labeling of cristae and mtDNA, HeLa cells were incubated with DMEM containing 150 nM PKMO at 37° C for 45 min. Cells were washed twice and incubated with DMEM containing 0.5 µl/ml Quant-iT PicoGreen reagent (Thermo Fisher Scientific) at 37 °C for 30 min. The culture medium was replaced and the cells were incubated for 30 min to remove unbound dye. Cells were recorded in HDMEM at room temperature.

Labeling of cristae and SNAP/Halo fusion proteins

For labeling of MIC10-SNAP and cristae, HeLa cells were incubated with DMEM containing 200 nM PKMO at 37 °C for 40 min. Afterward, MIC10 was labeled with 1 µM SNAP-Cell 647-SiR (New England BioLabs Inc.) at 37 °C for 60 min. Halo-KDEL was labeled by co-staining with 300 nM PKMO and 500 nM 647-SiR-CA at 37 °C for 45 min. Following the staining procedure, cells were washed three times with culture medium and incubated at 37 °C for approximately 60 min to remove the unbound dye. The cells were imaged in HDMEM at room temperature. COS-7 cells expressing TOM20-Halo were incubated with

DMEM containing 500 nM 647-SiR-CA (synthesized according to literature protocol (12)) at 37 °C for 10 min. After removing the staining solution and three washing steps with PBS, the cells were then stained with DMEM containing 250 nM PKMO for 15 min at 37 °C. After removing the staining solution, the cells were then washed with DMEM once and maintained in fresh DMEM for following STED imaging at room temperature.

Labeling of cristae, tubulin, and mtDNA.
For labeling of cristae together with the cytoskeleton and mtDNA, HeLa cells were incubated with DMEM containing, 350 nM PKMO, 200 nM 4-610CP-CTX (13), and 0.2 µl/ml Quant-iT PicoGreen reagent (Thermo Fisher Scientific) at 37 °C for 40 min. Cells were washed three times with culture medium and incubated at 37 °C for 60 min to remove unbound dye. Cells were recorded in HDMEM at room temperature.

For a summary of used fluorophore concentrations and labeling duration, please also see Supplementary Tables S2 and S3.

Live-cell imaging of cancer cells, primary cells, and tissue.
For detailed summary of imaging parameters please see Supplementary Table S3. In brief, COS-7 cells, primary cells, and tissues were recorded using Facility Line or STEDYCON STED microscopes (Abberior Instruments GmbH, Göttingen, Germany) equipped with an Olympus UPlanXAPO 60x oil, NA1.42 objective (Olympus, Tokyo, Japan) or CFI Plan Apochromat Lambda D 100x oil, NA1.45 objective (Nikon, Tokyo, Japan). Pixel sizes of 20-30 nm were used for STED nanoscopy. PKMO was excited at 561 nm wavelength and STED was performed using a pulsed depletion laser at 775 nm wavelength with gating of 1-7 ns and dwell times of 10 µs. For dual-color STED imaging of COS-7 cells, SiR was excited at 640 nm wavelength. STED was performed at 775 nm wavelength with gating set to 0.75-8.75 ns. Dwell times of 10 µs were used. Rho123 was excited at 485 nm and recorded in the confocal mode. The fluorescence signal was usually accumulated over 2–5-line steps.
STED nanoscopy of HeLa and U-2 OS cells was carried out using an Expert Line dual-color STED 775 QUAD scanning microscope (Abberior Instruments GmbH). The microscope was equipped with a UPlanSApo 100×/1.40 Oil [infinity]/0.17/FN26.5 objective (Olympus). In brief, PKMO was excited at 561 nm and SiR was excited at 640 nm wavelength. Depletion was performed at a 775 nm wavelength. Imaging parameters were adjusted based on the individual samples. We typically used pixel sizes of 20 - 30 nm and dwell times of 5-7 µs. In the STED mode, each line was scanned 6 to 9 times and the signal was accumulated. PicoGreen, mEGFP and Rho123 were excited at 485 nm wavelength and were recorded in the confocal mode. The pinhole was set to 0.7 - 1.0 AU.

Comparison of PKMO and SNAP-cell SiR for cristae imaging
HeLa cells stably expressing COX8A-SNAP (11) were labeled with SNAP-cell SiR (1 µM in DMEM, 37 °C, 40 min). HeLa wild type cells were labeled with PKMO (250 nM in DMEM, 37 °C, 40 min). Afterward, cells were counterstained with DMEM supplemented with 1 µM Rho123 (Thermo Fisher Scientific) for 20 min at 37 °C. For controls, cells were stained only with Rho123. Cells were recorded by time-lapse 2D STED nanoscopy. For each individual cell, a single field of view (FOV) of 10 µm x 10 µm was recorded over 20 frames (total time: 317 seconds). Samples stained with PKMO/Rho123 were excited at λ_{ex} = 485 nm and 561 nm. Samples stained with SiR/Rho123 were excited at λ_{ex} = 485 nm and 640 nm. STED was performed at λ_{STED} = 775 nm. Overall, 15-line steps were performed (9-line accumulations in the STED mode (PKMO/SiR), 3-line accumulation in the confocal mode (PKMO/SiR), and 3-line accumulations in the confocal mode (Rho123). The pinhole was set to 0.8 AU. The pixel size was set to 30 nm. The pixel dwell time was set to 7 µs. For laser power settings, please see Supplementary Table S3.
Fluorescence signal decrease was evaluated using Fiji. We measured the mean grey value for each frame (Analyze > Set Measurements > Area & Mean Grey Value > OK; Image > Stacks > Measure Stack). Bleaching curves of individual measurements were normalized and averaged. The brightness of the PKMO staining was quantified in Fiji. To this end, the mitochondrial networks of the measured cells were manually selected on confocal overview images (80 x 80 µm FOV, 100 nm pixel size, 7 µs dwell time, 1x

line accumulation) and the maximum grey values were estimated (Analyze > Set Measurements > Area, Min & max gray value > OK; Image > Analyze > Measure).

Transmission electron microscopy

COS-7 cells were grown on Aclar® film (Electron Microscopy Sciences, Hatfield, PA) to a confluency of approximately 60-70%. Fixation was performed by immersion with pre-warmed 2.5% glutaraldehyde in 0.1M phosphate buffer (pH 7.4) at room temperature for 1h. After washing 3 times with 0.1 M phosphate buffer (pH 7.4), samples were post-fixed with 2% osmium tetroxide (w/v) and 1.5% potassium ferricyanide (w/v) in the same buffer at 4°C for 2 h and then washed 3 times. Following en bloc staining with 2% uranyl acetate (w/v) performed at 4°C overnight, the samples were dehydrated and embedded in fresh resin, polymerized at 65°C for 24 h. Ultrathin (70 nm) sections were obtained by a Leica UC7 ultramicrotome (Leica Microsystems, Wetzlar, Germany) and recorded on a JEOL Jem-1400 transmission electron-microscope (JEOL Ltd., Akishima, Japan) using a XAROSA CMOS camera (EMSIS GmbH, Münster, Germany)

Preparation of HeLa and U-2 OS cells was performed as described previously (8). In brief, cells were grown on Aclar® film (Plano GmbH, Wetzlar, Germany) to a confluency of approximately 70%. Fixation was performed by immersion with pre-warmed (37 °C) 2% glutaraldehyde in 0.1 M cacodylate buffer (pH 7.4) at room temperature. For complete fixation, samples were stored at 4°C overnight. Following post-fixation with 1% osmium tetroxide and pre-embedding staining with 1% uranyl acetate, samples were dehydrated and resin-embedded. Ultrathin sections (70 nm) were recorded on a Talos L120C transmission microscope (Thermo Fisher Scientific, Hilsboro, Oregon, USA) at 11,000-13,500× magnification using a Ceta 4k × 4k CMOS camera (Thermo Fisher Scientific).

Primary neurons were grown on Aclar® discs (Electron Microscopy Sciences) to a confluency of approximately 70-80%. Fixation was performed by immersion with pre-warmed 3% glutaraldehyde in 0.1M phosphate buffer (pH 7.4) at 4°C for 2 h. After washing 3 times with 0.1 M phosphate buffer (pH 7.4), samples were post-fixed with 1% osmium tetroxide (w/v) in 0.24 M phosphate buffer (pH 7.4) at 4°C for 2 h and then washed 3 times. The samples were dehydrated and embedded in fresh resin, polymerized at 60°C for 24 h. Ultrathin (60-70 nm) sections were obtained by a Leica UC7 ultramicrotome (Leica Microsystems). Thin sections laid on copper mesh were stained with heavy metals, uranyl acetate, and lead citrate for contrast. A Hitachi-H7800 transmission electron microscope (Hitachi High-Technologies Corporation, Chiyoda, Japan) was used to observe the hippocampal ultrastructure.

Apoptosis by expression of mEGFP-BAX

Oligonucleotides (g) and (h) were used for site directed mutagenesis PCR of pEGFP-C3 (Clontech Laboratories Inc., Mountain View, CA, USA) to produce the expression plasmid pmEGFP-C3. BAX was amplified by PCR using oligonucleotides (i) and (j) and incorporated into pmEGFP-C3 using HindIII and EcoRI restriction sites.

(g) 5´-CTACCTGAGCACCCAGTCCAAGCTGAGCAAAGACCCCAACG-3´
(h) 5´-CGTTGGGGTCTTTGCTCAGCTTGGACTGGGTGCTCAGGTAG-3´
(i) 5´-TTAAAGCTTATGGACGGGTCCGGGGAG-3´
(j) 5´-AATGAATTCTCAGCCCATCTTCTTCCAGA-3´

HeLa cells were seeded in glass-bottom dishes (Ibidi GmbH) and were stained with 250 nM PKMO (DMEM, 37 °C, 45 min) the next day. Afterward, cells were transfected with pmEGFP-C3-BAX using jetPRIME® transfection reagent (Polyplus). Q-VD-Oph hydrate (APExBIO Technology LLC, Houston, TX, USA) was added to the medium at a concentration of 20 µM immediately after transfection in order to prevent detachment of apoptotic cells. Cells were analyzed by 2D STED nanoscopy 4-6 h after transfection. Cells were recorded in HDMEM at room temperature.

Image processing

STED nanoscopy

STED nanoscopy images of COS-7 cells, primary cells, and tissues were deconvoluted using Huygens software (Scientific Volume Imaging B.V., Hilversum, The Netherlands). STED nanoscopy images of HeLa and U-2 OS cells and 3D STED images of COS 7 cells were deconvoluted using the Richardson-

Lucy algorithm in the Imspector software (Abberior Instruments GmbH; version 0.14.11616). 3D rendering and reconstruction of 3D STED data were performed using Imaris (Bitplane, Belfast, UK). Resolution estimation was performed by Gaussian fitting of fluorescence intensity line profiles. The full width at half maxima (FWHM) was estimated via Analysis >> Fitting >> Nonlinear curve fit >> Gaussian fit in the Origin Pro 2020b software (OriginLab Corporation, Northampton MA, USA) or using Matlab (The Math Works, Natick, MA, USA). All images that were used to analyze the resolution in supplementary information were raw data without background subtraction. For time-lapse recordings, photobleaching was compensated using the bleach correction feature in Fiji/ImageJ (version 1.53f51).

Electron microscopy
Electron microscopy recordings were filtered using a median filter in Fiji/ImageJ (version 1.53f51).

Analysis of mitochondrial network appearance
HeLa cells stably expressing COX8A-mNeonGreen were stained at 37 °C for 30 minutes using DMEM supplemented with 250 nM of PKMO, PKMR, Mitotracker Red (Thermo Fisher Scientific) or TMRE (Thermo Fisher Scientific), respectively. Controls were treated with 20 µM FCCP for 30 min at 37 °C to induce fragmentation of mitochondria. Cells were washed three times using DMEM and recorded 2 hours and 5 hours later using a Facility Line confocal microscope (Abberior Instruments). The cells were recorded at 37°C and 5% CO_2. Tile scans (500 µm x 500 µm, 10% overlap) were recorded with a pixel size of 100 nm. The pinhole was set to 1.0 AU. mNeonGreen was excited at λ_{ex} = 485 nm, the mitochondrial probes were excited at λ_{ex} = 561 nm. The pixel dwell time was set to 6.5 µs and 2-line accumulations were recorded for each channel. The mNeonGreen channel was utilized for segmentation of individual cells using a machine learning approach. For optimized cell detection, the cell shape was manually determined on about 10% of the images. The manually labeled recordings served as input for an image-to-image translation network (14) or training. After applying the trained network on all recordings (using the mNeonGreen channel) minor corrections were applied manually (like cutting a residual connection between two adjacent cells or joining fragments to cover a whole cell). Overall, the cell shape detection (data not shown) could distinguish the vast majority of cells at high accuracy. Therefore, we used the segmentation data to correlate the fluorescence readout on a single cell level. The structure of the mitochondrial network was detected and analyzed using a custom written script in MATLAB (The MathWorks Inc., Natick, MA, USA). First, the confocal images were deconvolved (Richardson-Lucy) with a 200nm FWHM Gaussian peak PSF and a relative threshold above the background (10% of maximum) was applied to get a binary representation of the mitochondrial network. For the analysis of the variances of the cellular brightness (of mNeonGreen, PKMO, PKMR, Mitotracker Red or TMRE) the average signal intensity was calculated in each color channel on the detected mitochondria for each individual cell. The results were then pooled for each recording and normalized by the mean of all cells of the recording. Therefore, all brightness distributions show the cellular brightness relative to the mean brightness per cell per recording. For the estimation of eccentricity, the detected mitochondrial network was divided into disjoint segments. For each segment, the eccentricity of the ellipse that has the same second-moments as the segment was calculated (MATLAB function *regionprops*). For the determination of the average mitochondrial network branch length, the skeleton of the detected mitochondrial network per cell was calculated, branch points were eliminated and the average length of the remaining disjoint curves was calculated.
.

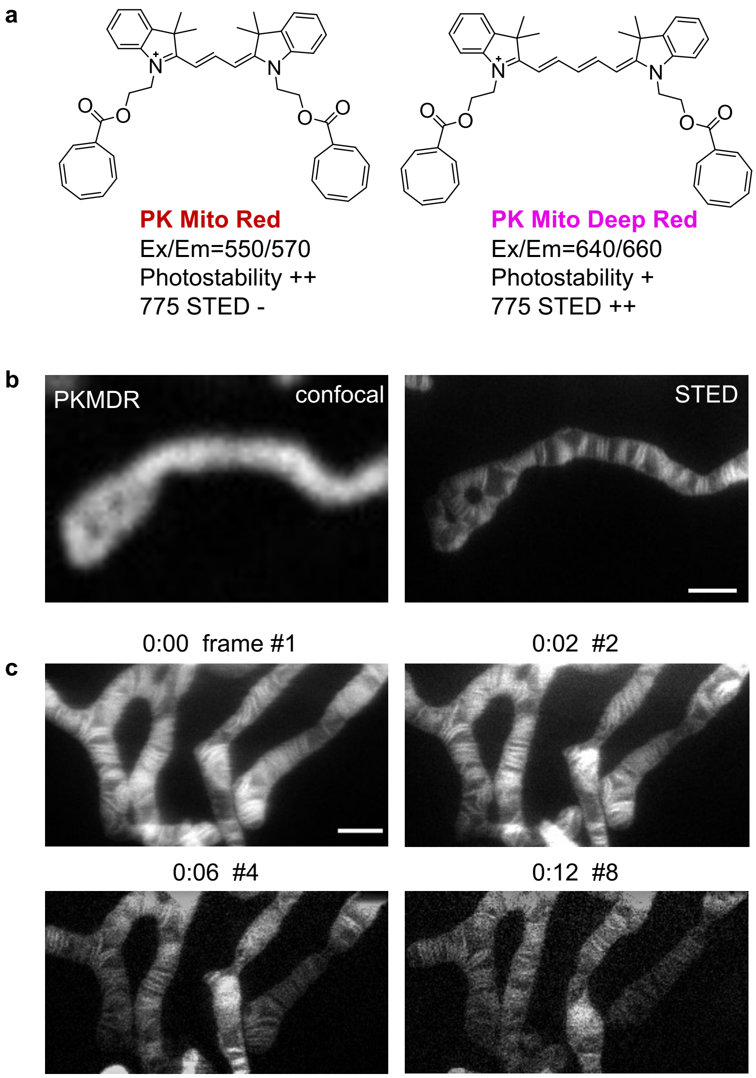

Figure S1. STED nanoscopy of mitochondria labeled with PKMDR. (a) Chemical structures of PK Mito Red (PKMR) and PK Mito Deep Red (PKMDR). (b) Confocal (left) and STED (right) image of mitochondria in live COS-7 cells labeled with 250 nM PKMDR for 15 min. Scale bar = 1 μm. (c) Time-lapse images of cristae in a live COS-7 cell labeled with PKMDR (250 nM, 15 min). Scale bar = 1 μm.

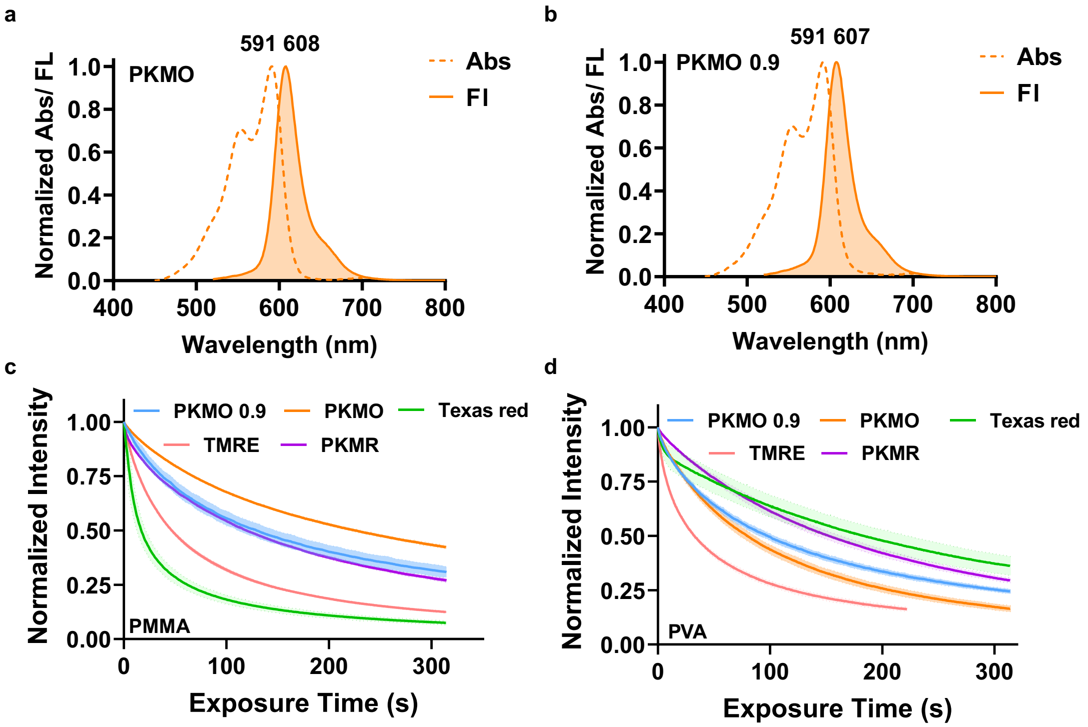

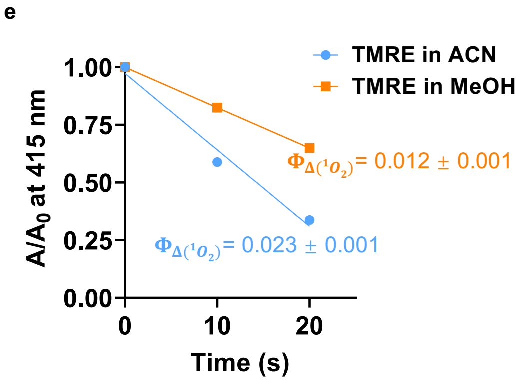

f

	PKMO	PKMO 0.9
**Abs/Em**	591/608[a]	591/607[a]
**Quantum yield (Φ_f)**	0.14[a] / 0.45[b]	0.13[a] / 0.49[b]
**$\Phi_{\Delta(^1O_2)}$ (x 10^{-4})**	67 ± 5[c]	17 ± 1[c]

Figure S2. Photophysical properties of PKMO. (a-b) Absorption (abs) and fluorescence (fl) spectra of PKMO (a) and PKMO 0.9 (b) in methanol. (c-d) Photobleaching curve of PKMO, PKMO 0.9, PKMR, Texas Red, TMRE in polymethyl methacrylate (PMMA) (c) and polyvinyl alcohol (PVA) film (d) recorded under continuous 561 nm laser scanning of a confocal microscope. (e) Singlet oxygen quantum yield of TMRE in MeOH or ACN measured using 1,3-diphenylisobenzofuran (DPBF) decay assay. TMRE in MeOH (Φ_Δ= 0.012) in MeOH was selected as a standard. (f) table of photophysical properties of PKMO and PKMO 0.9. Solvents: a, methanol; b, toluene; c, acetonitrile.

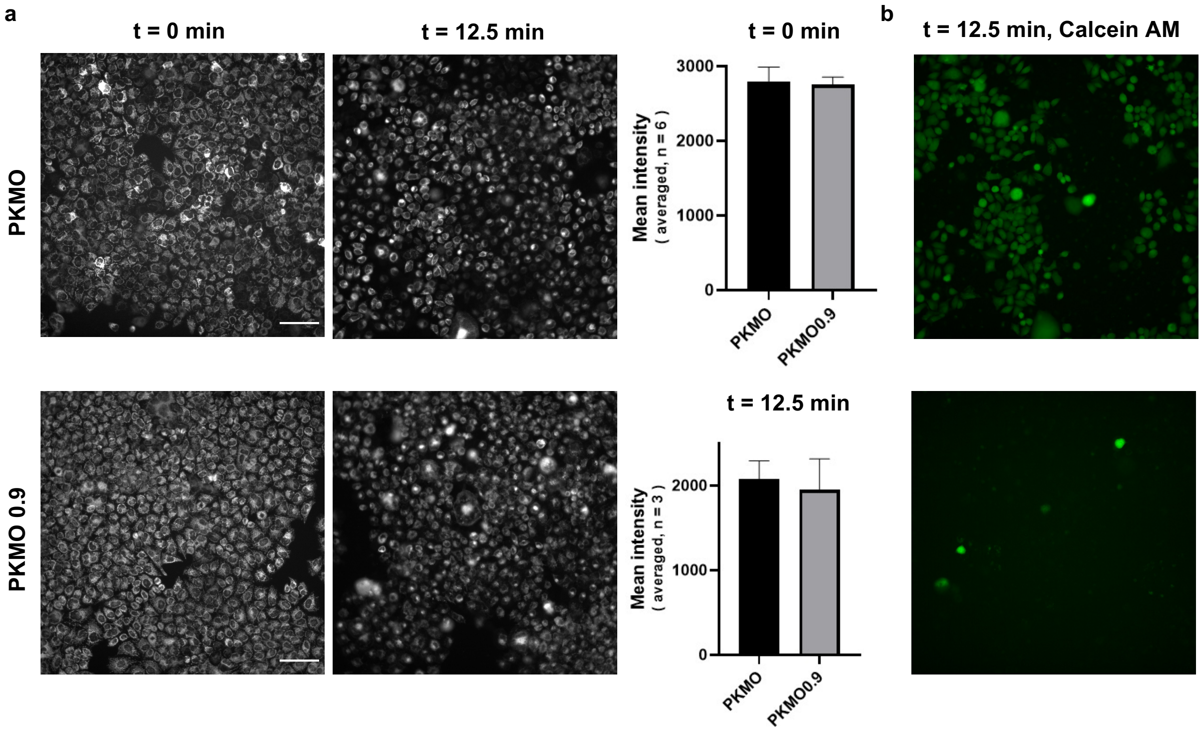

Figure S3. Phototoxicity of PKMO and PKMO 0.9. (a) Wide field images of live HeLa cells labeled with 1 μM PKMO or 0.65 μM PKMO 0.9 for 15 min before (left) and after LED illumination in the high-content imaging system and analysis of mean mitochondrial fluorescence intensity before and after 12.5 min illumination. Error bars represent standard deviation. (b) Wide field images of Calcein AM (2 μM), an indicator of cell viability, after 12.5 min illumination of PKMO or PKMO 0.9 treated cells.

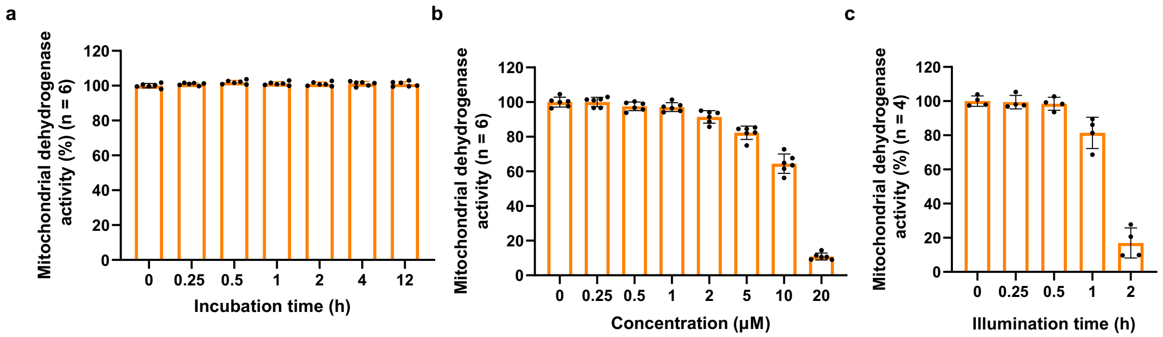

Figure S4. Mitochondrial dehydrogenase activity of HeLa cells stained with PKMO at different conditions. (a) Mitochondrial dehydrogenase activity of HeLa cells stained with 250 nM PKMO for different incubation times. Bars indicate the mean of six individual repeats. Error bars indicate standard deviation. (b) Mitochondrial dehydrogenase activity of HeLa cells treated by PKMO at different concentrations for 14 hours. Bars indicate the mean of six individual repeats. Error bars indicate standard deviation. (c) Mitochondrial dehydrogenase activity of PKMO-treated (250 nM, 30 min) HeLa cells illuminated by 50 mW/cm^2 green LED (520 - 530 nm) for different illumination times. Bars indicate the mean of four individual repeats. Error bars indicate standard deviation.

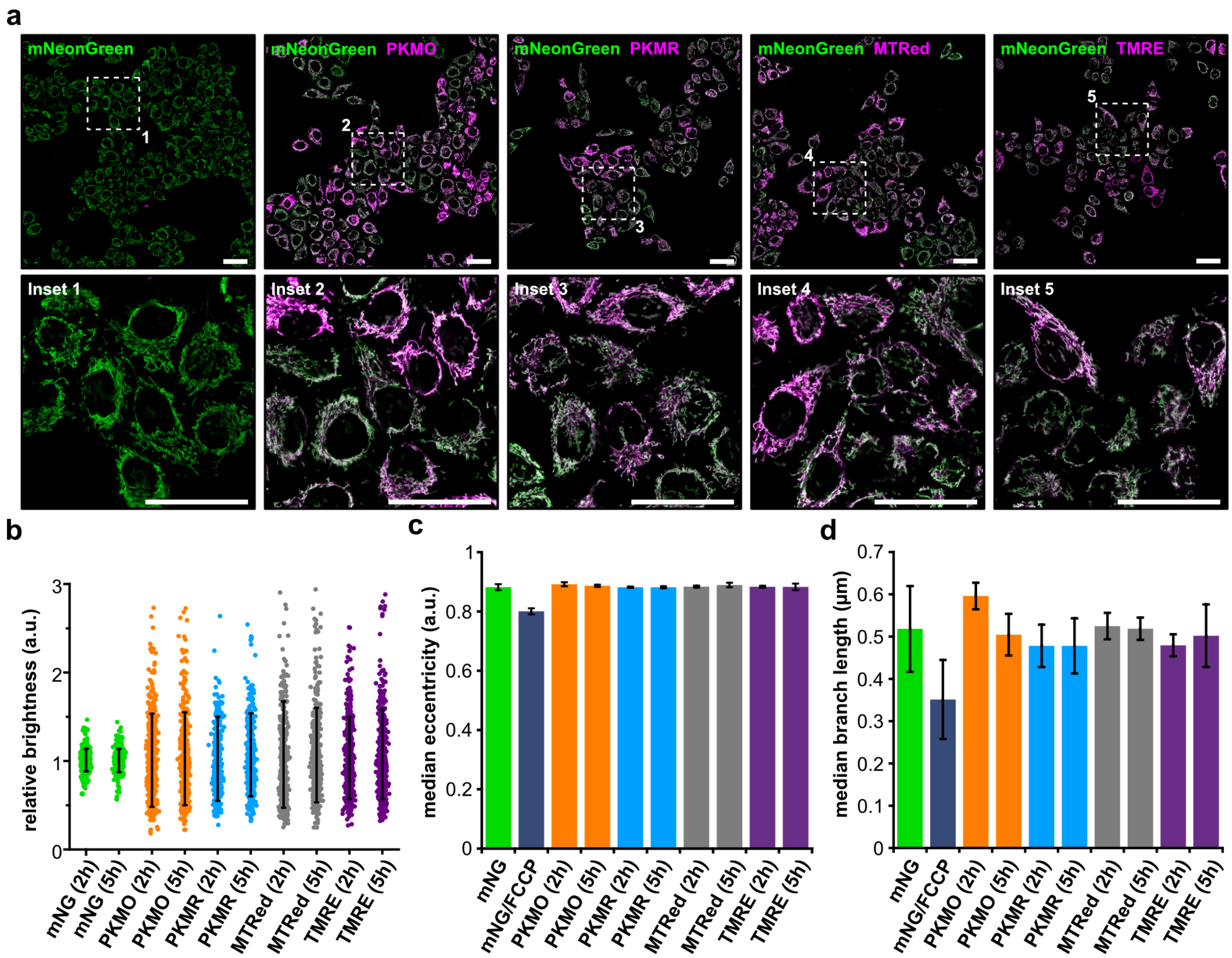

Figure S5. Heterogeneity of PKMO staining and influence of PKMO on the mitochondrial network appearance. (a-d) HeLa cells expressing COX8A-mNeonGreen (mNG) stained with different mitochondria-specific probes (250 nM, 37 °C, 30 min). Confocal microscopy images were recorded 2h and 5h after staining. (a) Representative recordings of HeLa cells 5h after staining. (b-d) Single cells and their respective mitochondrial networks were automatically segmented and analyzed. (b) Comparison of the relative brightness of individual cells. At least 380 cells were analyzed for each sample. Bars indicate 10-90 percentile range. (c) Analysis of the median eccentricity of segmented mitochondria. Bars indicate the average of three individual biological repeats (at least 68 cells were analyzed per sample and repeat). Error bars indicate the standard deviation. Control (mNG/FCCP) was treated with 20 µM FCCP for 20 min to induce mitochondrial fragmentation. (d) Analysis of the median branch length of automatically segmented mitochondrial sub-networks. Bars indicate the average of three independent biological repeats (at least 68 cells were analyzed per sample and repeat). Control (mNG/FCCP) was treated with 20 µM FCCP for 20 min to induce mitochondrial fragmentation. Error bars indicate the standard deviation. Scale bars: 50 µm.

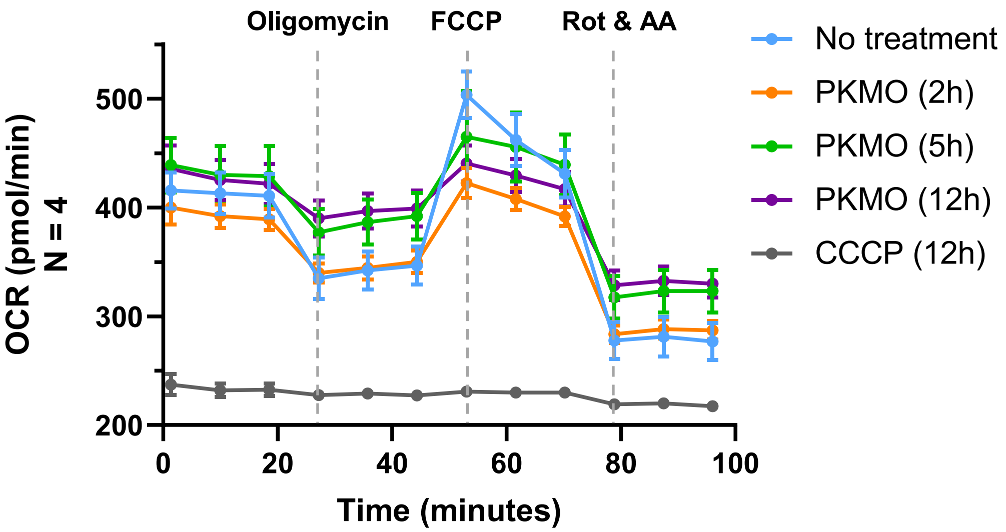

Figure S6. Real-time respirometry of HeLa cells. Real-time oxygen consumption rate (OCR) of HeLa cells without treatment (blue) and PKMO treatment (250 nM in DMEM) for different times (2h, orange; 5h, green; 12h, purple). OCR was monitored after addition of oligomycin (1.5 μM), carbonyl cyanide 4-(trifluoromethoxy) phenylhydrazone (FCCP, 0.25 μM) and rotenone/antimycin a (AA, 0.5 μM). The gray curve shows negative controls treated with 10 μM carbonyl cyanide m-chlorophenylhydrzone (CCCP) for 12 h. Four independent repeats were averaged and analyzed for each sample. Error bars indicate standard error of mean (SEM).

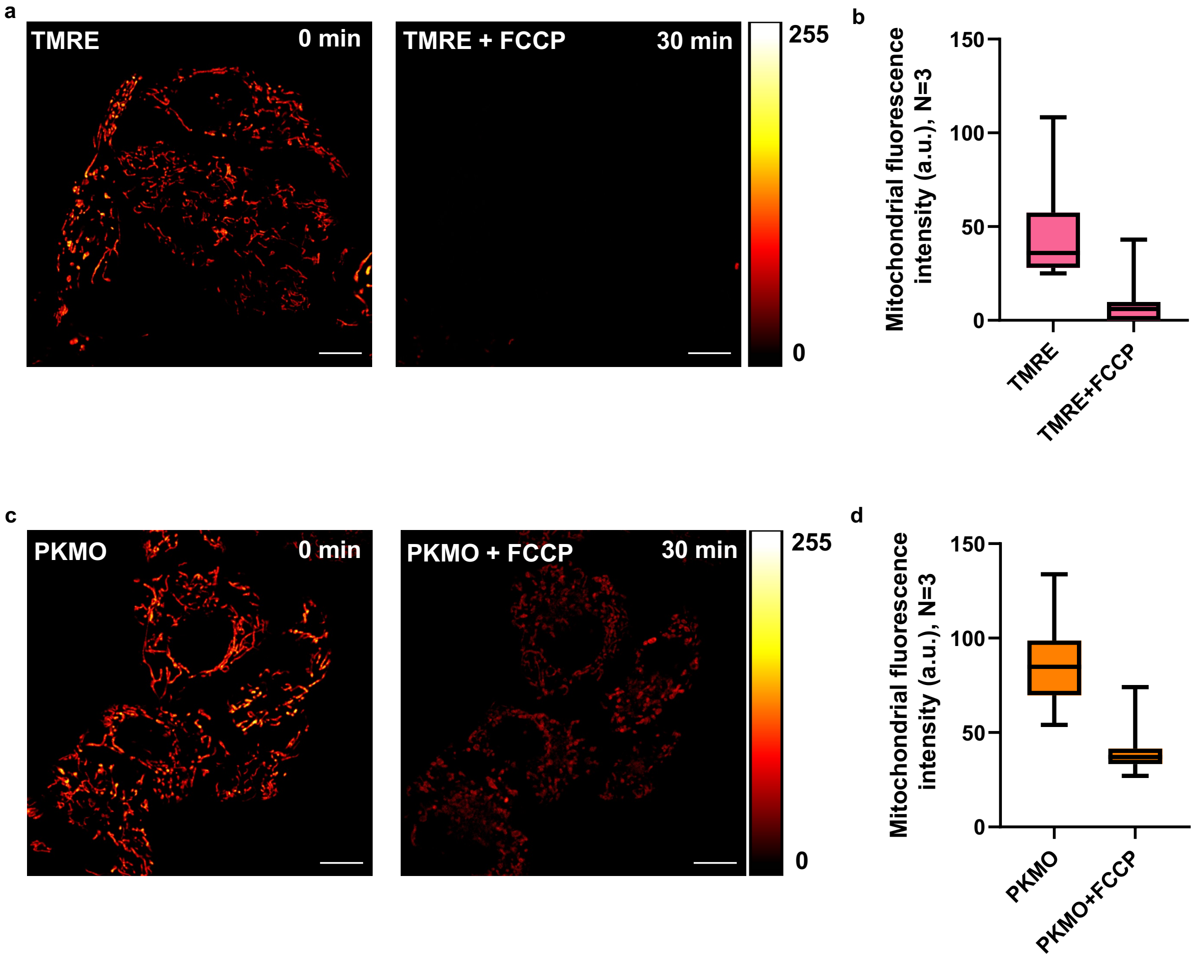

Figure S7. Influence of the mitochondrial membrane potential on PKMO labeling. (a-d) HeLa cells were labeled with DMEM supplemented with 25 nM TMRE or 25 nM PKMO, respectively. Cells were then treated with FCCP (10 µM in DMEM) for 30 min to decouple the mitochondria. (a,c) Confocal images were recorded before and 30 min after FCCP addition. (b, d) Analysis of mitochondrial fluorescence intensity from recordings presented in (a and c) The box and the central line indicate the 25-75 percentile and median, respectively. The upper and lower whisker indicate the maximum and minimum. Scale bars = 10 µm.

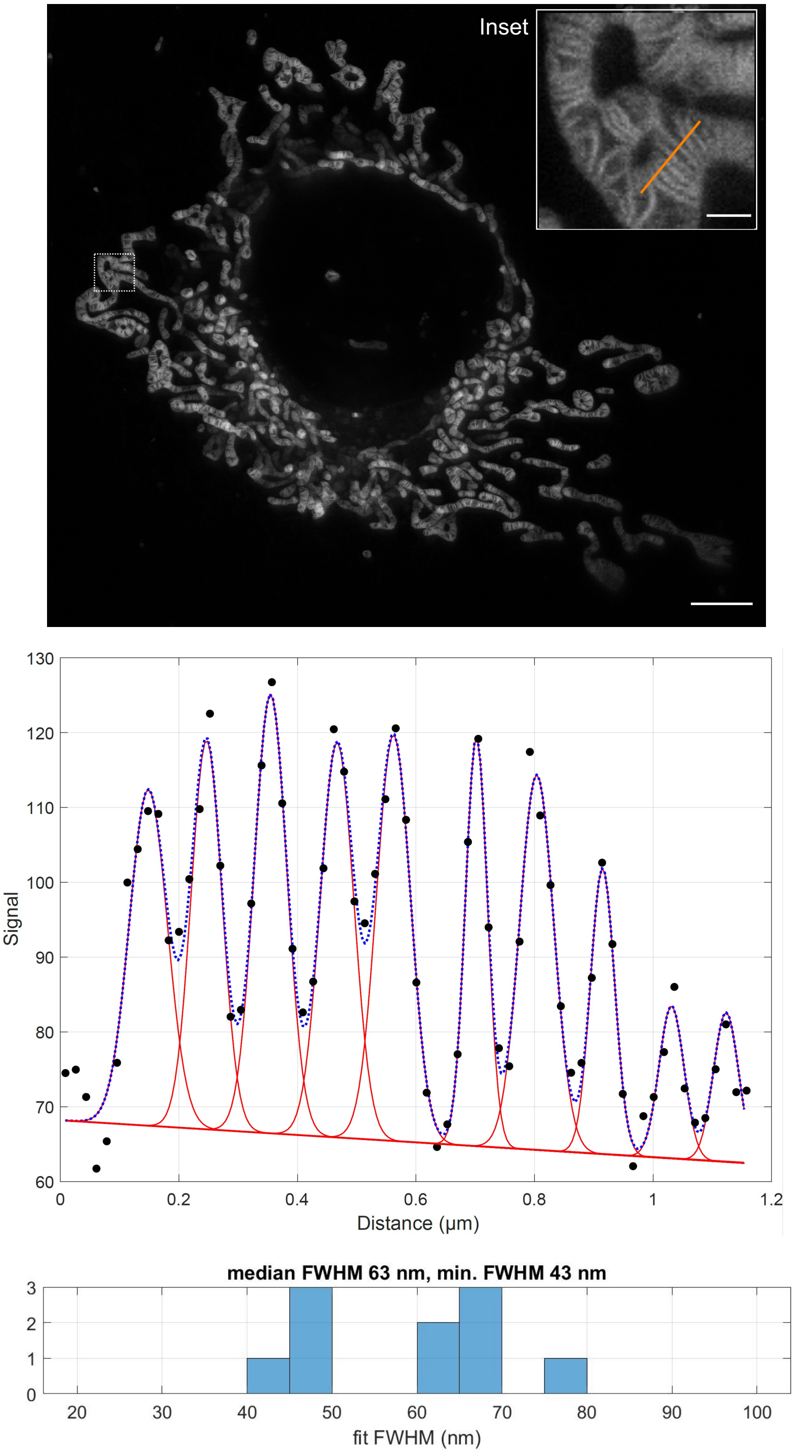

Figure S8. Resolution estimation of 2D STED nanoscopy. Top: raw data (SNR=15) of the 2D-STED recording of a live COS-7 cell shown in Fig. 2. Inset (white boxed area) indicates the area used for resolution estimation. Bottom: fluorescence intensity line profile was measured as indicated by the orange line. The fluorescence intensity signal was fitted using a Gaussian fit. The full width at half maximum (FWHM) was estimated for the individual peaks. Scale bar: overview 5 µm, inset 2 µm.

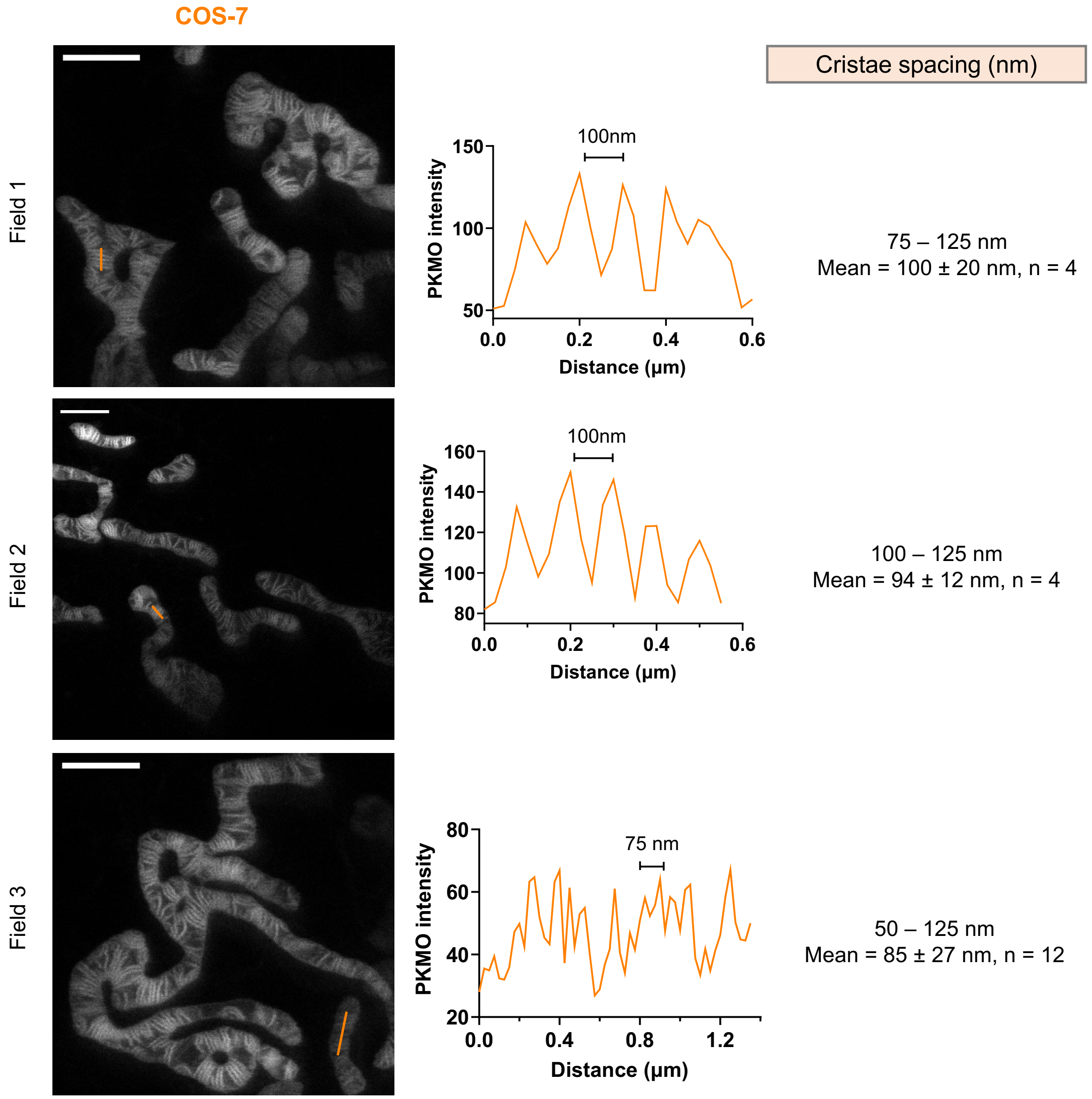

Figure S9. Cristae spacing in COS-7 cells. STED images of mitochondrial cristae in live COS-7 cells labeled with PKMO (left) and fluorescence intensity line profiles (right). The fluorescence intensity line profiles were measured as indicated by the orange lines in the STED images and used to estimate the cristae distances. Scale bars = 2 µm.

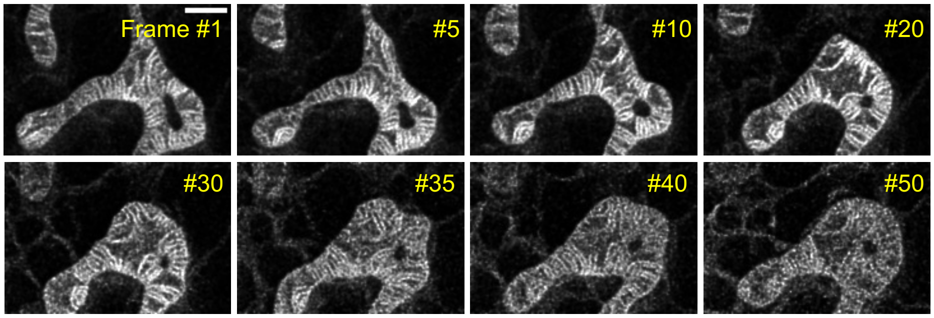

Figure S10. Time-lapse 2D STED nanoscopy of COS-7 cells. Shown are selected frames from a 50-frame time-lapse recording of cristae in a live COS-7 cell labeled with PKMO (250 nM,15 min). Scale bar = 1 μm.

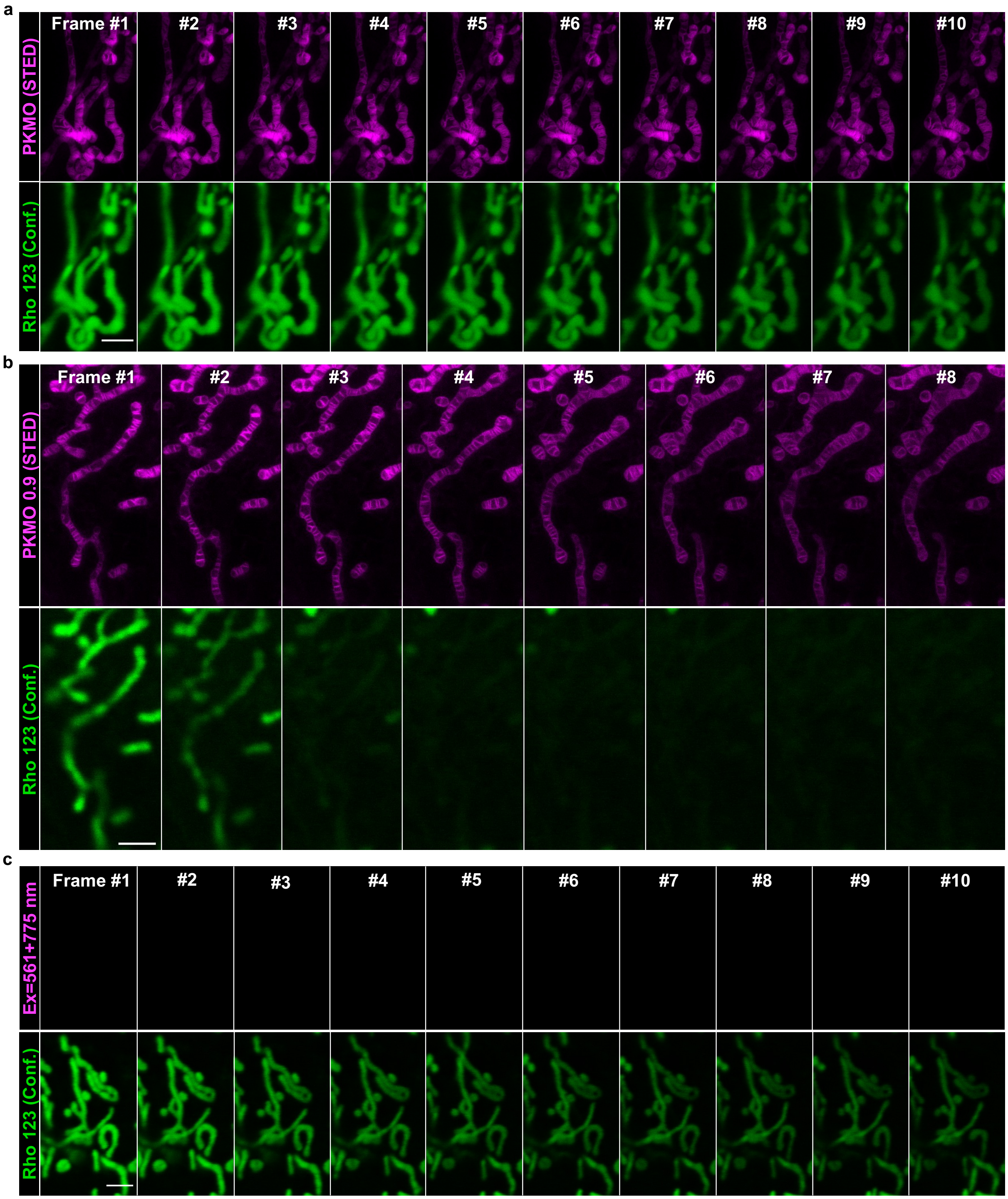

Figure S11. Comparison of PKMO and PKMO 0.9 for live-cell STED nanoscopy of mitochondrial cristae. (a-c) HeLa cells were co-labeled with PKMO or PKMO 0.9 (250 nM) and Rho123 (300 nM) in DMEM for 60 min. PKMO was recorded by STED nanoscopy, Rho123 was recorded in the confocal mode. (a) Dual-color time-lapse recording of cells co-stained with PKMO (STED) and Rho123 (confocal). (b) Dual-color time-lapse recording of HeLa co-stained with PKMO 0.9 (STED) and Rho123 (confocal). (c) Time-lapse recording of HeLa cells stained with Rho123 only (confocal). All samples were recorded with two excitation/emission channels (λ_{Ex} 488 nm (green); λ_{Ex} = 561 nm (magenta). Depletion was performed at λ_{STED} = 775 nm). Scale bars = 2 µm.

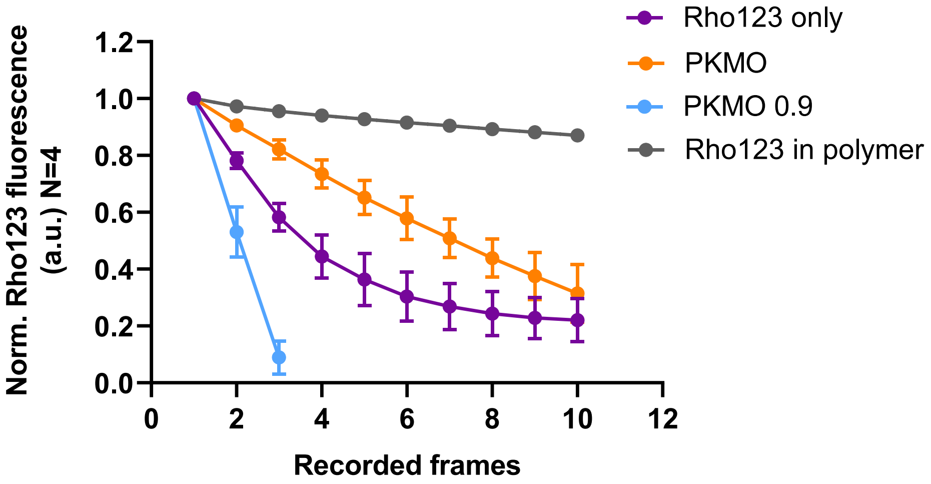

Figure S12. Photodamage by STED imaging of PKMO and PKMO 0.9 as evaluated by Rho123 fluorescence in confocal channel. HeLa cells were co-labeled with PKMO or PKMO 0.9 (250 nM) and Rho123 (300 nM) in DMEM for 60 min. PKMO was recorded by STED nanoscopy, Rho123 was recorded in the confocal mode. Cellular control (purple curve) shows cells which were labeled with Rho123 only (300 nM, DMEM, 60 min). In vitro control (gray curve) shows data from glass slides coated with polymer films (PMMA) containing 1 µM Rho123. All samples were recorded with two excitation/emission channels (λ_{Ex} 488 nm (green); λ_{Ex} = 561 nm (magenta). Depletion was performed at λ_{STED} = 775 nm). Curves show averaged bleaching curve of four fields of view. Error bars show standard deviation.

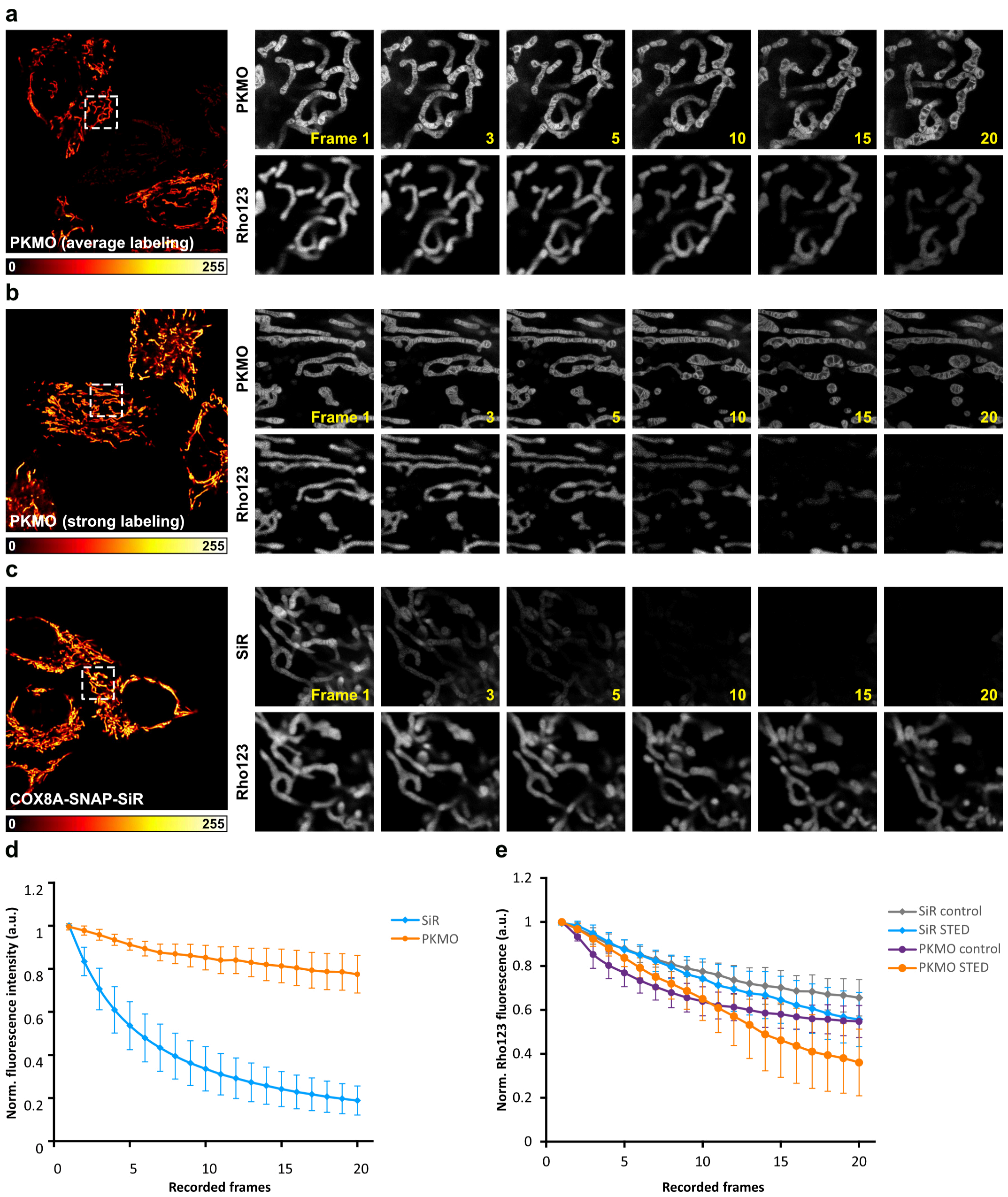

Figure S13. Comparison SNAP-cell SiR and PKMO for live-cell STED nanoscopy of mitochondrial cristae. (a-e) HeLa cells stably expressing COX8A-SNAP and HeLa cells (wild type) were labeled with SNAP-cell SiR (1 µM, 40 min) or PKMO (250 nM, 40 min), respectively. Cells were counterstained using Rho123 (1 µM, 20 min). SNAP-cell SiR and PKMO were recorded by STED nanoscopy, Rho123 was recorded in the confocal mode. Left: confocal overviews. Right: Consecutive frames from the areas indicated by the dashed boxes. (a) Time-lapse STED nanoscopy of a cell with average PKMO labeling degree. (b) Time-lapse STED nanoscopy of a cell with strong PKMO labeling. (c) Time-lapse STED of a COX8A-SNAP expressing cell labeled with SNAP-cell SiR. (d) Evaluation of SNAP-cell SiR and PKMO photobleaching during STED imaging. Curves show averaged bleaching curves of 20 cells (recorded during 2 individual biological repeats). Error bars represent standard deviation. (e) Evaluation of Rho123 fluorescence during STED imaging. All samples (including controls) were recorded by STED-nanoscopy (PKMO: λ_{ex} 561 nm, λ_{ex} 488 nm, λ_{STED} 775 nm; SiR: λ_{ex} 640 nm, λ_{ex} 488 nm, λ_{STED} 775 nm). Curves show averaged bleaching curves of at least 10 individual cells (from 2 independent biological repeats). Error bars show standard deviation.

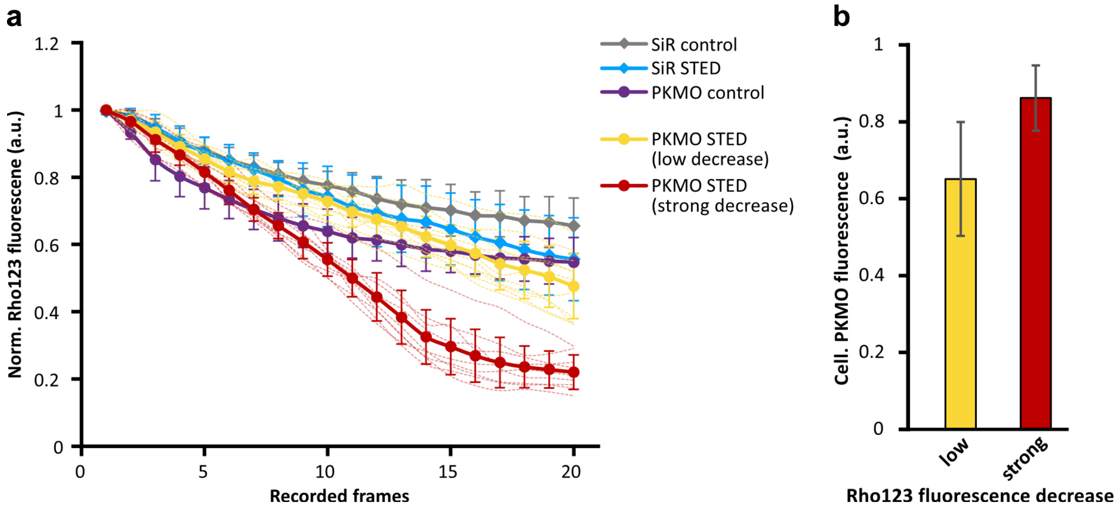

Figure S14. Photodamage by STED imaging of PKMO. (a-b) HeLa cells stably expressing COX8A-SNAP and HeLa wild type cells were labeled with SNAP-cell SiR (1 µM, 40 min) or PKMO (250 nM, 40 min), respectively. Cells were counterstained using Rho123 (1 µM, 20 min). SNAP-cell SiR and PKMO were recorded by STED nanoscopy, Rho123 was recorded in the confocal mode. (a) Evaluation of Rho123 fluorescence during STED imaging. All samples (including controls) were recorded by STED-nanoscopy (PKMO: λ_{Ex} 561 nm, λ_{Ex} 488 nm, λ_{STED} 775 nm; SiR: λ_{Ex} 640 nm, λ_{Ex} 488 nm, λ_{STED} 775 nm). Data points represent averaged bleaching curves of at least 10 individual cells (from 2 independent biological repeats). Error bars show standard deviation. Dashed curves show individual measurements. Yellow curves: Cells with low decrease in Rho123 fluorescence. Red curves: Cells with strong decrease in Rho123 fluorescence. (b) Quantification of maximum cellular PKMO fluorescence signal of cells that showed low (yellow bar) or strong decrease (red) of Rho123 fluorescence signal. Bars show the mean of 10 individual measurements. Error bars indicate standard deviation.

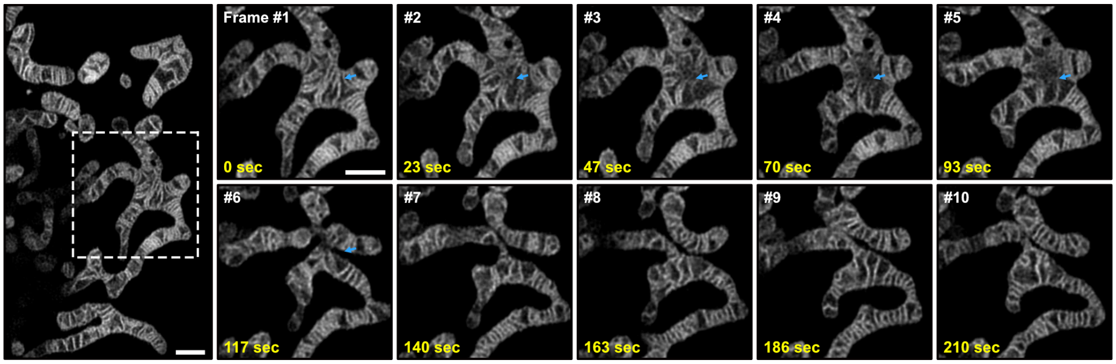

Figure S15. Cristae remodeling during mitochondrial tubule fission. Time-lapse STED nanoscopy recording of mitochondria in a HeLa cell. Left: Overview. Right: Time-lapse recording of the area indicated by the dashed box. Shown are ten consecutive frames of a branched mitochondrion that undergoes fission. Arrows indicate cristae, which undergo significant remodeling before and during the separation of the mitochondria. Scale bars = 1 µm.

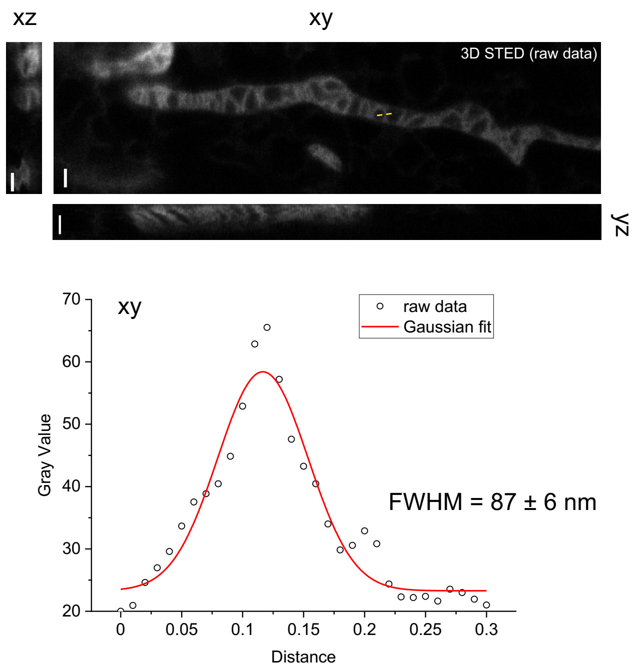

Figure S16. 3D STED nanoscopy of COS-7 cells. Orthogonal view of a 3D-STED recording of a mitochondrion in a live COS-7 cell presented in Movie S2 and Fig. 3. The image was recorded using a 3D STED PSF and a voxel size of 20 x 20 x 50 nm. The fluorescence intensity line profile was measured as indicated by the yellow line. Fluorescence intensity was fitted using a Gaussian fit and FWHM was estimated. Scale bars = 500 nm.

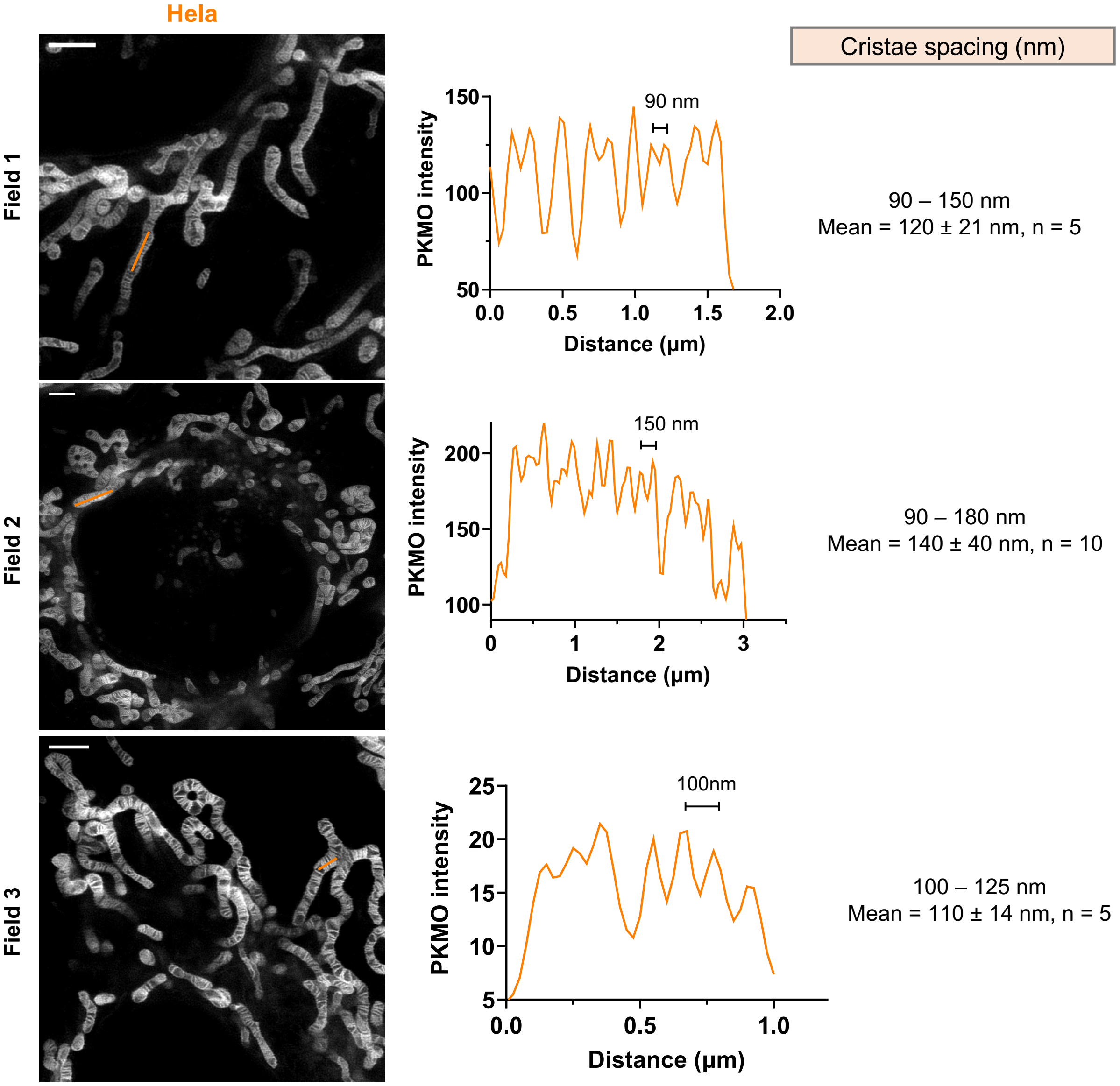

Figure S17. Cristae spacing in HeLa cells. STED images of mitochondrial cristae in live HeLa cells labeled with PKMO (left) and fluorescence intensity line profiles (right). The fluorescence intensity line profiles were measured as indicated by the orange lines in the STED images and used to estimate the cristae distances. Scale bars = 2 μm.

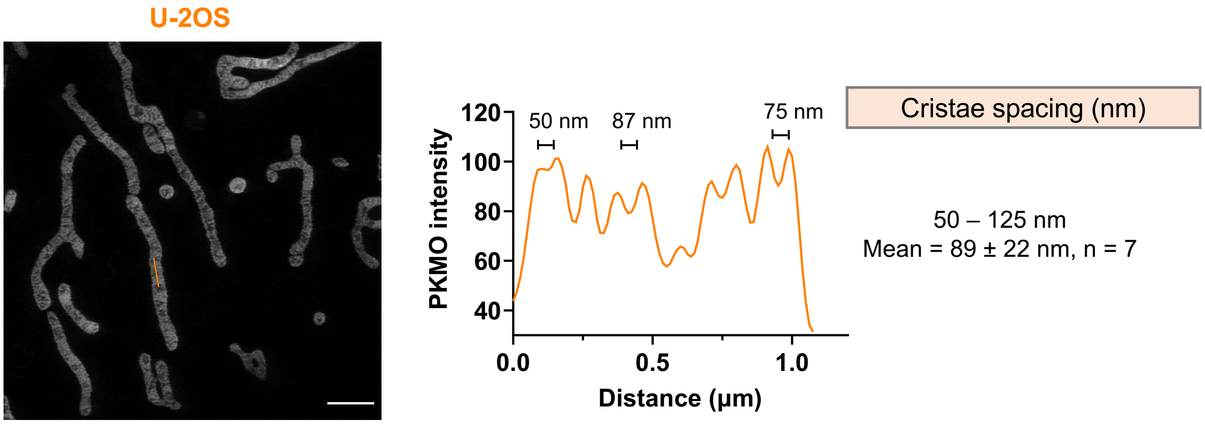

Figure S18. Cristae spacing in U-2 OS cells. STED image of mitochondrial cristae in a live U-2 OS cell labeled with PKMO (left) and fluorescence intensity line profiles (right). The fluorescence intensity line profiles were measured as indicated by the orange lines in the STED images and used to estimate the cristae distances. Scale bars = 2 μm.

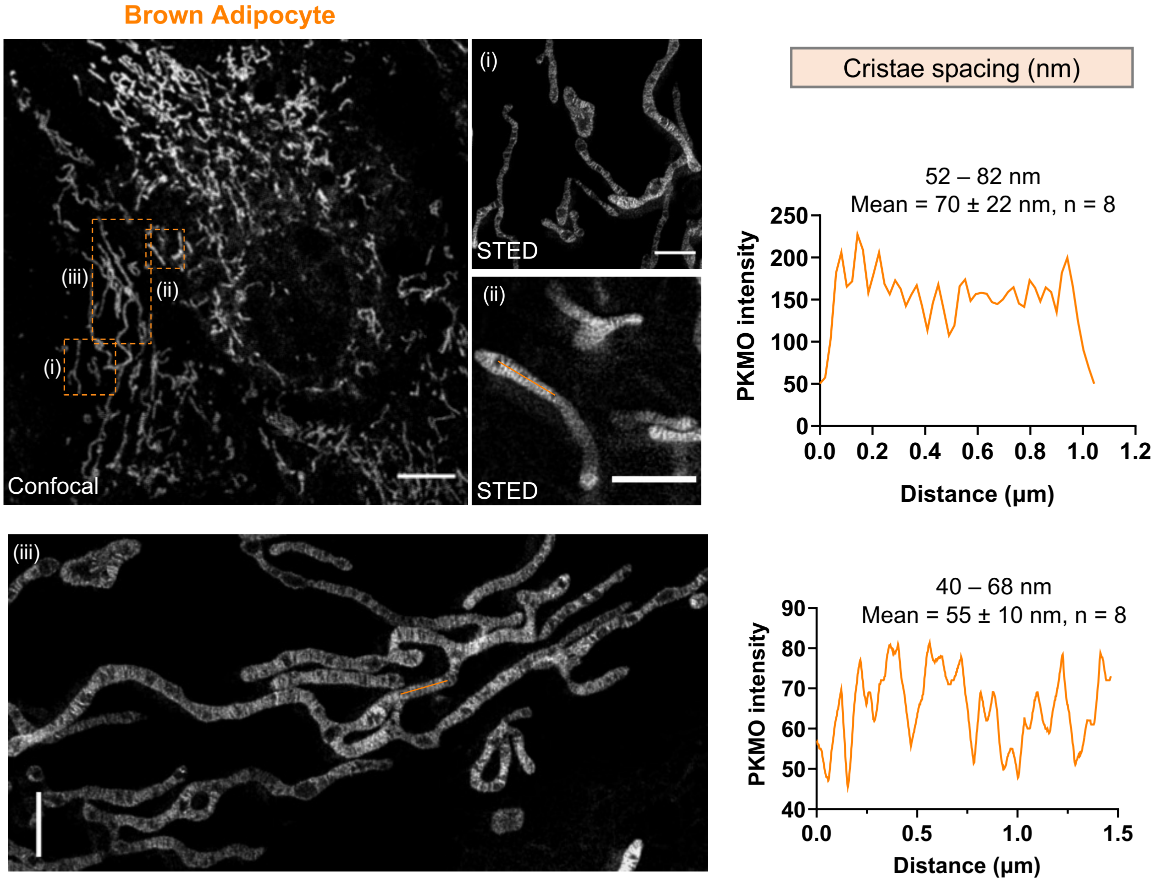

Figure S19. Cristae spacing in brown adipocytes. STED images of mitochondrial cristae in live primary brown adipocytes (pBAcs) labeled with PKMO (250 nM,15 min) (left) and fluorescence intensity line profiles (right). The fluorescence intensity line profiles were measured as indicated by the orange lines in the STED images and used to estimate the cristae distances. Scale bar: overview 5 µm, insets 2 µm.

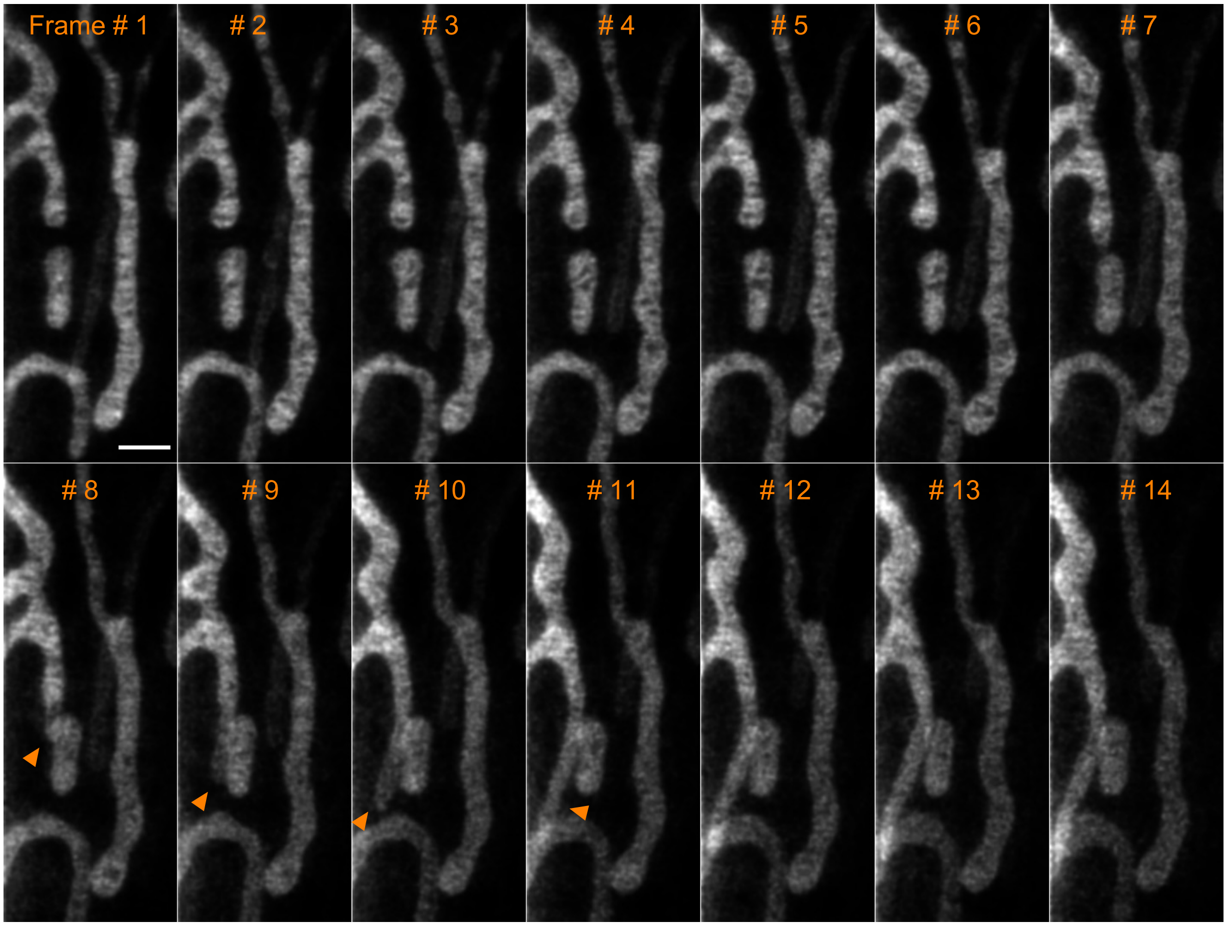

Figure S20. Time-lapse STED nanoscopy of brown adipocytes. Time-lapse STED recording of primary brown adipocytes labeled with PKMO (250 nM, 15 min). Orange triangles indicate an event of mitochondrial tubulation. Scale bar = 1 μm.

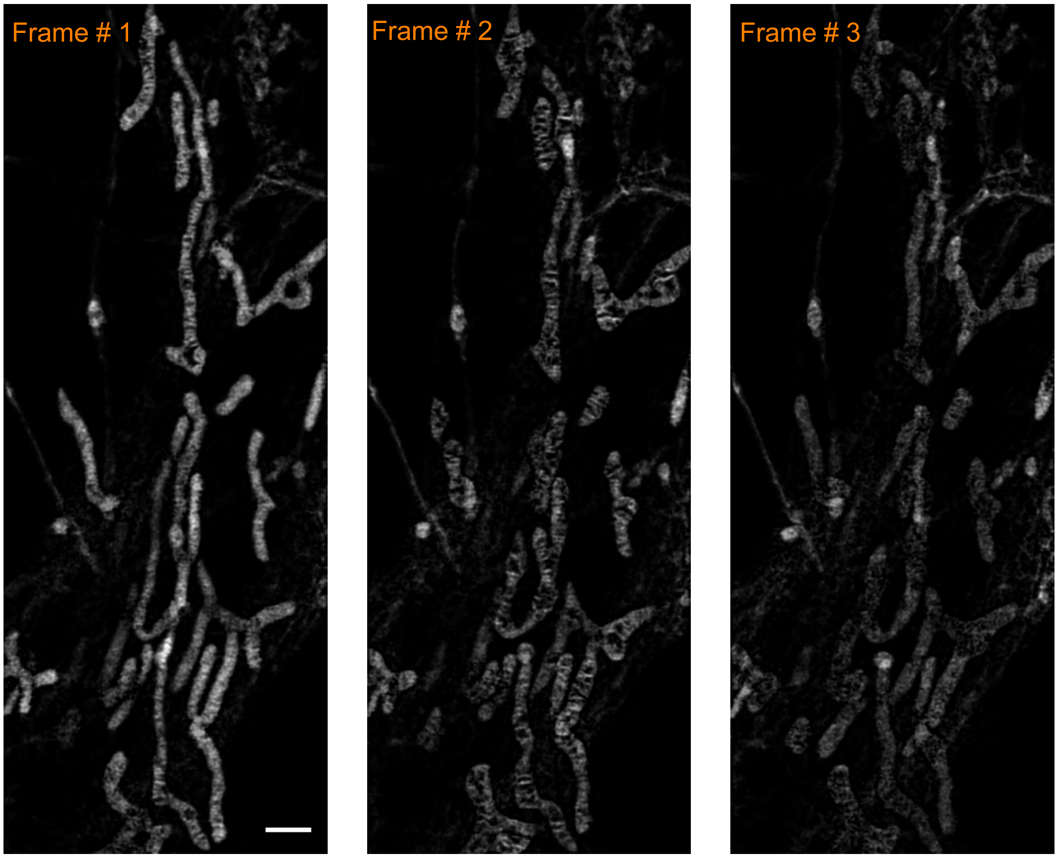

Figure S21. Time-lapse STED nanoscopy of neurons. Time-lapse STED recordings of primary mouse hippocampal neurons labeled with PKMO (250 nM, 15 min). Images show photobleaching and structural changes caused by phototoxic effects. Scale bar = 1 µm.

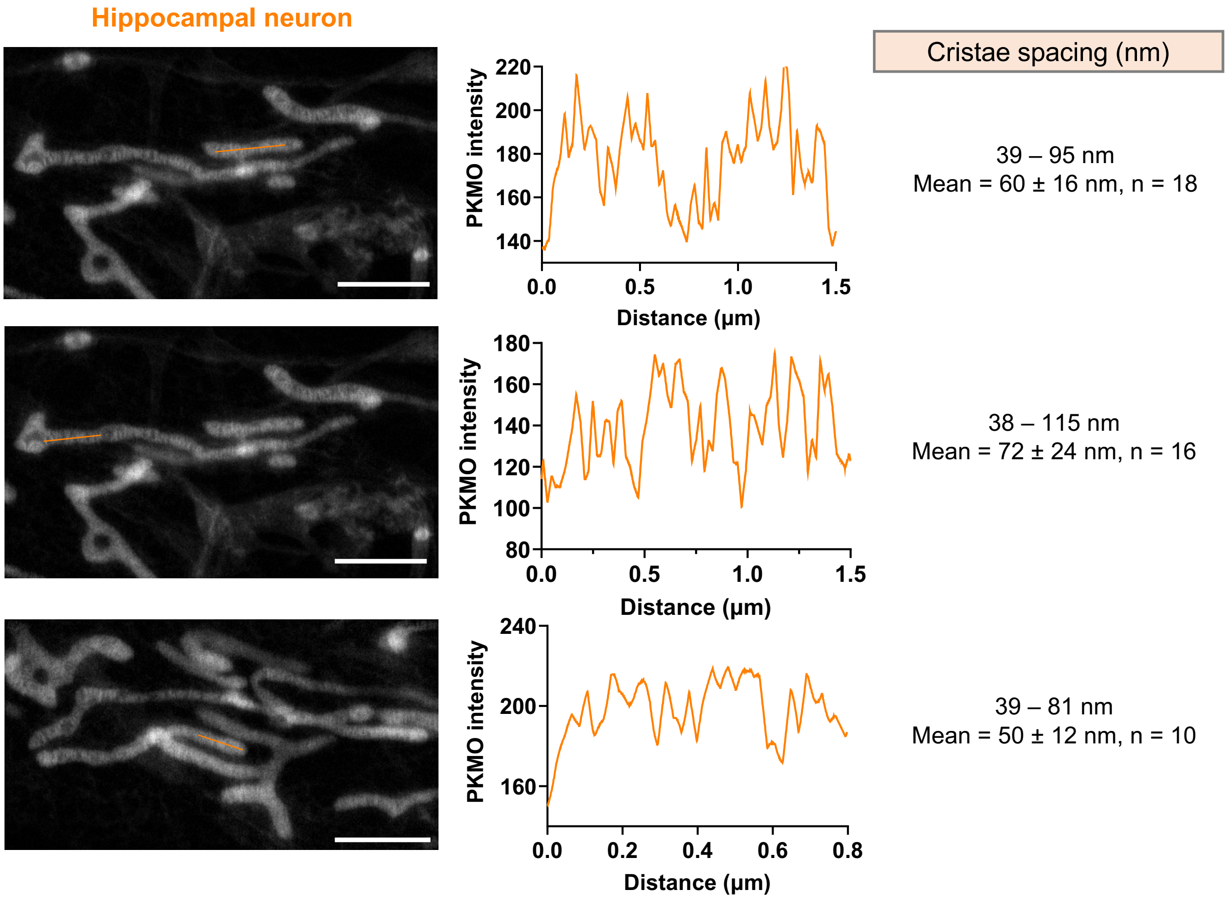

Figure S22. Cristae distance in neurons. STED images of mitochondrial cristae in live primary hippocampal neurons labeled with PKMO (left) and fluorescence intensity line profiles (right). The fluorescence intensity line profiles were measured as indicated by the orange lines in the STED images and used to estimate the cristae distances. Scale bars = 2 µm.

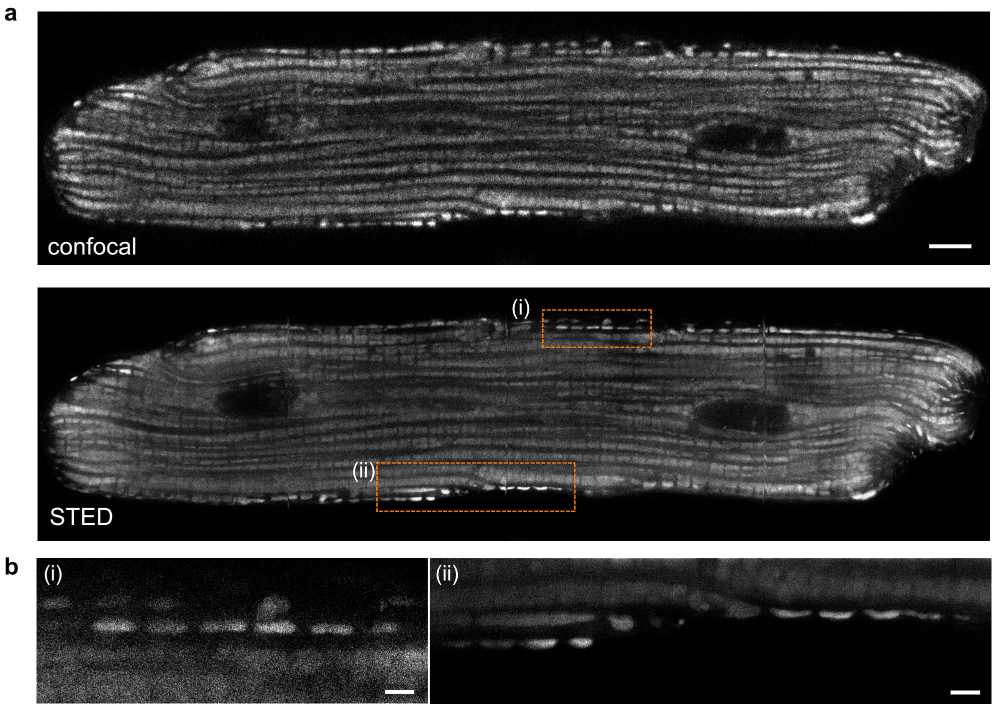

Figure S23. PKMO labeling in rat cardiomyocytes. (a) Confocal and STED images of mitochondria in live primary rat cardiomyocytes labeled with PKMO (500 nM, 15 min). Scale bar = 5 µm. (b) Magnifications of the area indicated in the STED overview images in (a). Scale bars = 1 µm.

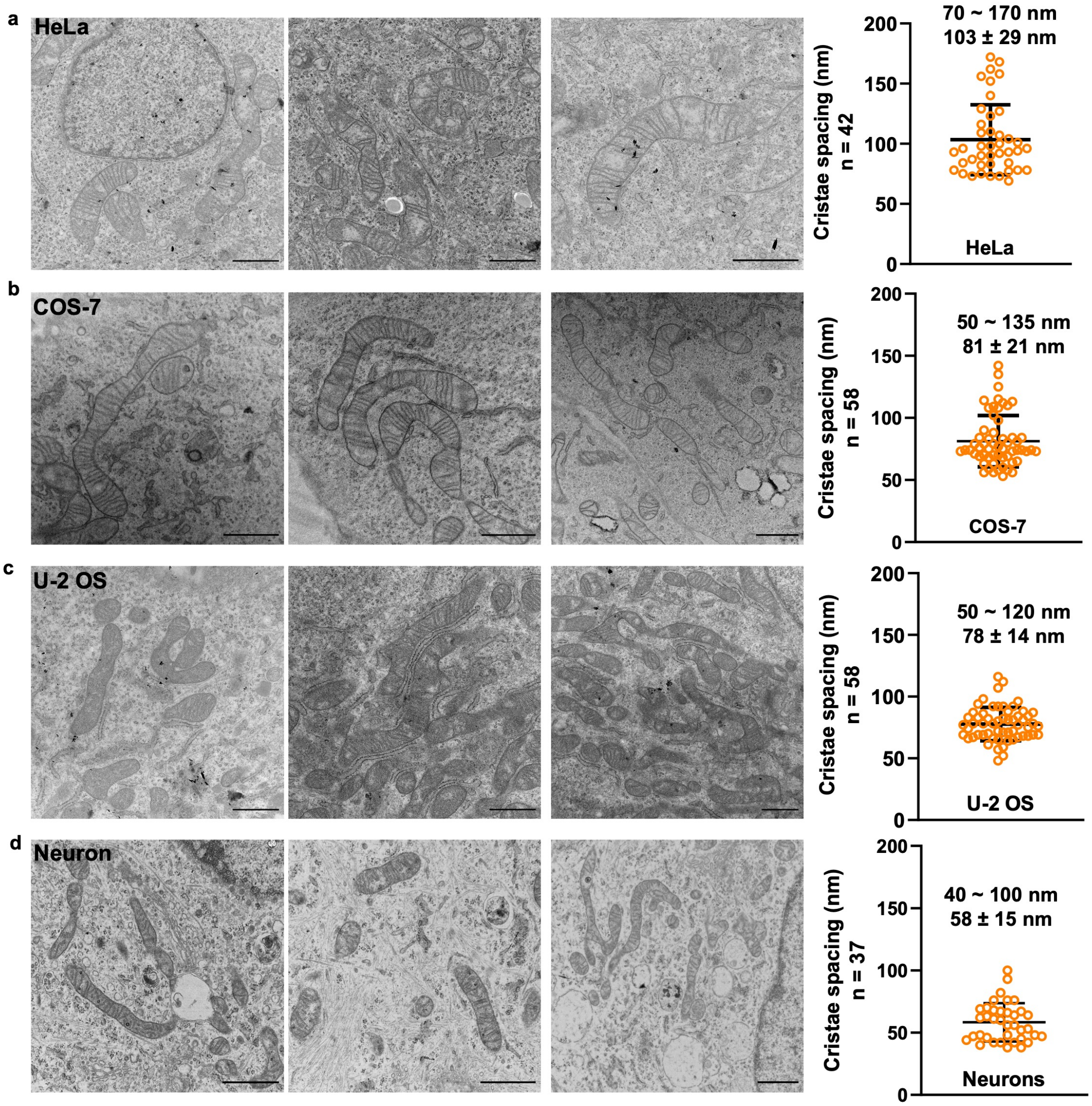

Figure S24. Transmission electron microscopy of cristae. (a-d) TEM recording of HeLa cells (a), COS-7 cells (b), U-2 OS (c) and primary mouse neurons (d) and the corresponding analysis of cristae spacing. Data were analyzed from at least 30 adjacent cristae of three TEM images. The centered line indicates the mean, error bars indicate standard deviation. Scale bars = 1 μm.

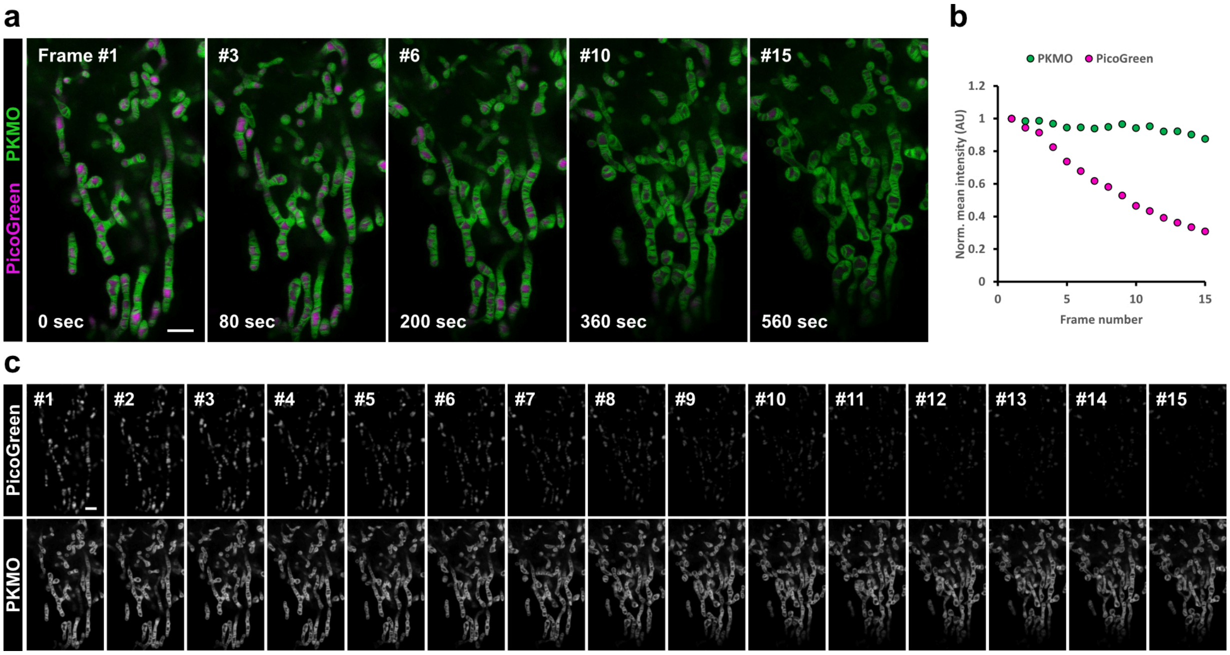

Figure S25. Photobleaching of PKMO and PicoGreen. (a-c) 2D dual-color time-lapse recording of mitochondrial cristae (STED) and DNA (confocal). (a) Selected frames of the time-lapse recording presented in Movie S3 and Figure 5b. Images illustrate bleaching and changes of mitochondrial morphology over time. (b) Photobleaching during dual-color time-lapse images based on the frames presented in (c). Images are raw data without contrast enhancement. Scale bars = 2 µm.

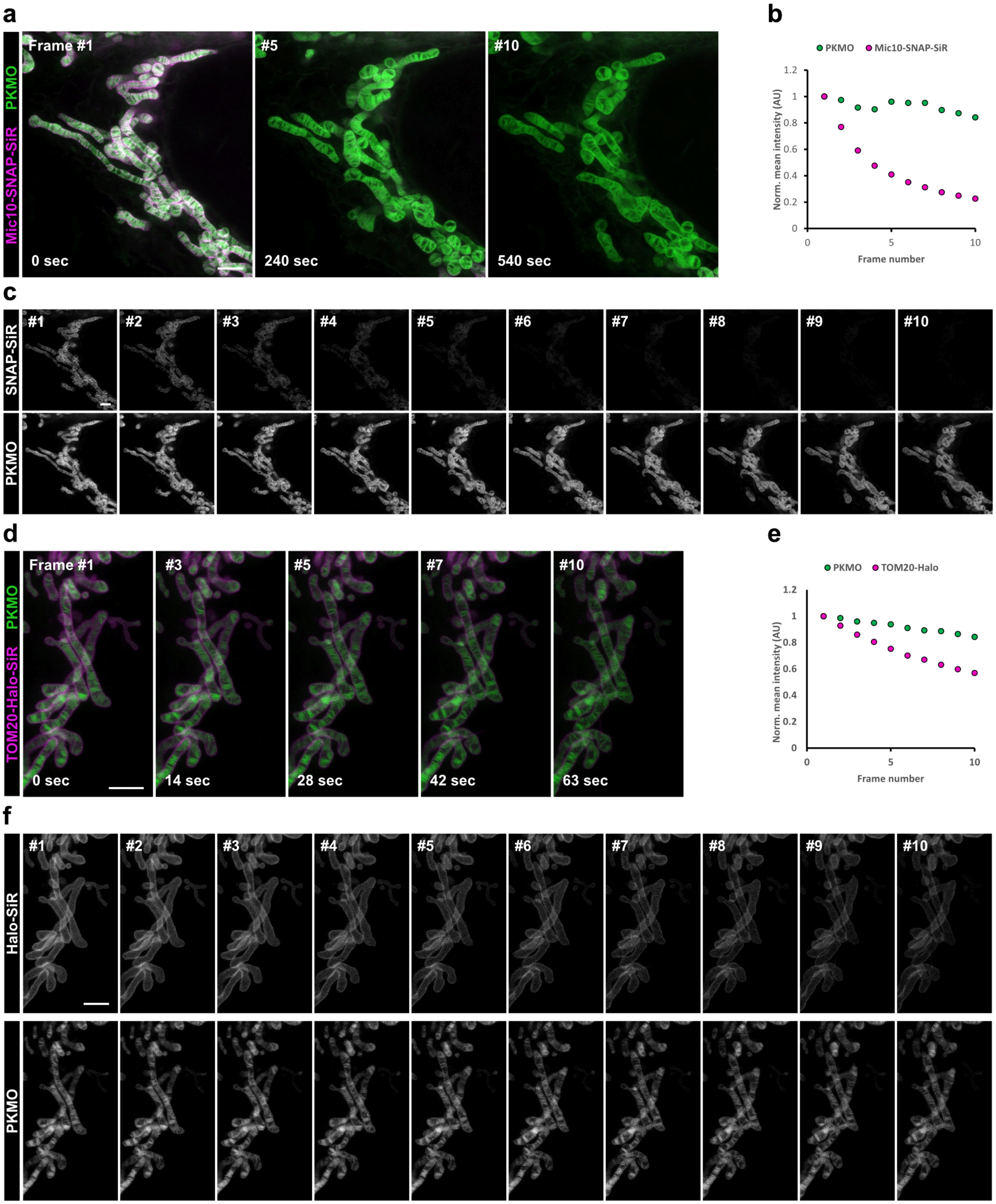

Figure S26. Photobleaching of PKMO and MIC10-SNAP-SiR or TOM20-Halo-SiR. (a-c) 2D dual-color time-lapse STED recording of mitochondrial cristae (PKMO) and MIC10-SNAP (SNAP-Cell-647-SiR). (a) Selected frames of the time-lapse recording presented in Figure 5e. Images illustrate bleaching and changes of mitochondrial morphology over time. (b) Photobleaching during dual-color time-lapse imaging based on the frames presented in (c). (d-f) 2D dual-color time-lapse STED images of mitochondrial cristae (PKMO) and TOM20-Halo (647-SiR-CA). (d) Selected frames of the time-lapse recording presented in Figure 5c. Images illustrate bleaching and changes of mitochondrial morphology over time. (e) Photobleaching during dual-color time-lapse imaging based on the frames presented in (f). All images are raw data without contrast enhancement. Scale bars = 2 µm.

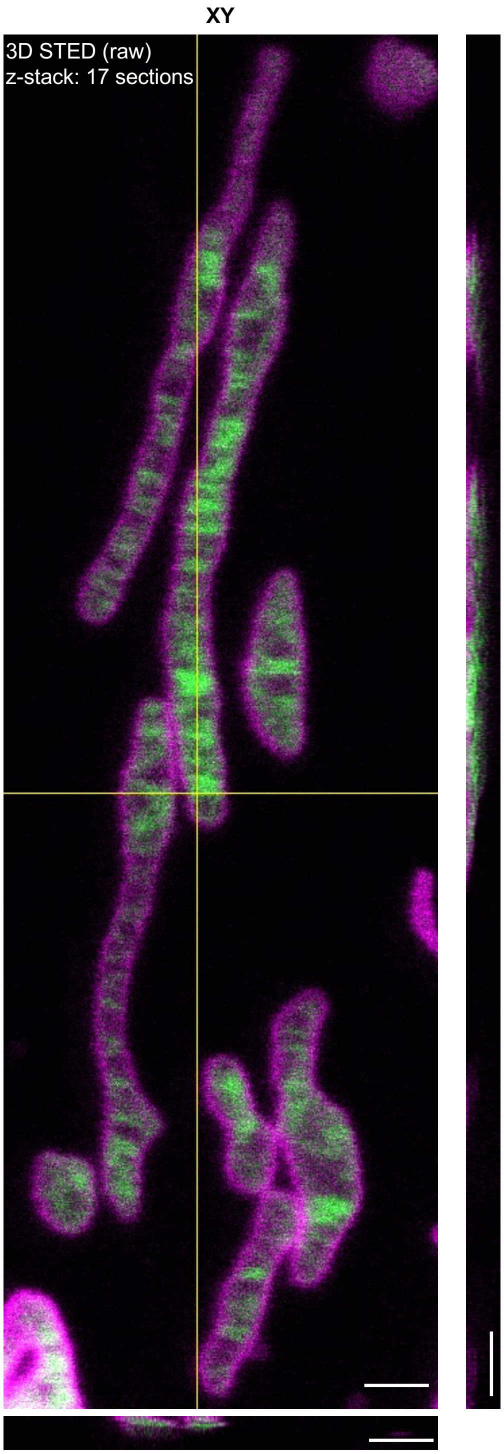

Figure S27. Dual-color 3D STED nanoscopy of mitochondria. Orthogonal view of the dual-color 3D-STED recording of mitochondrial cristae (PKMO, green) and outer membrane (TOM20-Halo, magenta) presented in Fig. 5d. Voxel size= 20 x 20 x 30 nm (XYZ). Image shows raw data. Scale bars = 1 µm.

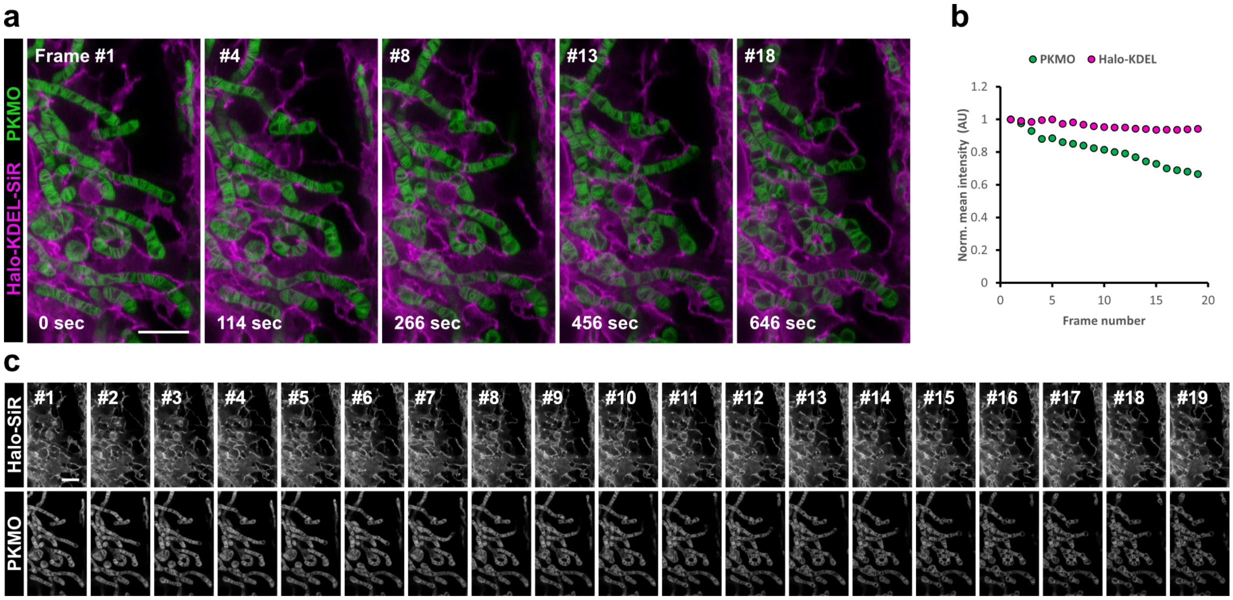

Figure S28. Photobleaching of PKMO and Halo-KDEL-SiR in HeLa cells. (a-c) 2D dual-color time-lapse STED recording of mitochondrial cristae (PKMO) and Halo-KDEL (647-SiR-CA). (a) Selected frames of the time-lapse recording presented in Figure 5e and Movie S4. Images illustrate bleaching and changes of mitochondrial morphology over time. (b) Photobleaching during dual-color time-lapse imaging based on the frames presented in (c). Images show raw data without contrast enhancement. Scale bars = 2 µm.

Table S1. Cristae spacing of various cell lines measured using STED and TEM images.

Cells	Cristae spacing range (nm)		Mean ± SD of cristae spacing (nm)	
	STED	EM	STED	EM
HeLa	90 – 150	70 - 170	127 ± 33<br>n = 20	103 ± 29<br>n = 42
COS-7	50 – 125	50 - 135	90 ± 24<br>n = 20	81 ± 20<br>n = 58
U-2 OS	50 - 125	50 – 120	89 ± 22<br>n = 7	78 ± 14<br>n = 58
Primary hippocampal neurons	38 - 115	40 – 100	62 ± 20<br>n = 44	58 ± 15<br>n = 37

Table S2. Typical staining conditions for the cells used in this work.

Cell lines		Staining condition
**Immortal cells**		
COS-7	PKMO	250 nM, DMEM, 15 min
HeLa	PKMO	150-350 nM, DMEM, 35-45 min
	SNAP-cell SiR	500-1000 nM, DMEM, 40-60 min
	647-SiR-CA	250-500 nM, DMEM, 45 min
	4-610CP-CTX	200-500 nM, DMEM, 40 min
	PicoGreen	1:5000, DMEM, 30- 40 min
	Rho123	300-1000 nM, DMEM, 20-60 min
U-2 OS	PKMO	200-250 nM, McCoy´s, 15 min
**Primary cells**		
Adipocytes	PKMO	250 nM in DMEM, 15 min
Islet cells	PKMO	500 nM in KRBB, 20 min
Neurons	PKMO	250 nM in Neurobasal, 20 min

Table S3. Fluorescence (confocal and STED) microscopy data acquisition parameters.

Figure	Microscope	Labels	Imaging mode	Laser settings	Objective	Detection (nm)	Pixel dwell time [µs]	Line accumulations	Pixel/voxel Size [nm]	FOV [µm]	Number of frames	Frame interval [s]	Staining conditions
2a	Facility line	PKMO	2D STED	λ_{EX}561nm (20%)<br>λ_{STED}775nm (20%)	60x	571-681	10	5	25	58.15 x 50.45			250 nM<br>15min<br>DMEM
2d	STEDYCON	PKMO	2D STED	λ_{EX}561nm (5%)<br>λ_{STED}775 (75%)	100x	571-681	10	5	30	6.9 x 15.51	33	15.4	250 nM<br>15min<br>DMEM
2d	STEDYCON	PKMO 0.9	2D STED	λ_{EX}561nm (10%)<br>λ_{STED}775 (75%)	100x	571-681	10	5	30	8.4 x 21.99	15	9.6	250 nM<br>15min<br>DMEM
2e	Facility line	PKMO	2D STED	λ_{EX}561nm (10%)<br>λ_{STED}775nm (20%)	60x	571-681	10	5	30	11.52 x 24.72	23	19.5	250 nM<br>15min<br>DMEM
2f	Facility line	PKMO	2D STED	λ_{EX}561nm (10%)<br>λ_{STED}775nm (20%)	60x	571-681	10	5	30	11.52 x 24.72	23	19.5	250 nM<br>15min<br>DMEM
3	Facility line	PKMO	3D STED	λ_{EX}561nm (10%)<br>λ_{STED}775nm (30%)	60x	571-681	10	2	20<br>x20<br>x50	4.1 x 14.74 x 0.95	19<br>z stack	3.1	250 nM<br>15min<br>DMEM
4a	Expert Line	PKMO	2D STED	λ_{EX}561 nm (15%, ~1.3 µW)<br>λ_{STED}775 nm (22%, ~43 mW)	100x	580-630	4	9	30	76 x 71.4	1		200 nM<br>40 min<br>DMEM
4b	Expert Line	PKMO	2D STED	λ_{EX}561 nm (14%, ~1.2 µW)<br>λ_{STED}775 nm (20%, ~39 mW)	100x	580-630	5	8	25	25.6 x 24.2	5	81.1	250 nM<br>20 min<br>McCoy
4c	Facility line	PKMO	Conf.	λ_{EX}561nm (1%)	60x	571-681	2	1	166.7	125 x 125			250 nM<br>15 min<br>DMEM
4c<br>(i)<br>(ii)	Facility line	PKMO	2D STED	λ_{EX}561nm (15%)<br>λ_{STED} 775 nm (20%)	60x	571-681	10	3	30	25.08 x 50.01			250 nM<br>15 min<br>DMEM
4c<br>(iii)	Facility line	PKMO	3D STED	λ_{EX} 561 nm (15%)<br>λ_{STED} 775 nm (20%)	60x	571-681	10	3	30	11.76 x 22.74	20	11.1	250 nM<br>15 min<br>DMEM
4d	Facility line	PKMO	Conf.	λ_{EX} 561 nm (1%)	60x	571-681	2	1	100	125 x 125			250 nM<br>20 min<br>Neurobasal
4d	Facility line	PKMO	STED	λ_{EX} 561 nm (20%)<br>λ_{STED} 775 nm (35%)	60x	571-681	10	3	20	7.12 x 26.82			250 nM<br>20 min<br>Neurobasal
4e	Facility line	PKMO	Conf.	λ_{EX} 561 nm (2%)<br>λ_{EX} 488 nm (7%)	60x	577-687<br><br>498-551	10	1	100	132.8 x 121.2			500 nM<br>20 min<br>KRBB
4e	Facility line	PKMO	2D STED	λ_{EX} 561 nm (10%)<br>λ_{STED} 775 nm (20%)	60x	577-687	10	3	20	8.1 x 17.26			500 nM<br>20 min<br>KRBB
5b	Expert line	PicoGreen<br><br>PKMO	Conf.<br><br>2D STED	λ_{EX} 485 nm (2%, ~1.9 µW)<br><br>λ_{EX} 561 nm (15%, ~1.3 µW)<br>λ_{STED} 775 nm (22%, ~ 43 mW)	100x	500-550<br><br>580-630	5	3 (PicoGreen)<br><br>9 (PKMO)	25	13.6 x 23.2	15	39.5	PKMO:<br>150 nM, 45 min, DMEM<br><br>Afterward:<br><br>PicoGreen:<br>1:5000, 30 min, DMEM
5c-5d	Facility line	PKMO<br>SiR-CA	3D STED	λ_{EX} 561 nm (30%)<br>λ_{STED} 775 nm (30%)<br><br>λ_{EX} 640 nm (2%)<br>λ_{STED} 775 nm (10%)	60x	571-630<br>(PKMO)<br><br>650-775<br>(SiR)	10	1	20x<br>30x<br>30	10.88 x 28.82 x 1.14	37<br>z stack	15.7	250 nM<br>15min<br>DMEM<br><br>SiR:<br>500nM, 10 min, DMEM
5e	Expert line	PKMO<br><br>SNAP-cell SiR	2D STED	λ_{EX} 561 nm (12%, ~1.1 µW)<br>λ_{STED} 775 nm (20%, ~39 mW)<br><br>λ_{EX} 640 nm (4%, ~ 9 µW)<br>λ_{STED} 775 nm (27%, ~53 mW)	100x	580-630<br>(PKMO)<br><br>650-720<br>(SiR)	5	9 (PKMO)<br><br>9(SiR)	25	24.8 x 29	10	58.8	PKMO:<br>200 nM, 40 min, DMEM<br><br>Afterward:<br><br>1 µM SNAP-cell SiR: 60 min, DMEM
5f	Expert line	PicoGreen<br><br>PKMO<br><br>4-610CP-CTX	2D STED<br>+<br>Conf.	λ_{EX} 485 nm (0.6%, ~0.5 µW)<br><br>λ_{EX} 561 nm (16%, ~1.9 µW)<br>λ_{STED} 775 nm (22%, ~ 43 mW)<br><br>λ_{EX} 640 nm (4%, ~ 9 µW)<br>λ_{STED} 775 nm (27%, ~53 mW)	100x	500-550<br>(PicoGreen)<br><br>580-630<br>(PKMO)<br><br>650-720<br>(610CP)	7	2 (PicoGreen)<br><br>8 (PKMO)<br><br>6 (610CP)	28	18.2 x 25.6	1		PKMO: 350 nM<br>4-610CP-CTX: 200 nM<br>PicoGreen: 1:5000,<br>40 min, DMEM
5g	Expert line	PKMO<br><br>647-SiR-CA	2D STED	λ_{EX} 561 nm (4%, ~ 0.35 µW)<br>λ_{STED} 775 nm (23%, ~45 mW)<br>λ_{EX} 640 nm (1%, ~ 2.3 µW)<br>λ_{STED} 775 nm (30%, ~59 mW)	100x	580-630<br>(PKMO)<br>650-720<br>(SiR)	7	6 (PKMO)<br><br>2 (SiR)	25	24.8 x 15	20	37.3	PKMO: 300 nM<br><br>647-SiR-CA: 0.5 µM<br>45 min, DMEM

6	Expert line	mEGFP<br><br>PKMO	2D STED<br>+<br>Conf.	λ_{Ex}485 (0.5%, ~ 0.5 µW)<br><br>λ_{Ex}561 (2.2%, ~ 0.2 µW)<br>λ_{STED}775nm (20%, ~ 39 mW)	100x	500-550 (mEGFP)<br><br>580-630 (PKMO)	8	1 (mEGFP)<br><br>8 (PKMO)	30	35.3 x 43	11	240	250 nM, 45 min, DMEM
7a	Expert line	PicoGreen<br><br>PKMO	2D STED<br>+<br>Conf.	λ_{Ex} 485 nm (2%, ~1.9 µW)<br><br>λ_{Ex} 561 nm (16%, ~1.4 µW)<br>λ_{STED} 775 nm (22%, ~43 mW)	100x	500-550 (PicoGreen)<br><br>580-630 (PKMO)	4	3 (PicoGreen)<br><br>9 (PKMO)	25	39 x 34.4	1		PKMO: 150 nM, 45 min, DMEM<br><br>Afterward:<br><br>PicoGreen: 1:5000, 30 min, DMEM
7b	Expert line	PicoGreen<br><br>PKMO	2D STED<br>+<br>Conf.	λ_{Ex}485 (1%, 0.9 µW)<br><br>λ_{Ex}561 (7%, 0.6 µW)<br>λ_{STED}775nm (20%, 39 mW)	100x	500-550 (PicoGreen)<br><br>580-630 (PKMO)	4	3 (PicoGreen)<br><br>12 (PKMO)	30	37.8 x 28.2	1		KMO: 150 nM, 45 min, DMEM<br><br>Afterward:<br><br>PicoGreen: 1:5000, 30 min, DMEM
7c	Expert line	PicoGreen<br><br>PKMO	2D STED<br>+<br>Conf.	λ_{Ex}485 (1%, ~0.9 µW)<br><br>λ_{Ex}561 (7%, ~ 0.6 µW)<br>λ_{STED}775nm (20%, ~ 39 mW)	100x	500-550 (PicoGreen)<br><br>580-630 (PKMO)	4	3 (PicoGreen)<br><br>12 (PKMO	30	26.8 x 29.4	1		PKMO: 150 nM, 45 min, DMEM<br><br>Afterward:<br><br>PicoGreen: 1:5000, 30 min, DMEM
S11a	STEDYCON	Rho123<br><br>PKMO	2D STED<br>+<br>Conf.	λ_{Ex}488 (10%)<br><br>λ_{Ex}561 (10%)<br>λ_{STED}775nm (75%)	100x	500-550 (Rho123)<br><br>571-681 (PKMO)	10	2	30	6 x 11	20	4.7	PKMO: 250 nM, 60min, DMEM<br><br>Rho123: 300 nM, 60min, DMEM
S11b	STEDYCON	Rho123<br><br>PKMO 0.9	2D STED<br>+<br>Conf.	λ_{Ex}488 (10%)<br><br>λ_{Ex}561 (4.4%)<br>λ_{STED}775nm (75%)	100x	500-550 (Rho123)<br><br>571-681 (PKMO)	10	2	30	6 x 13	10	5.6	PKMO 0.9: 250 nM, 60min, DMEM<br><br>Rho123: 300 nM, 60min, DMEM
S11c	STEDYCON	Rho123	2D STED<br>+<br>Conf.	λ_{Ex}488 (10%)<br><br>λ_{Ex}561 (4.4%)<br>λ_{STED}775nm (75%)	100x	500-550 (Rho123)<br><br>571-681 (PKMO)	10	2	30	7 x 12	10	6.1	Rho123: 300 nM, 60min, DMEM
S13a+b	Expert line	Rho123<br><br>PKMO	2D STED<br>+<br>Conf	λ_{Ex}485 (2%, ~1.9 µW)<br><br>λ_{Ex}561 (8%, ~ 0.7 µW)<br>λ_{STED}775nm (22%, ~ 43 mW)	100x	500-550 (Rho123)<br><br>580-630 (PKMO)	7	3 (Rho123)<br><br>9 (PKMO)	30	10 x 10	20	15.85	PKMO: 250 nM, 40 min, DMEM<br><br>Afterward:<br><br>Rho123: 1 µM, 20 min, DMEM
S13c	Expert line	Rho123<br><br>SNAP-cell SiR	2D STED<br>+<br>Conf	λ_{Ex}485 (1%, ~ 0.9 µW)<br><br>λ_{Ex}640 (3%, ~ 6.8 µW)<br>λ_{STED}775nm (28%, ~ 55 mW)	100x	500-550 (Rho123)<br><br>650-720 (SiR)	7	3 (Rho123)<br><br>9 (SiR)	30	10 x 10	20	15.85	SNAP-cell SiR: 1 µM, 40 min, DMEM<br><br>Afterward:<br><br>Rho123: 1 µM, 20 min, DMEM
S15	Expert line	PKMO	2D STED	λ_{Ex}561 (6%, ~ 0.6 µW)<br>λ_{STED}775nm (20%, ~39 mW)	100x	580-630 (PKMO)	7	12	28	14.8 x 12	20	23.3	250 nM, 40 min, DMEM

Chemical Synthesis and Characterization of New Compounds

General Information

Unless otherwise mentioned, all reactions were carried out under a nitrogen atmosphere with dry solvents under anhydrous conditions. All the chemicals were purchased at the highest commercial quality and used without further purification unless otherwise stated. Reactions were monitored by Thin Layer Chromatography on plates (GF254) supplied by Yantai Chemicals (Yantai, Shandong, China) using UV light as a visualizing agent and an ethanolic solution of phosphomolybdic acid and cerium sulfate, and heat as developing agents or by LC/MS (4.6 mm × 150 mm 5 μm C18 column; 5 μL injection; 10-95% or 50-95% CH_3CN/H_2O, linear-gradient, with constant 0.1% v/v TFA additive; 20 min run; 1 mL/min flow; ESI; positive ion mode; UV detection at 254 nm). Flash column chromatography uses silica gel (200-300 mesh) supplied by Tsingtao Haiyang Chemicals (Qingdao, Shandong, China). NMR spectra were recorded on Brüker Advance 400 (^{1}H 400 MHz, ^{13}C 101 MHz) and are calibrated using residual undeuterated solvent ($CDCl_3$ at 7.26 ppm ^{1}H NMR, 77.16 ppm ^{13}C NMR; CD_3OD at 3.31 ppm ^{1}H NMR, 49.00 ppm ^{13}C NMR; DMSO-d6 at 2.50 ppm ^{1}H NMR, 39.52 ppm ^{13}C NMR). Data for ^{1}H NMR spectra are reported as follows: chemical shift (δ ppm), multiplicity (s = singlet, d = doublet, t = triplet, q = quartet, dd = doublet of doublets, dt = triplet of doublets, m = multiplet, br = broad), coupling constant (Hz), integration. Data for ^{13}C NMR are reported by chemical shift (δ ppm).

Synthetic Procedure and Characterization

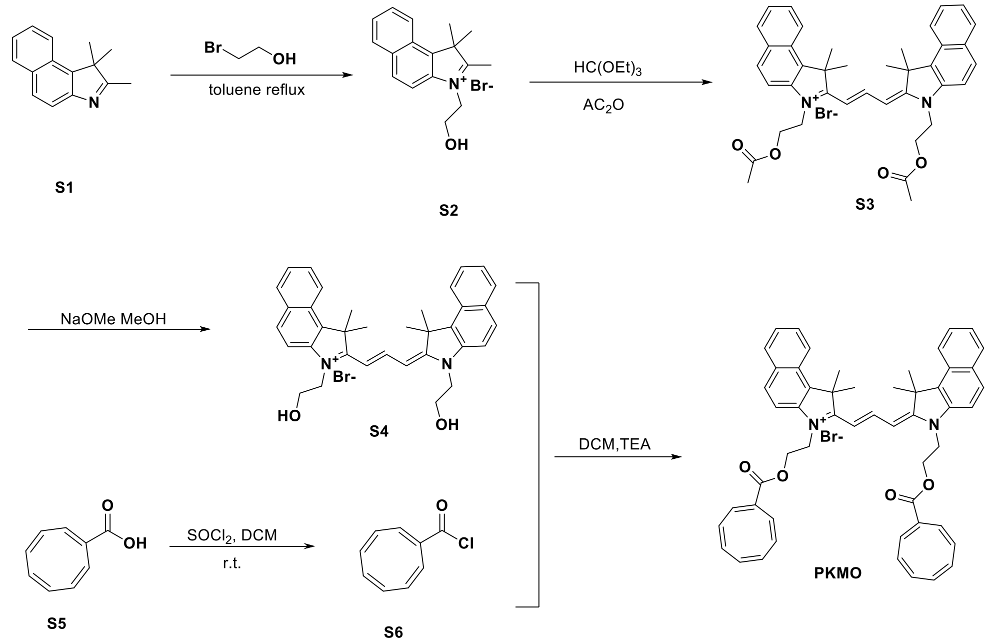

Scheme S1. Synthetic route of Compound PKMO and PKMO 0.9

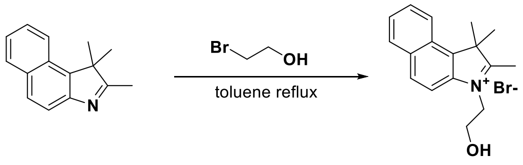

S1 S2

Compound S2: To a solution of compound S1 (500 mg, 2.39 mmol) in toluene (5 mL) was added 2-Bromoethan-1-ol (CAS: 540-51-2) (896 mg, 7.17 mmol) at room temperature (r.t.), the mixture was heated at 110°C and stirred for 16 h. The reaction mixture was then cooled to r.t slowly and a solid precipitated. The solid was collected by filtration, followed by washing with methyl tert-butyl ether (10 mL), the solid was then dried under vacuum to afford the compound (700 mg, 88%) as a brown solid.

^{1}H NMR (400 MHz, Methanol-d4) δ 8.34 (d, J = 8.5 Hz, 1H), 8.23 (d, J = 9.0 Hz, 1H), 8.17 (d, J = 8.3 Hz, 1H), 8.01 (d, J = 9.0 Hz, 1H), 7.81 (td, J = 8.5, 1.4 Hz, 1H), 7.72 (td, J = 8.3, 1.2 Hz, 1H), 4.79(t, J =6.0 Hz, 2H), 4.12 (t, J =6.0 Hz, 2H), 1.86 (s, 6H).

MS (ESI) calcd for $C_{17}H_{20}NO^+$ $[M]^+$ 254.15, found 254.06.

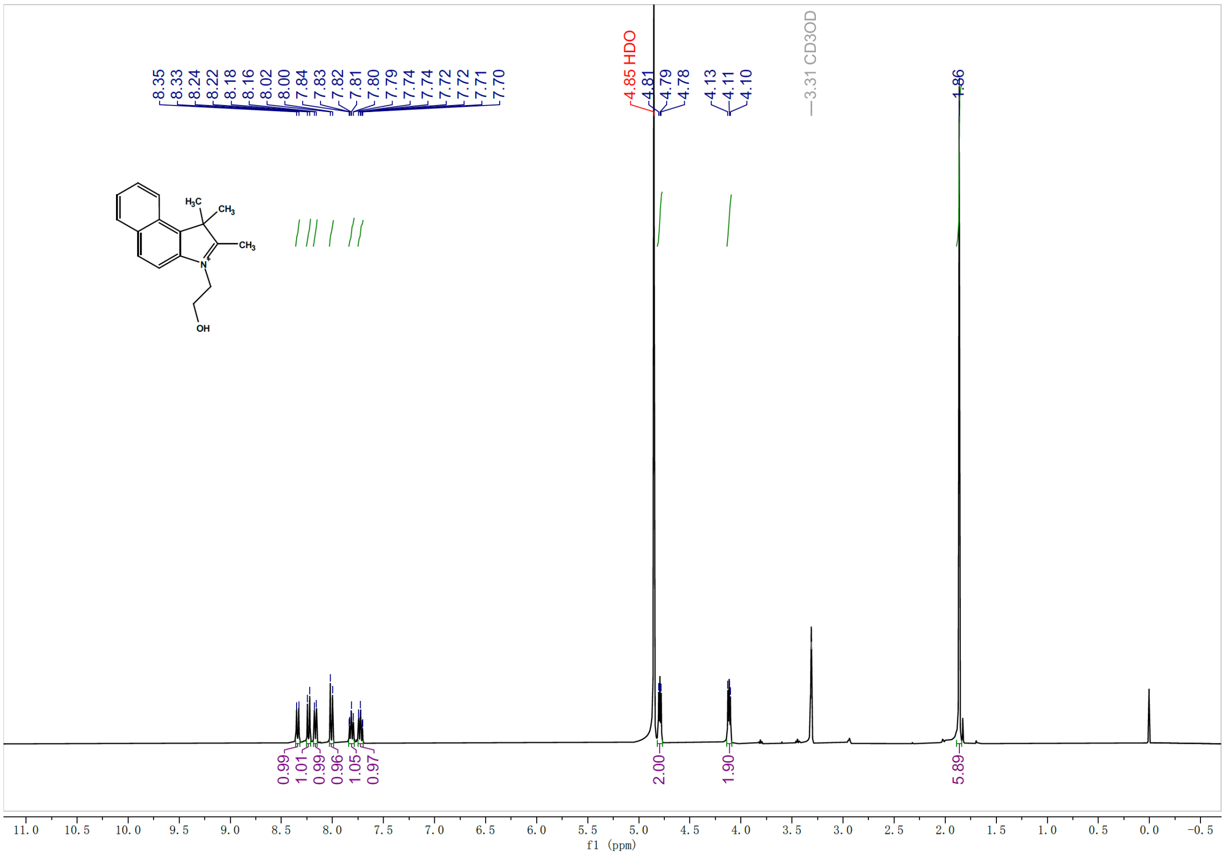

^{1}H NMR spectrum of compound S2

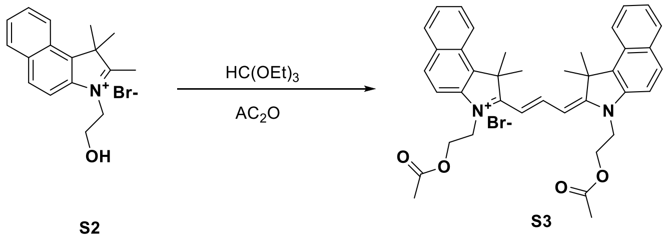

S2 S3

Compound S3: A mixture of compound **S3** (450 mg, 1.35 mmol), triethyl orthoformate (120 mg, 0.808 mmol), and acetic anhydride (4.5 mL) in a pressure tube was heated at 120°C and stirred for 16 h. The mixture was then cooled to r.t. The solvent was removed under reduced pressure to obtain a residue, which was purified by a silica gel chromatography column, eluting with DCM: MeOH=15:1 to afford the compound (330 mg, 72%) as a purple solid.

^{1}H NMR (400 MHz, Chloroform-*d*) δ 8.69 (t, *J* = 13.3 Hz, 1H), 8.11 (d, *J* = 9.2 Hz, 2H), 7.96-=7.92 (m, 4H), 7.74 (d, *J* = 13.3 Hz, 2H), 7.63 (td, *J* = 7.0, 1.3 Hz, 2H), 7.50 (t, *J* = 8.4 Hz, 2H), 7.45 (d, *J* = 8.9 Hz, 2H) 4.82 – 4.69 (m, 8H), 2.05 (s, 12H), 1.77 (s, 6H).

MS (ESI) calcd for $C_{39}H_{41}N_2O_4^+$ [M]$^+$ 601.31, found 601.17.

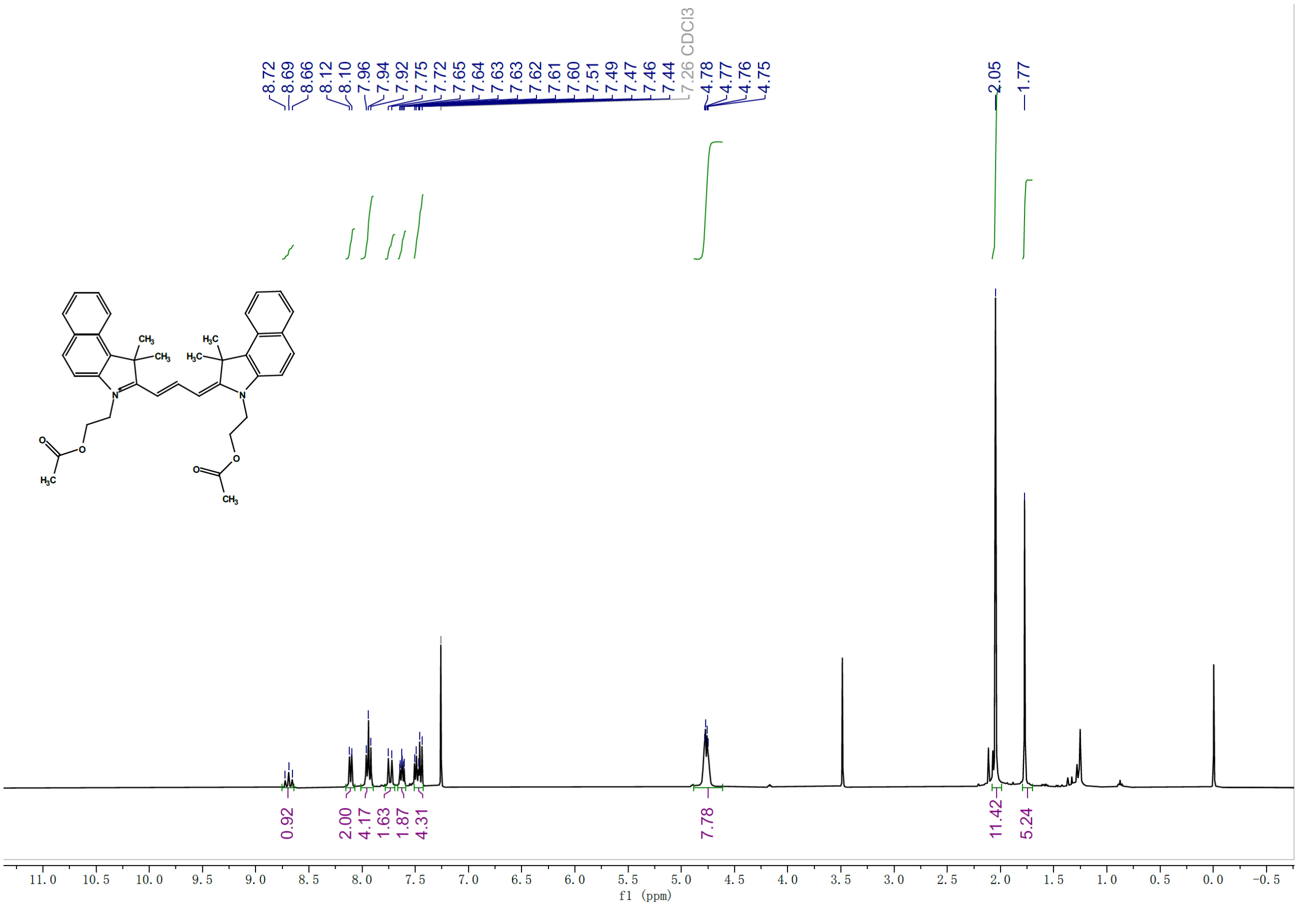

^{1}H NMR spectrum of compound S3

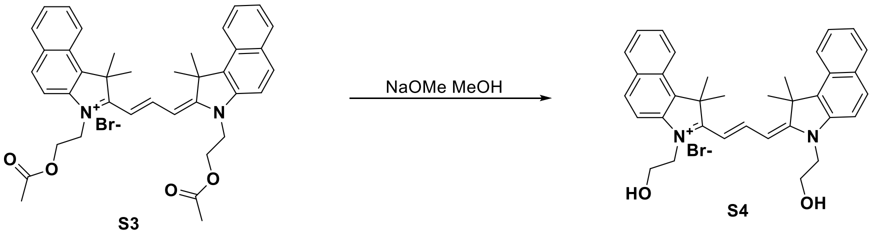

Compound S4: To a mixture of compound **S3** (280 mg, 0.411 mmol) in MeOH (6.0 mL) was added NaOMe (111 mg, 2.06 mmol) at r.t. The mixture was stirred for 3 h before concentrated under reduced pressure to obtain a residue, which was purified by a silica gel chromatography column, eluting with DCM: MeOH=30:1 to 10:1 to afford the compound (210 mg, 86%) as a purple solid.

^{1}H NMR (400 MHz, Methanol-*d*$_4$) δ 8.83 (t, *J* = 13.5 Hz, 1H), 8.29 (d, *J* = 8.5 Hz, 2H), 8.03- 7.99 (m, 4H), 7.69-7.65 (m, 4H), 7.51 (t, *J* = 7.5 Hz, 2H), 6.56 (d, *J* = 13.5 Hz, 2H), 4.42 (t, *J* = 5.3 Hz, 4H), 4.06 (t, *J* = 5.1 Hz, 4H), 2.11 (s, 12H).

^{13}C NMR (101 MHz, Methanol-*d*$_4$) δ 178.31, 150.87, 141.50, 134.71, 133.58, 131.60, 131.13, 129.26, 128.82, 126.25, 123.36, 112.74, 103.66, 60.31, 52.48, 48.08, 28.00.

MS (ESI) calcd for $C_{35}H_{37}N_2O_2^+$ [M]$^+$ 517.28, found 517.16.

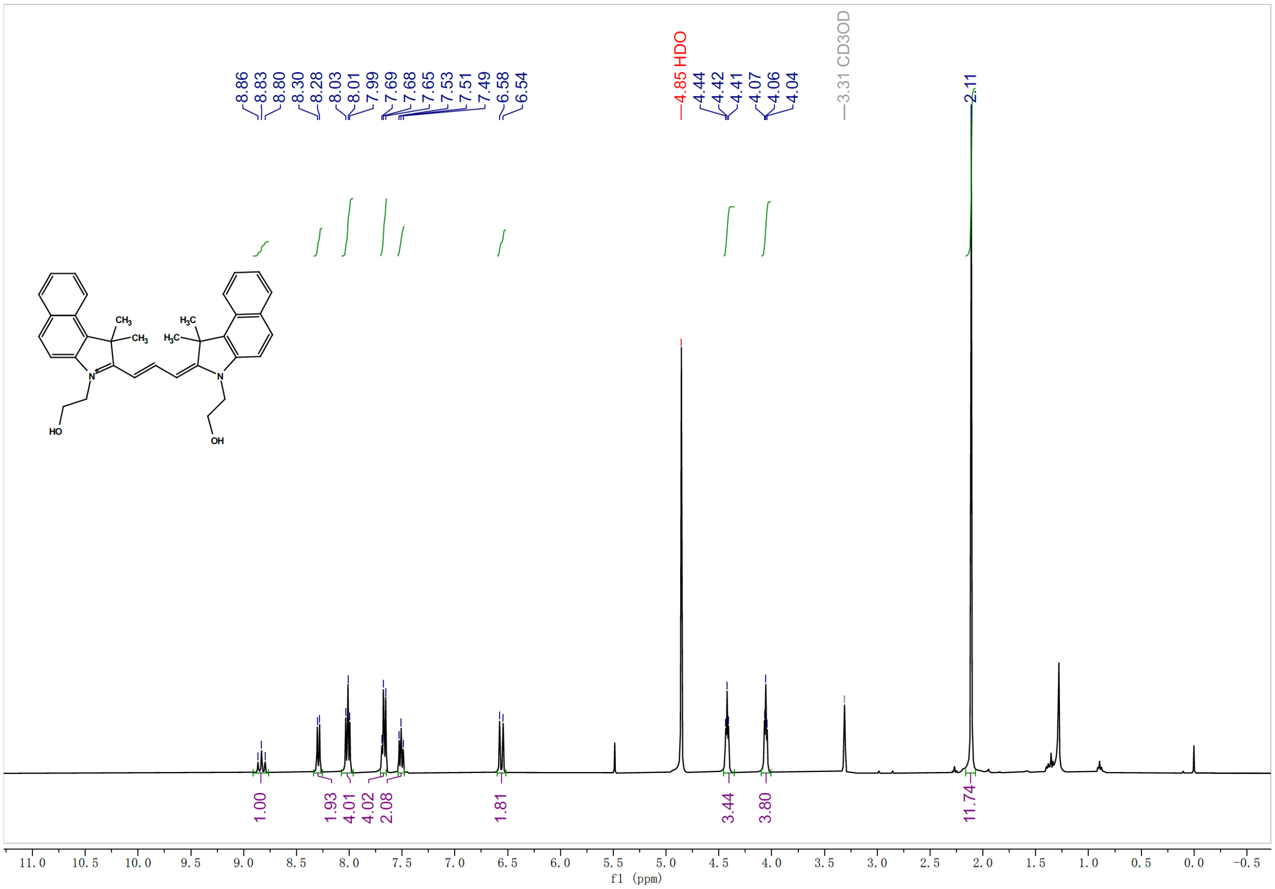

^{1}H NMR spectrum of compound S4

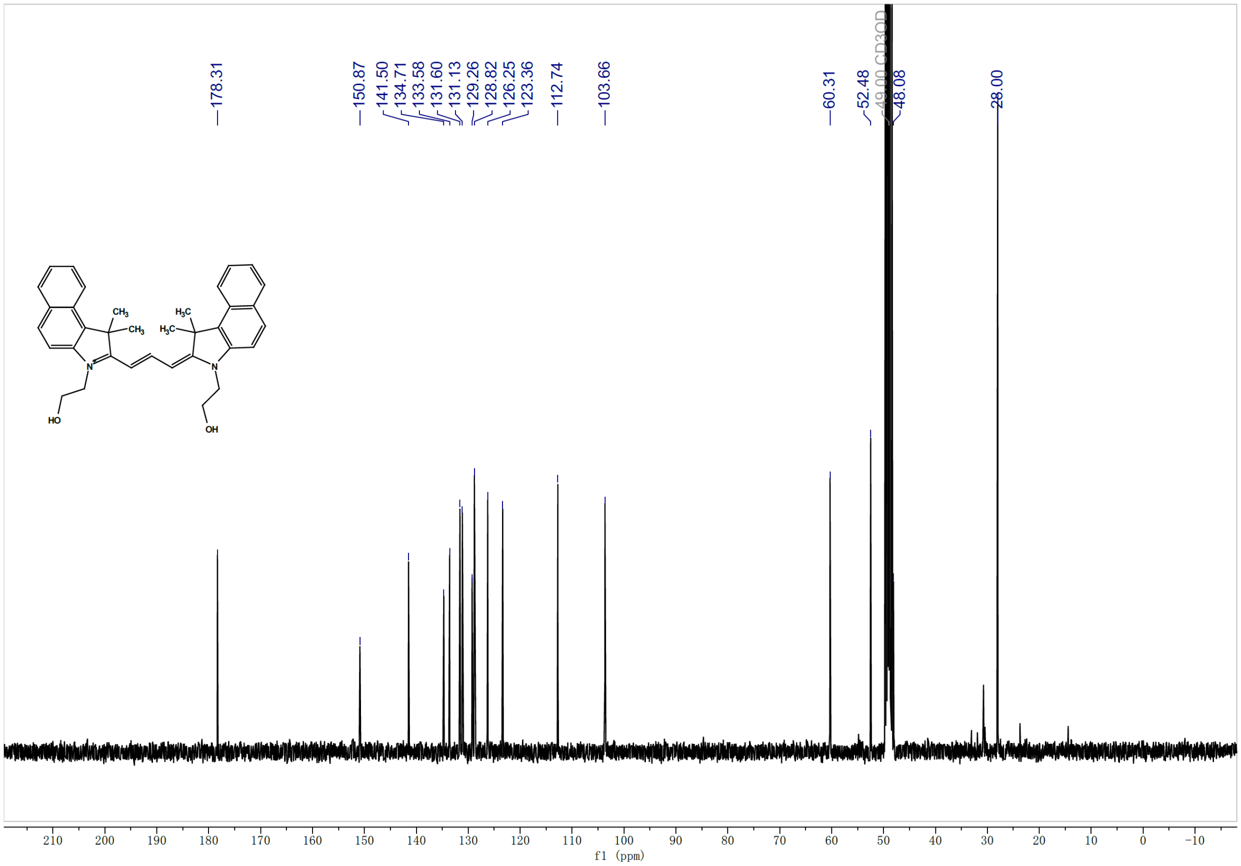

^{13}C NMR spectrum of compound S4

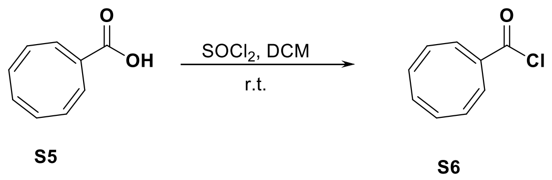

Compound S6: To a solution of compound **S5** (60.0 mg, 0.405 mmol) in DCM (5.0 mL) were added $SOCl_2$ (242 mg, 2.03 mmol) and DMF (10 uL) in turn at r.t under nitrogen. The mixture was then stirred for 2 h at r.t. before concentrated under reduced pressure to obtain the crude **S6**, which was used in the next step directly.

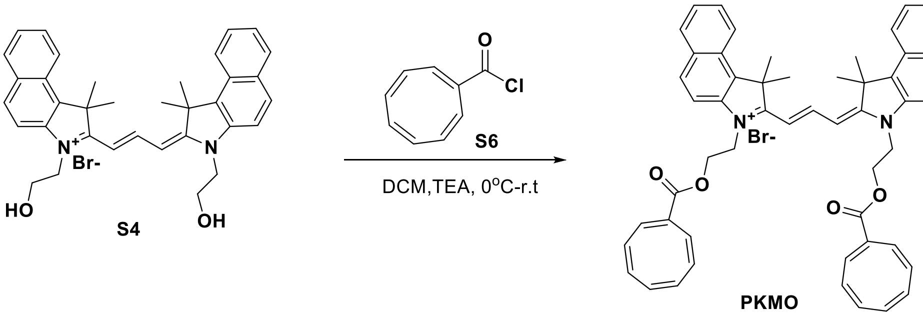

PKMO: To a mixture of compound **S4** (35.0 mg, 0.059 mmol) and triethylamine (59.7 mg, 0.590 mmol) in dry DCM (3.0 mL) was added dropwise the solution of compound **S6** (67.5 mg, 0.405 mmol) in dry DCM (1.0 mL) at 0°C under nitrogen. The mixture was stirred at room temperature for 16 h before concentrated under reduced pressure to afford a residue, which was purified by preparative TLC (DCM: MeOH=15:1) to afford crude **PKMO** (40.0 mg, 79%) as a purple solid. The solid was dissolved in DCM (2.0 mL) and added dropwise into diethyl ether (20.0 mL) slowly. The solid was collected and dried under reduced pressure to afford **PKMO** (25.0 mg, 49%) as a purple solid.

^{1}H NMR (400 MHz, Methanol-*d*$_4$) δ 8.78 (t, *J* = 13.2 Hz, 1H), 8.28 (d, *J* = 8.5 Hz, 2H), 8.04 - 8.01 (m, 4H), 7.79 – 7.61 (m, 4H), 7.52 (t, *J* = 7.5 Hz, 2H), 6.82 (s, 2H), 6.64 (dd, *J* = 13.6, 4.4 Hz, 2H), 5.85 – 5.37 (m, 14H), 4.79 – 4.59 (m, 8H), 2.07 (s, 12H).

^{13}C NMR (101 MHz, Methanol-*d*$_4$) δ 178.13, 166.54, 151.10, 144.86, 140.99, 135.25, 134.71, 134.15, 133.77, 133.66, 132.85, 132.41, 131.91, 131.16, 130.78, 130.01, 129.20, 128.90, 126.39, 123.36, 112.54, 103.97, 62.03, 52.48, 44.45, 28.03.

MS (ESI) calcd for $C_{53}H_{49}N_2O_4^+$ [M]$^+$ 777.37, found 777.28.

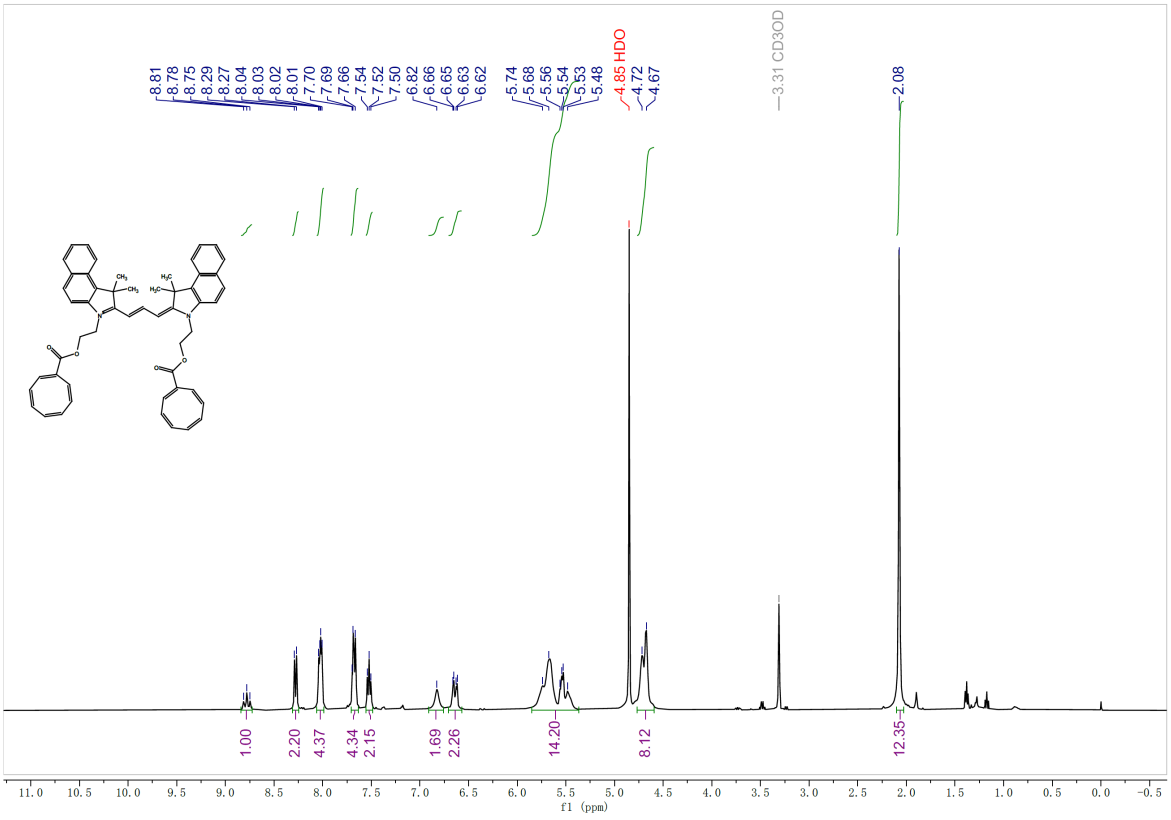

^{1}H NMR spectrum of compound PKMO

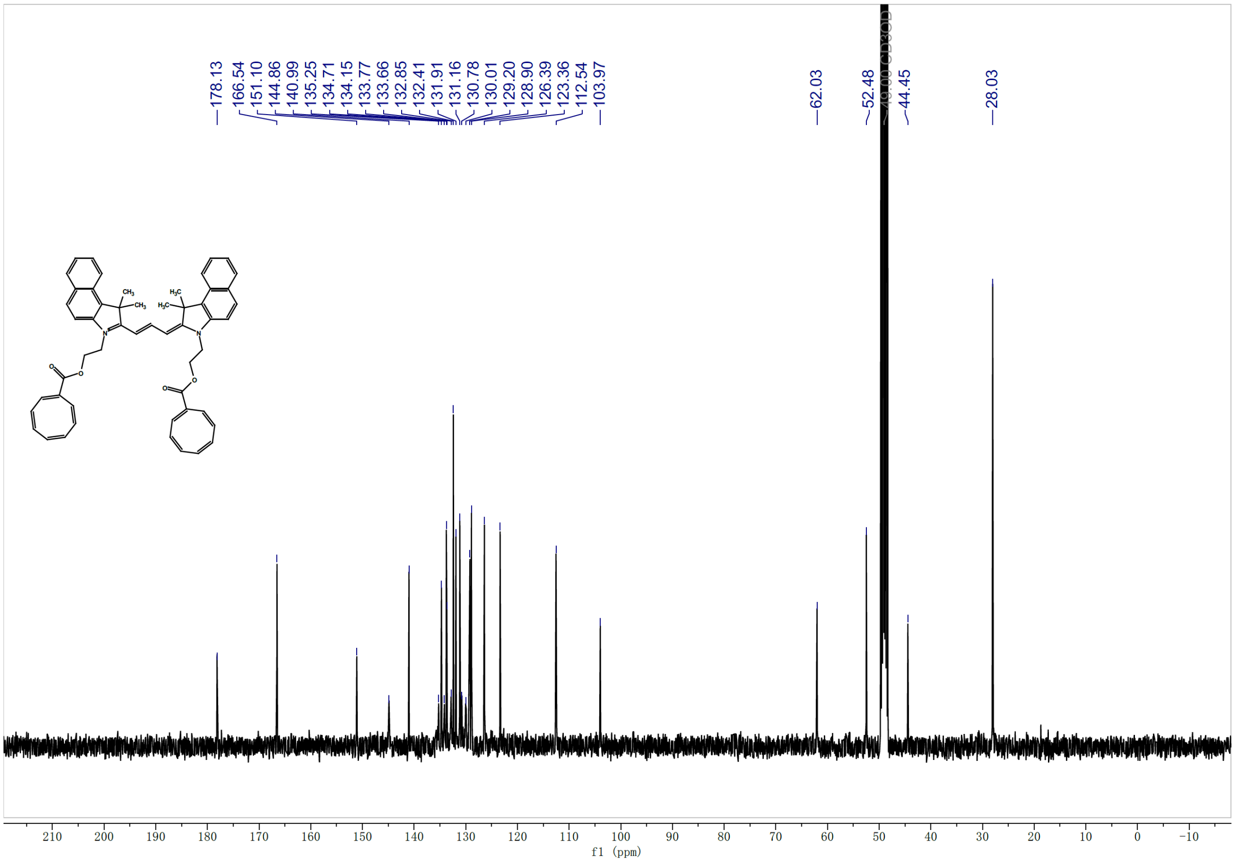

^{13}C NMR spectrum of compound PKMO

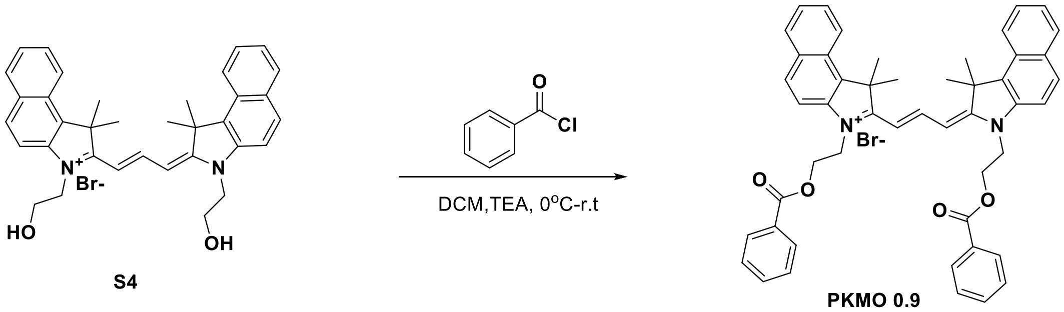

PKMO 0.9: To a mixture of compound **S4** (25.5 mg, 0.043 mmol) and triethylamine (21.6 mg, 0.213 mmol) in dry DCM (3.0 mL) was added dropwise the solution of benzoyl chloride (29.8 mg, 0.213 mmol) in dry DCM (0.5 mL) at 0°C under nitrogen. The mixture was stirred at room temperature for 16 h before concentrated under reduced pressure to afford the residue, which was purified by preparative TLC (DCM: MeOH=20:1) to afford crude **PKMO 0.9** (30.0 mg, 97%) as a purple solid. The solid was dissolved in DCM (1.5 mL) and added dropwise into diethyl ether (15 mL) slowly. The solid was collected and dried under reduced pressure to afford the **PKMO 0.9** (20.0 mg, 58%) as a purple solid.
^{1}H NMR (400 MHz, Methanol-*d*$_4$) δ 8.51 (t, *J* = 13.5 Hz, 1H), 8.21 (d, *J* = 8.6 Hz, 4H), 8.00 - 7.96 (m, 4H), 7.84 (dd, *J* = 8.4, 1.3 Hz, 4H), 7.73 (d, *J* = 8.8 Hz, 4H), 7.65 (t, *J* = 7.7 Hz, 2H), 7.49 (t, *J* = 7.6 Hz, 2H), 7.41 (td, *J* = 7.4, 1.4 Hz, 2H), 7.30 (t, *J* = 7.7 Hz, 4H), 6.52 (d, *J* = 13.4 Hz, 2H), 4.86 (t, *J* = 4.8 Hz, 4H), 4.73 (t, *J* = 5.0 Hz, 4H), 1.92 (s, 12H).
^{13}C NMR (101 MHz, Methanol-*d*$_4$) δ 178.06, 167.49, 150.78, 140.68, 134.70, 134.44, 133.54, 131.75, 131.06, 130.68, 130.40, 129.47, 129.09, 128.85, 126.32, 123.24, 112.30, 103.87, 62.40, 52.32, 44.65, 27.79.
MS (ESI) calcd for $C_{49}H_{45}N_2O_4^+$ [M]$^+$ 725.34, found 725.27.

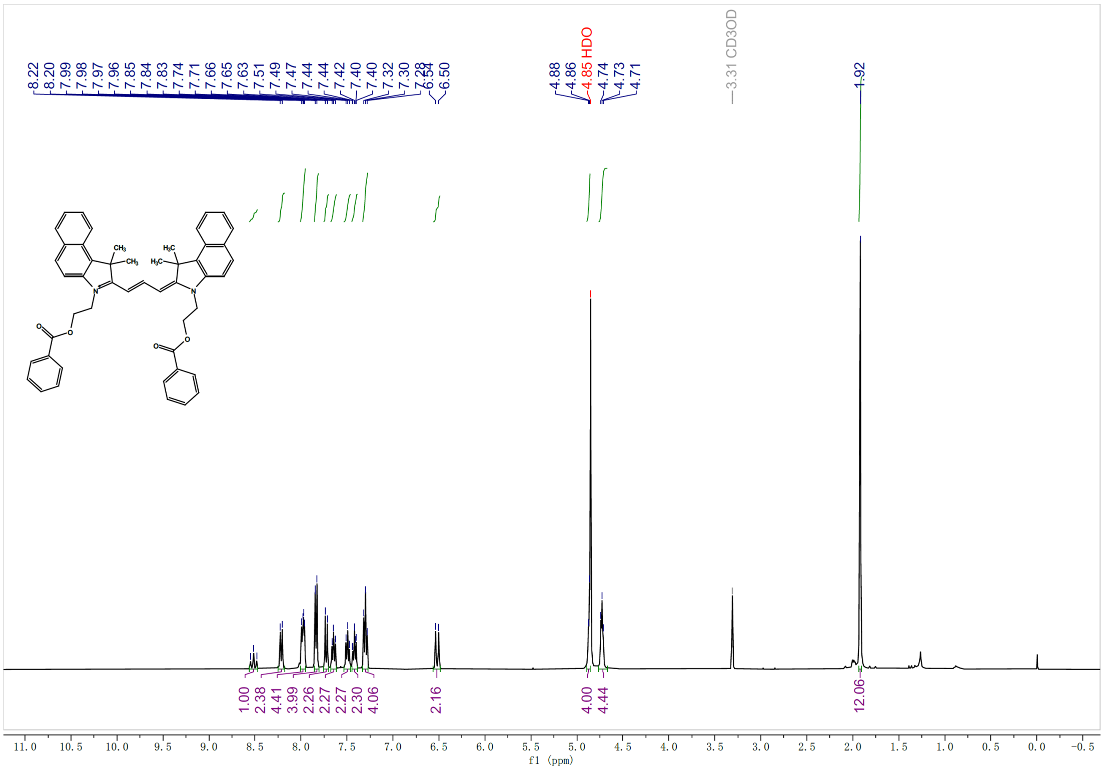

^{1}H NMR spectrum of compound PKMO 0.9

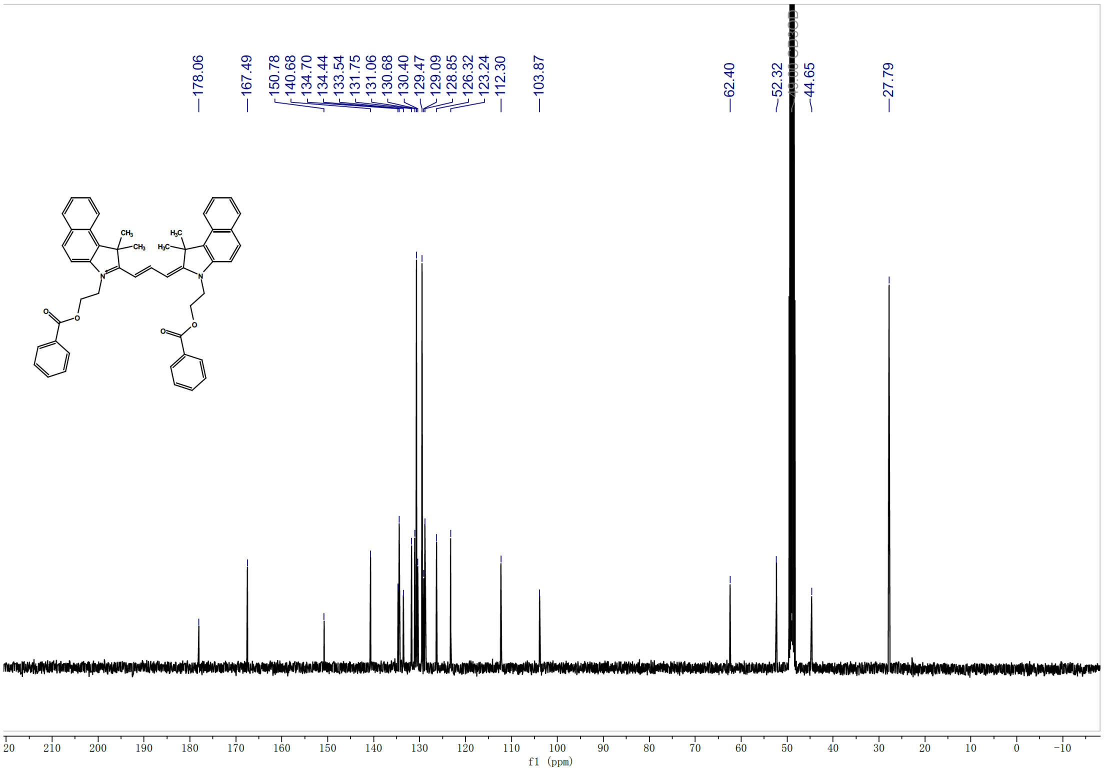

^{13}C NMR spectrum of compound PKMO 0.9

References

1. M. Lopalco, E. N. Koini, J. K. Cho, M. Bradley, Catch and release microwave mediated synthesis of cyanine dyes. *Org. Biomol. Chem.* **7**, 856-859 (2009).
2. R. W. Redmond, J. N. Gamlin, A Compilation of Singlet Oxygen Yields from Biologically Relevant Molecules. *Photochemistry and Photobiology* **70**, 391-475 (1999).
3. J. Gruber, T. Lampe, M. Osborn, K. Weber, RNAi of FACE1 protease results in growth inhibition of human cells expressing lamin A: implications for Hutchinson-Gilford progeria syndrome. *J. Cell Sci.* **118**, 689 - 696 (2005).
4. M. Fasshauer *et al.*, Essential Role of Insulin Receptor Substrate 1 in Differentiation of Brown Adipocytes. *Molecular and Cellular Biology* **21**, 319-329 (2001).
5. S. Kaech, G. Banker, Culturing hippocampal neurons. *Nat. Protoc.* **1**, 2406-2415 (2006).
6. L. Yang *et al.*, Transcriptional regulation of intermolecular Ca(2+) signaling in hibernating ground squirrel cardiomyocytes: The myocardin-junctophilin axis. *Proceedings of the National Academy of Sciences of the United States of America* **118**, e2025333118 (2021).
7. J. Zhang *et al.*, Red- and Far-Red-Emitting Zinc Probes with Minimal Phototoxicity for Multiplexed Recording of Orchestrated Insulin Secretion. *Angew. Chem. Int. Ed.* **60**, 25846-25855 (2021).
8. T. Stephan *et al.*, MICOS assembly controls mitochondrial inner membrane remodeling and crista junction redistribution to mediate cristae formation. *The EMBO Journal* **39**, e104105 (2020).
9. L. K. Schroeder *et al.*, Dynamic nanoscale morphology of the ER surveyed by STED microscopy. *J. Cell Biol.* **218**, 83-96 (2018).
10. Z. Chen, C. Jing, S. S. Gallagher, M. P. Sheetz, V. W. Cornish, Second-Generation Covalent TMP-Tag for Live Cell Imaging. *J. Am. Chem. Soc.* **134**, 13692-13699 (2012).
11. T. Stephan, A. Roesch, D. Riedel, S. Jakobs, Live-cell STED nanoscopy of mitochondrial cristae. *Sci. Rep.* **9**, 12419 (2019).
12. G. Lukinavičius *et al.*, A near-infrared fluorophore for live-cell super-resolution microscopy of cellular proteins. *Nat. Chem.* **5**, 132-139 (2013).
13. J. Bucevičius, G. Kostiuk, R. Gerasimaitė, T. Gilat, G. Lukinavičius, Enhancing the biocompatibility of rhodamine fluorescent probes by a neighbouring group effect. *Chem. Sci.* **11**, 7313-7323 (2020).
14. P. Isola, J. Y. Zhu, T. Zhou, A. A. Efros (2017) Image-to-Image Translation with Conditional Adversarial Networks. in *2017 IEEE Conference on Computer Vision and Pattern Recognition (CVPR)*, pp 5967-5976.
